# Pyrazine-Based Chemosensors for Cation Detection

**DOI:** 10.3390/molecules31193573

**Published:** 2026-10-08

**Authors:** Tatyana N. Moshkina, Emiliya V. Nosova, Galina N. Lipunova

**Affiliations:** Department of Organic and Biomolecular Chemistry, Ural Federal University, Mira St. 19, 620002 Ekaterinburg, Russia; tan.moshckina@yandex.ru (T.N.M.); lipunova@ios.uran.ru (G.N.L.)

**Keywords:** pyrazines, chemosensors, naked-eye sensors, absorption, fluorescence, metal ion detection, limit of detection

## Abstract

Pyrazine (1,4-diazine) and its annulated derivatives are promising platforms for colorimetric and fluorescent chemosensors of biologically and environmentally relevant metal cations, including Hg^2+^, Cu^2+^, Zn^2+^, Pb^2+^, Fe^3+^, Al^3+^, Co^2+^, Ni^2+^, Ag^+^ and Cr^3+^. Their sensing potential arises from the strongly electron-deficient pyrazine ring, low-lying unoccupied molecular orbitals, and considerable synthetic versatility. This review systematically summarizes the structural diversity, metal-ion recognition strategies, photophysical response mechanisms, and practical applications of pyrazine-derived small-molecule chemosensors, with emphasis on studies published between 2015 and 2026. Reported architectures include pyrazinamide, pyrazinehydrazide, 2-acetylpyrazine, aminopyrazine, hydrazinopyrazine, aryl(hetaryl)pyrazine derivatives, pyrazines fused with five-membered carbo/heterocycles, quinoxalines, and pyrido [2,3-*b*]pyrazines. The analysis shows that N,N,O-tridentate chelation, CHEF/CHEQ, PET, ICT, ESIPT, FRET and AIE mechanisms govern the sensing behavior, providing detection limits from nanomolar to micromolar levels and enabling naked-eye, test-strip, and live-cell imaging applications. It is concluded that sensing performance is primarily determined by the chelating motif and the substitution pattern, while selectivity towards Cu^2+^ and Fe^3+^ remains the main limitation. Promising directions include AIE-active long-wavelength emitters, portable smartphone-assisted platforms, MOF-based architectures, and DFT-guided rational design of next-generation pyrazine chemosensors.

## 1. Introduction

Pyrazine (1,4-diazine) and its annelated derivatives represent an important class of electron-deficient nitrogen heterocycles that possess a strong electron-accepting character, making them valuable building blocks for the construction of π-conjugated push–pull systems in combination with electron-donating groups [1,2]. The parent compound, pyrazine, has been extensively studied; its derivatives exhibit diverse biological activities and are widely employed in pharmaceutical chemistry, while also finding broad application in materials science as components of organic light-emitting diodes (OLEDs), dye-sensitized solar cells, and nonlinear optical materials [2,3,4]. Although the basicity of the pyrazine ring (pKa ~ 0.65) is significantly lower than that of pyridine (pKa ~ 5.2) [3], the presence of two nitrogen atoms in the 1,4-diazine ring enables protonation, participation in hydrogen bonding, and chelation with metal ions. These structural and electronic features, coupled with their favorable photophysical properties and convenient synthetic tunability, have positioned pyrazine derivatives as promising platforms for the development of chromogenic and fluorogenic chemosensors for metal cation detection [2,5,6]. In this context, luminescent materials incorporating pyrazine fragments have been the subject of recent comprehensive reviews [2,7], and the application of pyrazine-based sensors for the detection of metal ions in environmental and biological systems has attracted considerable research attention due to the growing demand for sensitive, selective, and cost-effective analytical tools for monitoring toxic metal contamination [3,8].

It is important to note that interest in pyrazine-containing compounds is not limited to classical small-molecule chemosensors. In recent years, a field related to the development of aggregation-induced emission (AIE) systems based on pyrazine has been actively advancing, which opens up new opportunities for the development of highly efficient sensor materials [9]. In parallel with this, extensive research has been devoted to the design of functionalized 1,4-diazenes for optoelectronic applications, including fluorescence sensing [3]. In particular, quinoxaline derivatives, which are benzo-annulated analogs of pyrazine, also demonstrate outstanding photophysical properties, making them promising platforms for the development of chromogenic and fluorogenic chemosensors capable of detecting a wide range of analytes [10]; a broad range of benzo-annulated azaheterocycles were analyzed as diverse fluorescent probes [11].

A complementary and rapidly expanding area of pyrazine-based sensing involves their incorporation into metal–organic frameworks (MOFs), where the pyrazine moiety often serves as an integral linker within the extended porous architecture. 2,3,5,6-Tetra(4-carboxyphenyl)pyrazine [12,13,14], tetraphenylpyrazine [15,16], 2,3,5,6-tetrakis[2-(4-carboxyphenyl)vinyl]pyrazine [17], 2,3,5,6-tetrakis-[4-[(1*H*-imidazole-1-yl)-methyl]phenyl]pyrazine [18] and tetrakis(4-(pyridin-4-yl)phenyl)pyrazine [19] are applied for design of MOF sensors. These hybrid materials leverage the intrinsic porosity, high surface area, and structural tunability of MOFs to achieve exceptional performance in fluorescence-based detection of various analytes, including metal ions [12,14], antibiotics [17], explosives [13,15,18], and even carcinoid biomarkers [19], often with remarkable sensitivity and selectivity. However, while such MOF-based sensors represent a powerful paradigm in sensing technology, the present review is focused on a different class of pyrazine derivatives: discrete small molecules and related oligomeric or polymeric architectures that function as chelating or coordinating ligands capable of directly interacting with metal cations, generating a distinct chromogenic or fluorogenic signal through well-defined molecular interactions. Most of the sensors discussed are discrete small molecules acting as bi-, tri-, or tetradentate ligands; however, a small number of conjugated polymers and copolymers are also included, because they exploit the same pyrazine-based coordination chemistry and the same photophysical response mechanisms as their small-molecule counterparts. Systems in which the pyrazine nitrogen acts as a monodentate donor are also discussed, as they illustrate the structural versatility of the scaffold.

Pyrazine-based small-molecule sensors differ from other optical sensing materials in several key aspects. Unlike MOFs and coordination polymers, they are discrete, solution-processable, and structurally well-defined. Unlike bipyridines, terpyridines, or phenanthrolines, they contain two adjacent nitrogen donors in a single electron-deficient ring, which lowers the π* orbitals, promotes ICT, and enables N,N,O-tridentate chelation upon functionalization. Unlike quinoxalines and pyrido[2,3-*b*]pyrazines, the parent pyrazine core is smaller, more polar, and synthetically more accessible, while annulation shifts emission to longer wavelengths. Finally, unlike conventional fluorophores (rhodamine, BODIPY, pyrene), the pyrazine moiety acts simultaneously as a coordination site, an electron acceptor, and a modulator of CHEF, CHEQ, PET, ICT, ESIPT, and AIE pathways. This dual role—receptor and electronic modulator—distinguishes pyrazine derivatives from many other sensing platforms. It should be noted that the term “small-molecule sensors” is used here in a broad sense: it encompasses discrete molecular receptors as well as the small number of conjugated polymers and copolymers that share the same pyrazine-based coordination motif. These polymeric systems are included because they extend the same design principles—pyrazine as an electron-deficient coordination site and electronic modulator—to macromolecular platforms, and they illustrate the generality of the mechanisms discussed in this review.

**Figure 1:**
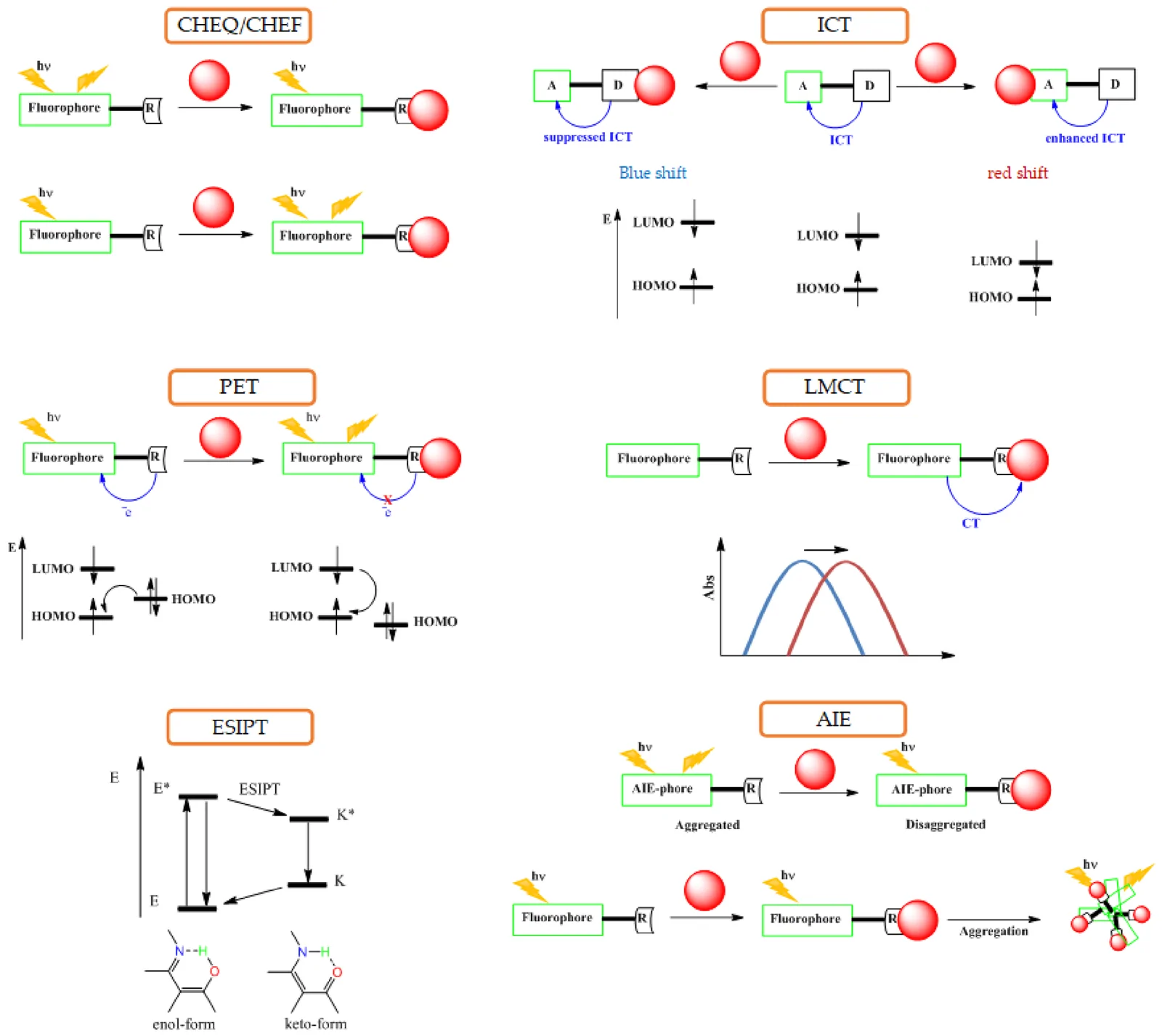
Schematic representation of the principal sensing mechanisms operating in pyrazine-based chemosensors: CHEF, CHEQ, PET, ICT, ESIPT, AIE, and ligand-to-metal charge transfer (LMCT). Color and cymbol coding: the sphere shown in red represents the analyte; the yellow lightning bolt represents the photons (hv) absorbed and emitted; A—acceptor group; D—donor group; R—receptor group. The schemes illustrate the general photophysical pathways rather than specific molecular structures; pyrazine-based sensors for each mechanism are discussed in Section 2 and Section 3 and summarized in Table 1.

Comprehensive surveys dedicated to the detection of various biologically and environmentally relevant metal ions, including copper, zinc, aluminum, mercury, nickel, iron, cadmium, and palladium, have demonstrated that Schiff bases, hydrazones, and related functionalized derivatives represent some of the most common and effective ligand scaffolds for the construction of efficient chemosensors for cations [20,21]. These structural motifs, particularly those containing imine (C=N) and amide functionalities, provide multiple coordination sites that enable strong and selective binding to metal cations. Metal coordination may generate an optical response in the form of spectral and/or color changes, fluorescence enhancement/quenching, or an emission wavelength shift. The nature of this response is determined by the photophysical process operating in the sensor, with reported examples operating through chelation-enhanced fluorescence (CHEF) or quenching (CHEQ), photoinduced electron transfer (PET), intramolecular charge transfer (ICT), excited-state intramolecular proton transfer (ESIPT), Förster resonance energy transfer (FRET), and AIE [20,22,23,24,25]. The most frequently encountered sensing mechanisms in pyrazine-based chemosensors—CHEF/CHEQ, PET, ICT, ESIPT, and AIE, and LMCT—are schematically depicted in Figure 1. The prevalence of such ligands in the literature underscores their significance as privileged structures in the field of cation chemosensing, offering the advantages of straightforward synthetic accessibility, tunable photophysical properties, and versatile coordination modes [21].

Heavy metals are ubiquitous environmental pollutants characterized by high atomic mass and density (typically >5 g/cm^3^). Common metal ions of concern include mercury, nickel, cadmium, lead, chromium, cobalt, iron, copper, zinc, silver and manganese [26]. Unlike organic contaminants, these elements are essentially non-biodegradable and therefore persistently accumulate in soils, waters and the atmosphere as a result of industrial, agricultural and pharmaceutical activities [27]. While some heavy metals occur naturally in the environment, anthropogenic emissions have substantially increased their concentrations, resulting in toxic effects on human health, fauna and flora. Consequently, it is essential to monitor metal concentrations in industrial emissions and in environment to prevent harmful impacts on living organisms and human life.

Mercury (Hg) is a hazardous element of both natural and anthropogenic origin, present in air, water and soil. It has been recognized as a global pollutant with a toxic, mobile, and persistent nature [28]. All forms of mercury are harmful, including inorganic species such as metallic mercury, mercury vapor (Hg^0^), mercurous (Hg^+^, as the dimeric Hg_2_^2+^ cation) and mercuric (Hg^2+^) salts, as well as organic compounds in which mercury is bonded to methyl, ethyl, phenyl, or related groups [29]. Toxic effects of Hg are associated with neurological disorders, cognitive impairment, developmental delays, cardiovascular damage, and high-level exposure may be fatal; overall toxicity depends on the chemical form, dose and rate of exposure [27,29]. The mechanisms of mercury toxicity are complex and multifactorial; however, direct binding to protein thiol groups plays a key role in microtubule disruption, protein deactivation and consequent metabolic and redox imbalance in cells [30].

Lead (Pb) stands out among heavy metal ions as particularly dangerous for both human health and marine life. Soluble or sparingly water-soluble Pb^2+^ salts can readily enter biological systems through contaminated water and soil, exerting significant negative effects on the health of organisms. Lead has no physiological role in the body, and even low levels can cause toxicity. Lead toxicity disrupts the functions of the digestive, nervous, respiratory, and reproductive systems, among others, and interferes with enzymatic activity by preventing enzymes from performing their normal functions. Of all organs, the nervous system is the most affected target in lead toxicity, both in children and adults; excessive accumulation of Pb^2+^ not only seriously damages the central nervous system but has also been linked to neurodegenerative disorders such as Alzheimer’s disease [31]. Moreover, Pb^2+^ disrupts normal DNA transcription processes and causes abnormalities in bone development [32].

Copper (Cu) is an essential trace element with critical roles in physical and mental health, primarily as a cofactor in endogenous enzymes. Although metallic copper is generally not highly toxic, several copper salts (e.g., CuSO_4_, CuCO_3_ and copper sub-acetate) can cause acute and chronic poisoning through inhalation, ingestion or dermal exposure. Excess Cu^2+^ accumulates mainly in the liver, ultimately affecting the nervous, reproductive and endocrine systems and connective tissues, and high-dose exposure may lead to severe gastrointestinal symptoms, neurological complications and even death [33]. Human exposure to Cu^2+^ may increase through contaminated drinking water and environmental sources; therefore, monitoring copper levels in water and other environmental sources is necessary [34]. Real-life applications of organic sensors for Cu^2+^ detection, including environmental monitoring of water quality, point-of-care diagnostics, and quality control in industrial processes, have been extensively reviewed [35].

Nickel (Ni) is another essential trace element, and Ni^2+^ is the most common oxidation state in biological systems. Its use has increased considerably with the expansion of modern technologies, leading to greater human exposure to nickel compounds, which can cause a variety of adverse, threshold-dependent health effects [36]. The most important and frequent effects include nickel allergy in the form of contact dermatitis, lung fibrosis, cardiovascular and kidney diseases, and lung and nasal cancers [37,38].

Zinc chloride (ZnCl_2_) is a versatile reagent widely used in industrial organic synthesis as a catalyst, dehydrating agent, and precursor for functional materials. Beyond process control, monitoring ZnCl_2_ is important for environmental protection because of its toxicity, and for biological studies, since excessive zinc in its chloride form may exert corrosive and damaging effects despite the essential role of Zn^2+^ ions in living systems [39]. Although numerous fluorescent probes for Zn^2+^ ions have been developed, most are not selective for the chloride salt form, highlighting the need for systems capable of detecting ZnCl_2_ specifically [40].

Silver (Ag) is an important industrial and biomedical metal, and its ionic form is widely used in photography, agriculture, electronic imaging, medical devices, and the pharmaceutical industry, making its detection and monitoring highly significant. Ag^+^ can contaminate soil, water, air and food and may accumulate in the human body through the food chain. By coordinating with thiol, imidazole, amino and carboxyl groups, Ag^+^ can cause developmental delays, skin damage, and toxicity to the kidney, liver, and central nervous system [41].

Iron (Fe) is an essential metal in humans and plays a wide range of biological functions. It serves as a cofactor for several enzymes involved in redox reactions because it can switch between ferrous Fe^2+^ and ferric Fe^3+^ states [42]. Maintaining appropriate iron levels is vital for human health, as both deficiency and overload can lead to disorders such as anemia and hemochromatosis and may damage the liver, heart, and endocrine glands. Iron enters environments through mining, industrial effluents, pipe corrosion, and manufacturing processes. Disruptions in iron levels can adversely affect ecosystems, and excessive human exposure may cause serious health effects [43].

Chromium (Cr) mainly exists in the +3 and +6 oxidation states. Cr^3+^ is less toxic and less mobile, and it is considered an essential trace element in biological systems; however, prolonged exposure may still have adverse health effects. Cr^3+^ can be easily oxidized to Cr^6+^, which is genotoxic, hemotoxic, and carcinogenic. Owing to its smaller size and strong oxidizing properties, Cr^6+^ can readily penetrate biological cell membranes, leading to various disorders [44].

Cobalt (Co) is an essential trace element whose biological significance in humans is primarily associated with its incorporation into vitamin B_12_ (cobalamin). Through cobalamin-dependent enzymes, it indirectly contributes to erythropoiesis and DNA synthesis and supports normal neurological function and the metabolism of lipids and carbohydrates. Excessive cobalt exposure, however, may result in cardiotoxicity and dysfunction of the thyroid, gastrointestinal, and nervous systems [45]. Systematic monitoring of cobalt, particularly in soils, is essential for assessing environmental pollution and protecting the quality of feed, food, and human and animal health [46].

Aluminum (Al) is one of the most abundant metals in the Earth’s crust and is widely used in industry and consumer products [47,48]. Due to its extensive use, it can enter food, water, and air, accumulate in the human body, and impair biological functions, especially under prolonged exposure [48]. Because excessive Al^3+^ exposure has been linked to neurotoxicity and several neurological disorders, the development of sensitive and selective probes for Al^3+^ detection in both environmental and biological systems is important [49,50].

Beyond their application in cation sensing, pyrazine-based small-molecule chemosensors have also been developed for the detection of a variety of other important analytes. For instance, a simple pyrazine-2-carboxamide derivative was reported as a highly selective colorimetric sensor for the detection of phenylalanine in blood plasma, exhibiting a distinct color change from colorless to bright green upon analyte recognition [51]. Furthermore, annulated pyrazine derivatives, particularly those incorporating AIE-active tetraphenylethylene moieties, have been demonstrated to serve as effective fluorescent probes for the selective detection of nitroaromatic compounds (NACs), including explosives such as TNT, with high sensitivity and fast response times in aqueous media [52]. Other studies have explored pyrazine-derived chemosensors for the detection of anions, such as cyanide and fluoride, through distinct mechanisms including nucleophilic addition and deprotonation [53]. Related quinoxaline compounds (benzo[*b*]pyrazines) were analyzed as chromogenic and fluorogenic chemosensors for fluoride, cyanide, acetate, and phosphate anions, in biomolecular applications [54]. However, as the present review is dedicated to pyrazine-based small-molecule sensors for metal cations, these examples involving alternative analytes are beyond the scope of this work and will not be discussed herein.

**Figure 2:**
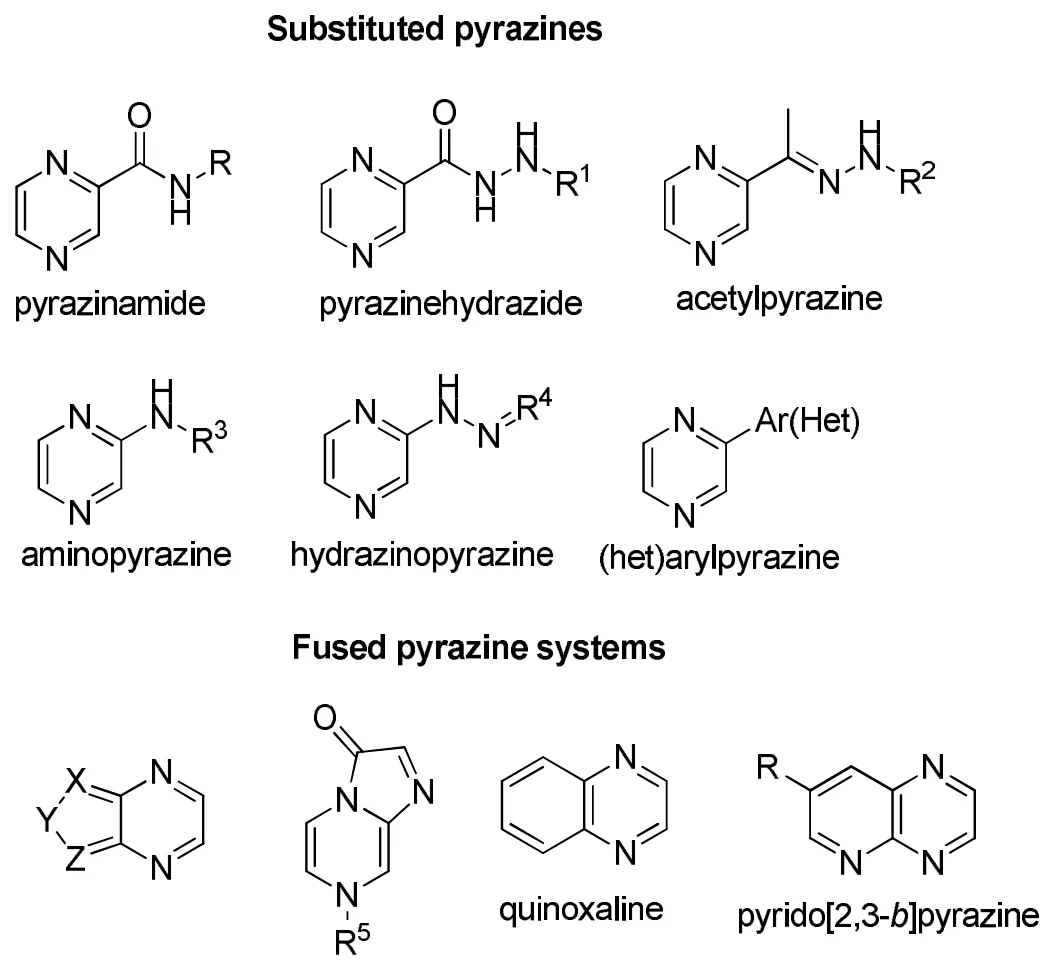
Types of pyrazines reported as chemosensors.

The molecular structures of sensors discussed herein are systematically presented in Figure 2.

This communication provides a comprehensive overview of the pyrazine-based optical sensors to metal cation developed mostly from 2015 to 2026, covering discrete small molecules as well as a small number of conjugated polymers and copolymers that exploit the same pyrazine-based coordination chemistry. Readers can quickly grasp both the recent advances and the existing gaps in the field of specific material recognition, facilitating them to create more effective pyrazine sensors.

## 2. Substituted Pyrazines

### 2.1. Pyrazinamide and Pyrazinehydrazide Derivatives

Pyrazine carboxamides represent an important class of pyrazine-based chemosensors because the combination of the ring nitrogens and the amide group enables diverse coordination chemistry. N-substituted pyrazine-2-carboxamides can form a pincer-like cavity that chelates metal ions, often giving stable, planar complexes.

The sensors discussed in this section operate predominantly through CHEF and CHEQ, with PET and ICT playing auxiliary roles. A common structural feature of pyrazinamide- and pyrazinehydrazide-based receptors is the formation of N,N,O-tridentate chelating pockets, in which the amide or hydrazide carbonyl oxygen, the imine nitrogen, and one of the pyrazine ring nitrogens participate in metal binding. The nature of the photophysical response—“turn-on” or “turn-off”—is often influenced by the electronic configuration of the coordinated cation: diamagnetic ions such as Zn^2+^, Al^3+^ and Ag^+^ typically activate CHEF, whereas paramagnetic Cu^2+^ (d^9^) and Fe^3+^ (d^5^) frequently induce CHEQ through efficient non-radiative deactivation pathways.

**Figure 3:**
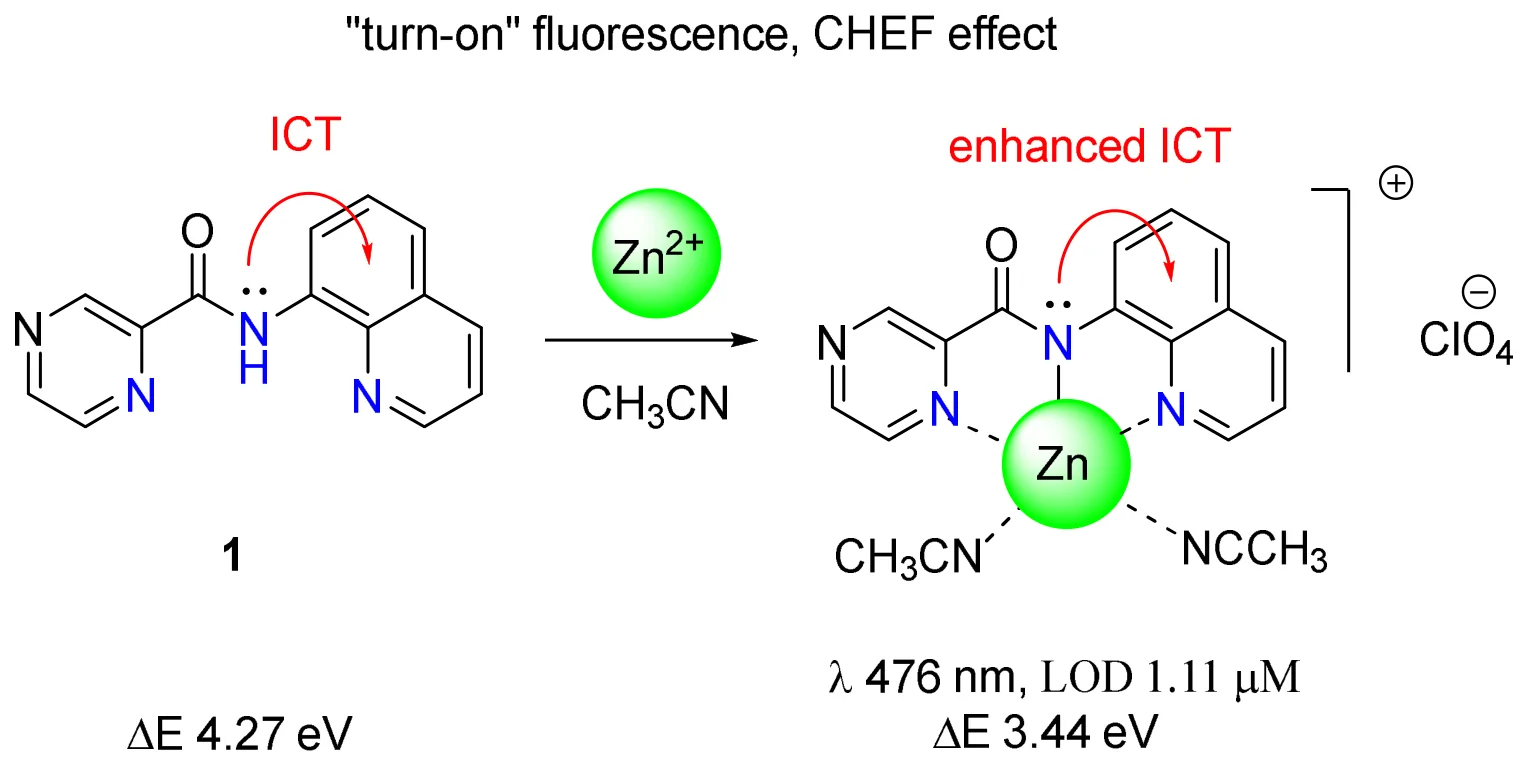
Molecular structure of *N*-(quinoline-8-yl)pyrazine-2-carboxamide sensor **1** and proposed interaction mode with Zn^2+^ upon addition of Zn(ClO_4_)_2_ × 6H_2_O. Color coding: the proposed detection mechanism is shown in red; nitrogen atoms involved in metal coordination are shown in blue; the sphere shown in color represents the detectable cation in this and subsequent figures.

A chemosensor **1**, based on pyrazine-2-carboxamide covalently linked to a quinoline fragment (Figure 3), showed “turn-on” fluorescent response to Zn^2+^ in acetonitrile among 13 other tested metal ions, including Na^+^, K^+^, Mg^2+^, Ca^2+^, Al^3+^, Co^2+^, Cd^2+^, Cr^3+^, Fe^2+^, Hg^2+^, Ni^2+^, Mn^2+^, as well as Cu^2+^ [55]. Compound **1** also demonstrated selective colorimetric (naked-eye) detection of Cu^2+^, with a color change from colorless to yellow and a bathochromic shift in the long-wavelength absorption maximum from 328 nm to 378 nm, attributed to an intense charge-transfer transition. It should be emphasized that the colorimetric response to Cu^2+^ originates from an intense LMCT/ICT transition rather than from CHEF, which operates only for Zn^2+^. Thus, sensor 1 exemplifies a dual-mechanism probe in which the same receptor operates through CHEF towards Zn^2+^ and through ICT/LMCT towards Cu^2+^. The color change remains stable even in the presence of competing metal ions, indicating high selectivity for Cu^2+^.

The fluorescence enhancement of pyrazine-2-carboxamide **1** at 476 nm (λ_ex_ = 360 nm) upon addition of Zn(ClO_4_)_2_ × 6H_2_O was explained by the CHEF mechanism: coordination of Zn^2+^ induces the deprotonation of the ligand and formation of a more rigid, extended π-conjugated system, that suppresses non-radiative decay pathways. Fluorescence and UV–vis titrations, a Job’s plot, ESI-MS (*m*/*z* 396.74), and density functional theory (DFT) calculations support a 1:1 binding stoichiometry, assigned as [Zn**1**(NCCH_3_)_2_]^+^, with N,N,N-coordination of the ligand **1** (Figure 3). The reported association constants and limit of detections (LODs) for Zn^2+^ were 3.837 × 10^4^ M^−1^ and 1.11 µM, respectively; for Cu^2+^, the reported values were 7.352 × 10^7^ M^−1^ and 14.8 µM.

DFT and time-dependent DFT (TD-DFT) calculations (B3LYP/6-31G(2d,p)/LANL2DZ) of **1** and its Zn^2+^ and Cu^2+^ complexes in acetonitrile are consistent with the experimental observations: the calculated emission for the **1**-Zn^2+^ complex (482 nm) closely matches the experimental emission (476 nm), and the calculated absorption for the **1**-Cu^2+^ complex shifts from 326 nm (free ligand) to 386 nm upon complexation, in agreement with the experimental shift to 378 nm. It should be noted that the TD-DFT transition energies were obtained with a specific functional and basis set, and their agreement with experiment reflects the quality of the computational model rather than a direct equivalence between orbital and spectroscopic energies. These results are consistent with a binding mode in which the deprotonated ligand (L^–^) coordinates the metal center while acetonitrile molecules complete the coordination sphere [55]. The reversible change in emission upon sequential addition of Zn^2+^ and SCN^−^ was exploited to construct an INHIBIT logic gate.

**Figure 4:**
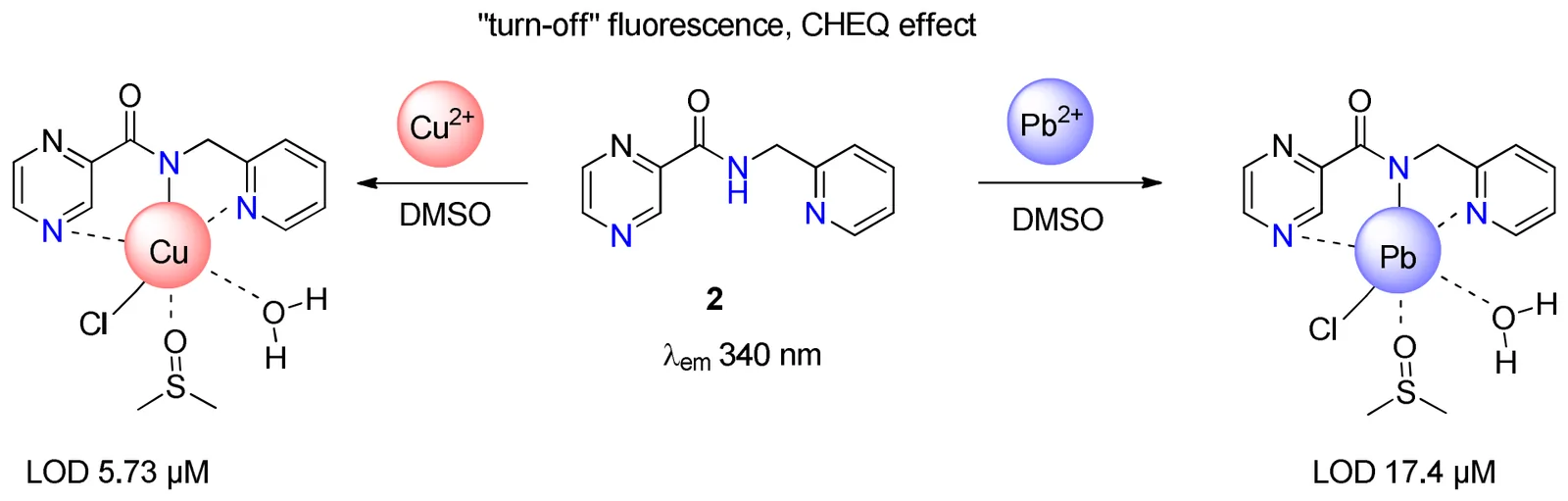
Molecular structure of a pyrazine-2-carboxamide-based fluorescent sensor **2** and proposed interaction mode with Cu^2+^ or Pb^2+^ upon addition of CuCl_2_ × 2H_2_O or PbCl_2_, respectively. Color coding: nitrogen atoms involved in metal coordination are shown in blue.

Ravandi et al. [56] reported *N*-(pyridine-2-ylmethyl)pyrazine-2-carboxamide **2** as a “turn-off” fluorescent probe for selective detection of Cu^2+^ and Pb^2+^ in aqueous media (Figure 4). The authors systematically evaluated key analytical parameters, including pH, response time, and reversibility. Upon addition of Cu^2+^ and Pb^2+^, sensor **2** exhibited fluorescence quenching at 340 nm (λ_ex_ = 281 nm). Stern–Volmer analysis of the titration data yielded quenching constants K_sv_ = 2.30 × 10^4^ M^−1^ for Cu^2+^ and 1.10 × 10^4^ M^−1^ for Pb^2+^. Limits of detection and quantification (LOD and LOQ) were determined as 5.73/9.62 µM for Cu^2+^ and 17.4/29.2 µM for Pb^2+^. Job’s plot, corroborated by ESI-MS and FT-IR spectroscopy, indicated a 1:1 binding stoichiometry for both metal complexes. The observed fluorescence quenching was attributed to CHEQ. For Cu^2+^, paramagnetic quenching (d^9^ configuration) via enhanced intersystem crossing (ISC) is likely dominant; for Pb^2+^, a heavy-atom effect contributes to efficient non-radiative decay. Sensor **2** displayed a rapid response (<1 min), high selectivity over 18 competing metal ions, and greater sensitivity toward Cu^2+^ than toward Pb^2+^. Practical applicability was demonstrated in drinking water, human blood plasma, and on paper test strips. In addition, carboxamide **2** showed reversible “Write–Read–Erase–Read” memory behavior using ethylenediaminetetraacetic acid (EDTA) and Cu^2+^ as chemical inputs, with the fluorescence intensity at 354 nm as the output signal, highlighting its potential for molecular memory applications.

**Figure 5:**
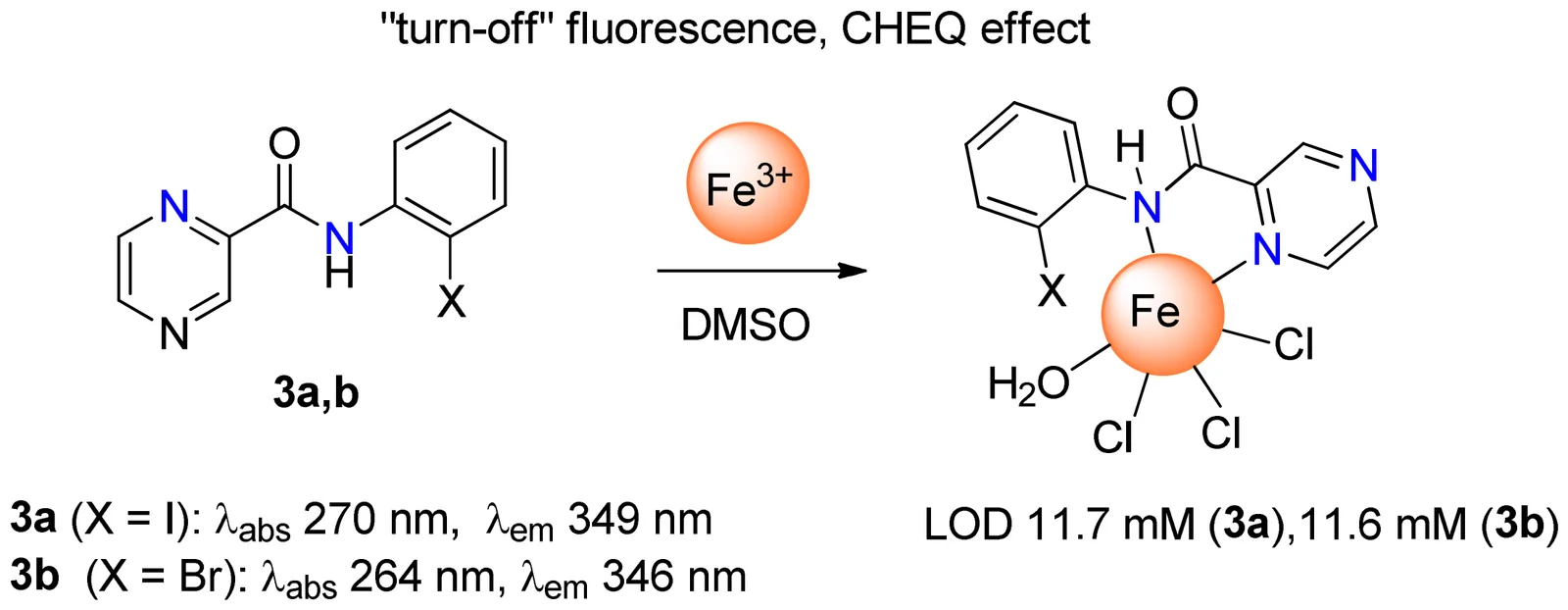
Molecular structure of *N*-(2-halophenyl)pyrazine-2-carboxamides **3a**,**b** and proposed interaction mode with Fe^3+^ upon addition of FeCl_3_ × 6H_2_O. Color coding: nitrogen atoms involved in metal coordination are shown in blue.

Extending this series, Ravandi et al. [57] described two *N*-(2-halogenophenyl)pyrazine-2-carboxamides (X = I, Br) **3a,b** as highly selective “turn-off” fluorescent and colorimetric sensors for Fe^3+^ in DMSO (Figure 5). The free iodo and bromo ligands absorb at 270 nm (X = I) and 264 nm (X = Br) and emit at 349 nm (λ_ex_ = 268 nm) and 346 nm (λ_ex_ = 284 nm), respectively. Addition of Fe^3+^ induces pronounced fluorescence quenching and an increase in absorption intensity. This behavior was attributed to CHEQ, driven by the paramagnetic nature of Fe^3+^ (d^5^ configuration) that facilitates the non-radiative deactivation of the excited state. Selectivity was confirmed in the presence of a broad range of competing cations, including monovalent Na^+^, K^+^, Ag^+^, bivalent Mg^2+^, Ca^2+^, Ba^2+^, Mn^2+^, Sn^2+^, Co^2+^, Cd^2+^, Pb^2+^, Ni^2+^, Cu^2+^, Hg^2+^, Zn^2+^, as well as trivalent Al^3+^, Cr^3+^, with minimal interference.

Job’s continuous variation method and ESI-MS (*m*/*z* 503.19, assigned to [**3a**·Fe(H_2_O)Cl_3_]) supported a 1:1 stoichiometry for the Fe^3+^ complex. From fluorescence titration, LODs were determined as 11.7 μM (iodo) and 11.6 μM (bromo). Association constants obtained by Benesi–Hildebrand analysis were K_a_ = 4.90 × 10^4^ M^−1^ and 1.75 × 10^3^ M^−1^ for **3a** and **3b**, respectively. Stern–Volmer analysis (K_SV_ = 4.24 × 10^5^ M^−1^ and 7.59 × 10^4^ M^−1^) indicates a predominantly static quenching mechanism. The binding was reversible upon addition of EDTA over at least eight cycles. The iodine-containing sensor **3a** was successfully applied for Fe^3+^ determination in real water samples (drinking, tap, and river water) and on test strips, where complexation with Fe^3+^ produced a visible color change from colorless to bright yellow, demonstrating its potential for rapid water-quality monitoring.

**Figure 6:**
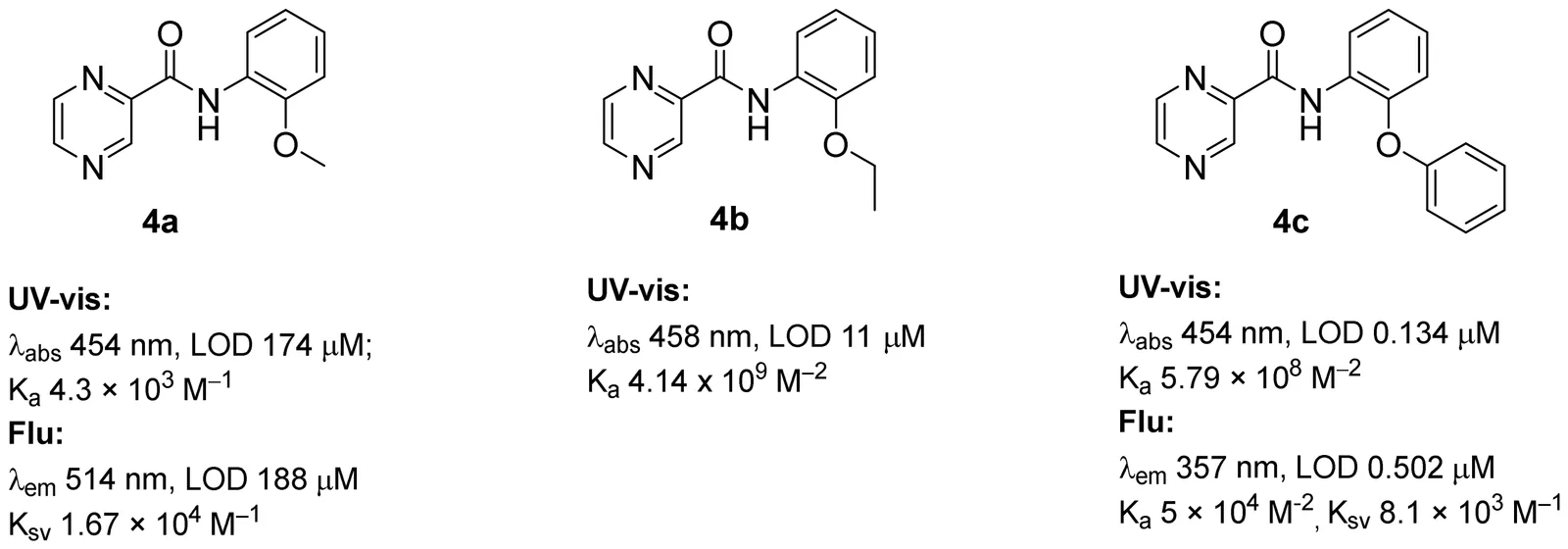
Molecular structure of pyrazine-2-carboxamides-based sensors **4a**–**c** and their sensing properties toward Cu^2+^.

Heydari et al. developed *N*-(2-alkoxyphenyl)- and *N*-(2-phenoxyphenyl)pyrazine-2-carboxamides **4a**–**c** (Figure 6) as chemosensors for Cu^2+^ [58,59,60]. Upon the addition of Cu^2+^, initially colorless solutions of **4a**–**c** turned pale yellow under visible light and dark yellow under UV illumination (fluorescence studied for **4a** and **4c** derivatives), with no observable interference from 18 competing metal ions. Spectral studies revealed an enhancement of the absorption band and the appearance of a new low-intensity band at ~454–458 nm, attributed to d–d transitions and partial LMCT, consistent with complex formation. The fluorescence quenching observed around 514 nm for **4a** (λ_ex_ = 283 nm) and 359 nm for **4c** (λ_ex_ = 280 nm) was triggered by non-radiative relaxation due to the paramagnetism of Cu^2+^ (d^9^ configuration). This quenching mechanism is consistent with CHEQ.

Variation in the ortho substituent on the phenyl ring (from methoxy to ethoxy and phenoxy) enabled tuning of the analytical performance, with reported limits of detection spanning from the millimolar to the micromolar range across the series (Figure 6).

**Scheme 1:**
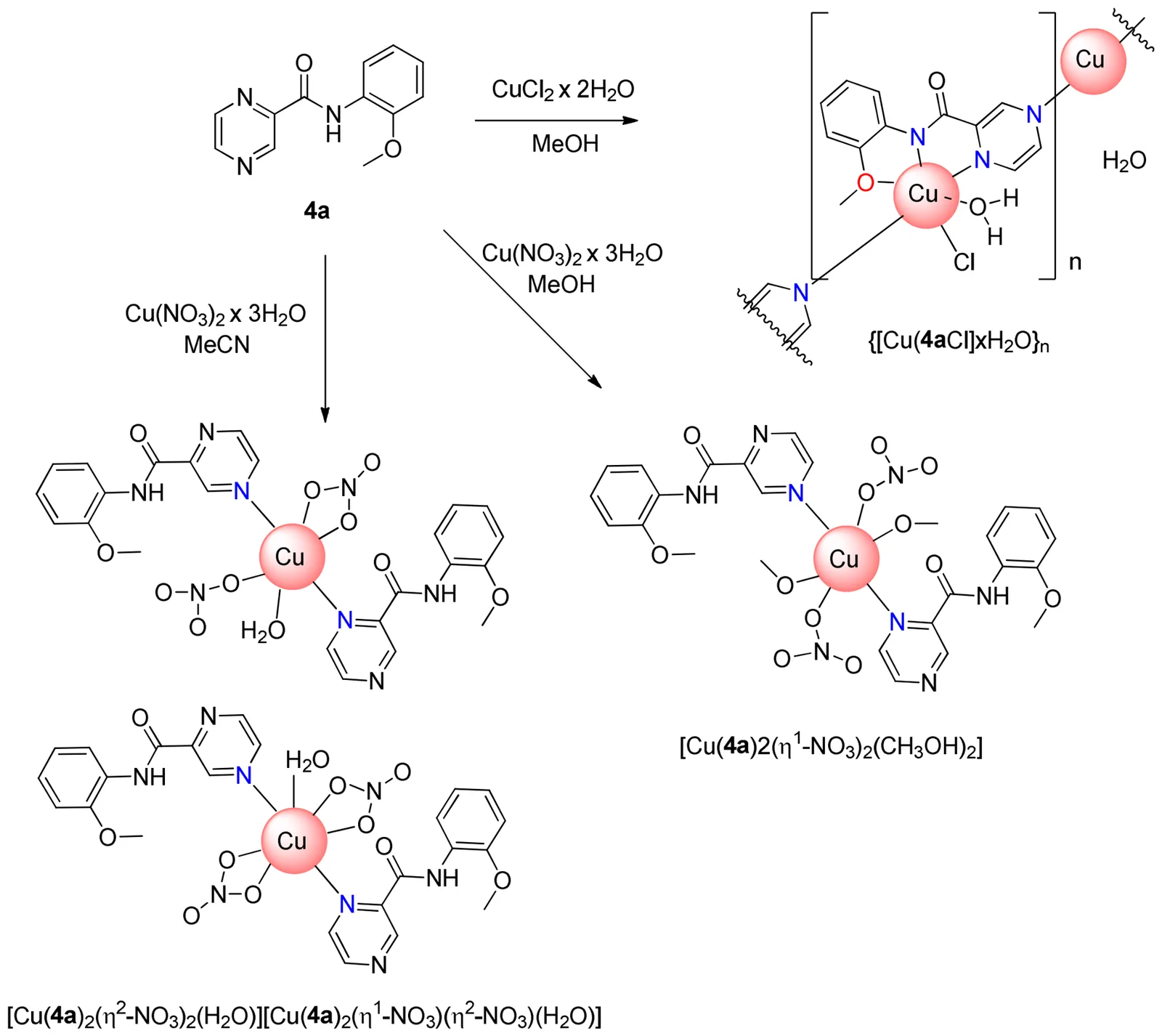
Synthesis of **4a**-Cu^2+^ complexes. Color coding: nitrogen atoms involved in metal coordination are shown in blue; oxygen atom involved in metal coordination is shown in red.

The coordination behavior of ligands **4a**–**c** toward Cu^2+^ depends on the copper salt, stoichiometry, and solvent, leading to different structural motifs [58,59,60]. For *N*-(2-methoxyphenyl)pyrazine-2-carboxamide **4a**, reaction with CuCl_2_×2H_2_O (1 equiv.) in MeOH in the presence of Et_3_N afforded a one-dimensional polymeric complex {[Cu(**4a**Cl)]·H_2_O}_n_ (Scheme 1). In this structure, the deprotonated ligand **4a** coordinates in a tridentate N,N,O fashion and bridges adjacent Cu^2+^ centers via the pyrazine nitrogen, generating ladder-like chains with distorted square-pyramidal geometry. In contrast, using 2 equiv. of Cu(NO_3_)_2_ × 3H_2_O in methanol or acetonitrile yielded mononuclear spacers [Cu(**4a**)_2_(η^1^-NO_3_)_2_(CH_3_OH)_2_] or [Cu(**4a**)_2_(η^2^-NO_3_)_2_(H_2_O)_2_] [Cu(**4a**)_2_(η^2^-NO_3_)(η^1^-NO_3_)(H_2_O)] (Scheme 1), respectively, in which two ligands **4a** binds in a monodentate mode through the pyrazine nitrogen, while nitrate anions and solvent molecules complete distorted octahedral or pentagonal–bipyramidal coordination spheres. These results highlight the conformational flexibility of **4a** and the strong influence of reaction conditions on the final coordination mode [58]. For the ethoxy derivative **4b,** addition of Cu(NO_3_)_2_ × 3H_2_O to **4b** (2:1 equiv.) in acetonitrile led to formation of a mononuclear complex described as [Cu(**4b**)_2_(η^2^-NO_3_)_2_(H_2_O)] with spectroscopic and crystallographic data supporting this formulation [59].

**Scheme 2:**
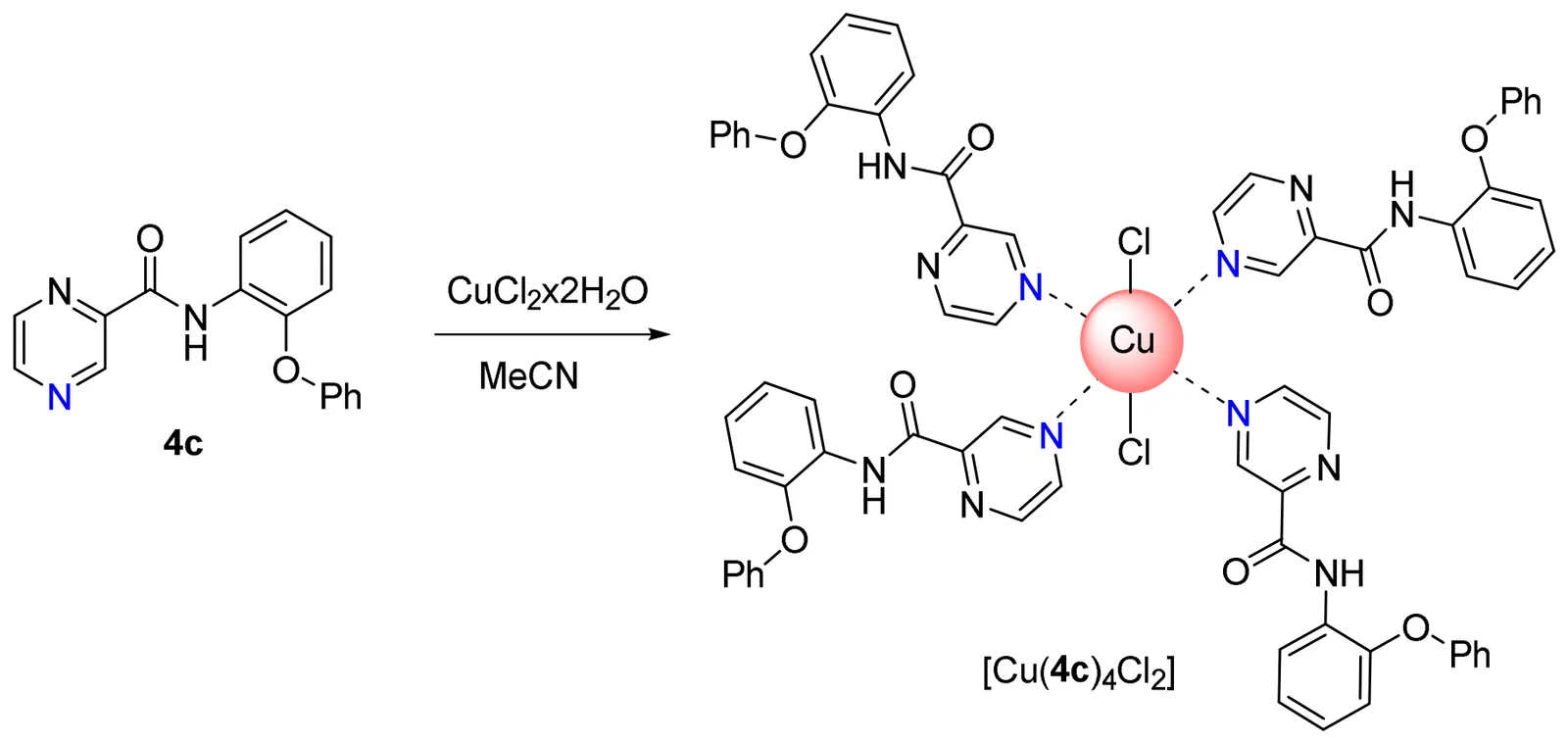
Synthesis of **4c**-Cu^2+^ complex. Color coding: nitrogen atoms involved in metal coordination are shown in blue.

In case of phenoxy derivative **4c**, reaction with CuCl_2_ × 2H_2_O (4 equiv.) in acetonitrile afforded [Cu(**4c**)_4_Cl_2_] (Scheme 2), as confirmed by single-crystal X-ray diffraction. XRD revealed a six-coordinate distorted octahedral geometry of **4c** with the Cu^2+^ ion at an inversion center, four monodentate **4c** ligands coordinated through pyrazine nitrogen atoms, and two chloride ligands. A pronounced Jahn–Teller distortion was observed, with elongated axial Cu–N bonds (2.543(3) Å) and shorter equatorial Cu–N bonds (2.056(3) Å).

Job’s plot analyses based on fluorescence titrations indicated **L**:Cu^2+^ stoichiometries of 2:1 for **4b** and 4:1 for **4c**, in agreement with the crystallographically characterized complexes [59,60]. For **4a**, the stoichiometry depended on the copper salt and solvent, as described above; in some titration experiments the exact Cu^2+^ salt and conditions were not specified in the original reports [58].

Across the series **4a**–**c**, the sensors exhibited a rapid response (less than 1 min), good reversibility with EDTA (three to five cycles), stability for at least one week, and effective performance in a broad pH range (including the pH of environmental water sources and biological fluids, pH ≈ 6). Together, these properties establish *N*-(2-alkoxyphenyl)- and N-(2-phenoxyphenyl)pyrazine-2-carboxamides **4a**–**c** as selective, reversible, and practically useful Cu^2+^ probes for both solution-phase and solid-state (test-strip) applications. The fluorescence quenching observed for **4a–c** is attributed to CHEQ arising from the paramagnetic d^9^ configuration of Cu^2+^, which promotes efficient non-radiative relaxation via enhanced ISC.

Hydrazones derived from pyrazinecarboxylic acid hydrazide and aryl/hetaryl aldehydes (Het–CH=N–NH–C(O)–Pyraz) are widely used ligands owing to their straightforward synthesis via Schiff-base condensation and versatile coordination modes. In the absence of additional pyridine-type nitrogen atoms or hydroxy/alkoxy substituents in the hetaryl moiety, these hydrazones typically behave as *N,N*-bidentate ligands.

**Figure 7:**
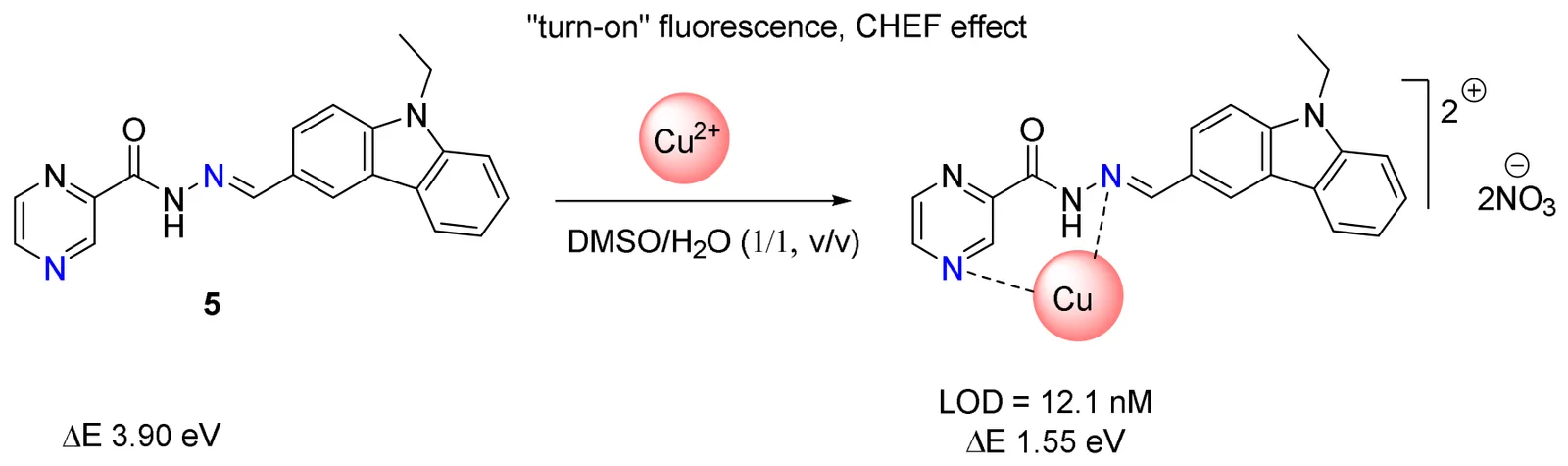
Molecular structure of hydrazone **5** based on pyrazine-2-carbohydrazide and *N*-ethylcarbazole-3-carbaldehyde and proposed interaction mode with Cu^2+^ cation upon addition of Cu(NO_3_)_2_. Color coding: nitrogen atoms involved in metal coordination are shown in blue.

Sun et al. [61] reported a pyrazine-based Schiff-base fluorescent probe **5** (Figure 7) for colorimetric and fluorimetric detection of Cu^2+^ in a DMSO/H_2_O (1:1, *v*/*v*). Probe **5** contains an N-ethylcarbazole fluorophore linked to a pyrazine–hydrazone receptor. In the free state, **5** exhibits very weak fluorescence, which was attributed to PET from the electron-rich receptor to the excited carbazole unit. Upon addition of Cu^2+^, the solution changed from colorless to bright yellow, and a strong emission band appeared at 500 nm. Among a broad panel of alkali, alkaline earth, transition and heavy metal ions, only Cu^2+^ induced a hypsochromically shifted, tail-shaped absorption band together with this intense luminescent response.

Coordination of Cu^2+^ suppresses the PET pathway and activates CHEF, as supported by DFT calculations showing HOMO localization at the Cu-binding site. The high Lewis acidity of Cu^2+^ lowers the energy of the donor orbital on the receptor, thereby inhibiting PET; DFT calculations at the B3LYP/6-31G(d) level support this interpretation, showing that the HOMO becomes localized at the Cu-binding site in the complex. The **5**-Cu^2+^ complex has a significantly narrower HOMO–LUMO gap (1.55 eV) than the free probe (3.90 eV), which is consistent with the observed spectral changes and suggests greater electronic stabilization upon coordination; however, this parameter alone does not establish thermodynamic stability, which would require equilibrium binding data [61]. Mechanistically, **5** operates through a PET→CHEF switching mechanism: in the free probe, PET from the electron-rich receptor to the excited carbazole unit dominates, whereas Cu^2+^ coordination lowers the energy of the donor orbital and suppresses this pathway, activating CHEF.

A 1:1 binding stoichiometry for the **5**-Cu^2+^ complex (Figure 7) was established by Job’s plot analysis, fluorescence titration, and UV–vis spectroscopy. According to ^1^H NMR data, probe **5** coordinates Cu^2+^ through the pyrazine nitrogen and the imine nitrogen of the C=N bond (N,N-coordination mode). The system **5** operates over a broad pH range (4–10), exhibits a low detection limit of 12.1 nM and shows excellent performance in colorimetric detection of Cu^2+^ in real water samples, highlighting its potential for environmental monitoring and analytical applications [61].

Introduction of an additional coordination site, such as a carbonyl or alkoxy oxygen, into the hydrazone framework (R–CH=N–NH–C(O)–Pyraz) changes the coordination mode: the hydrazone can act as an O,N,O-tridentate ligand, while the pyrazine moiety remains uninvolved in metal binding.

**Figure 8:**
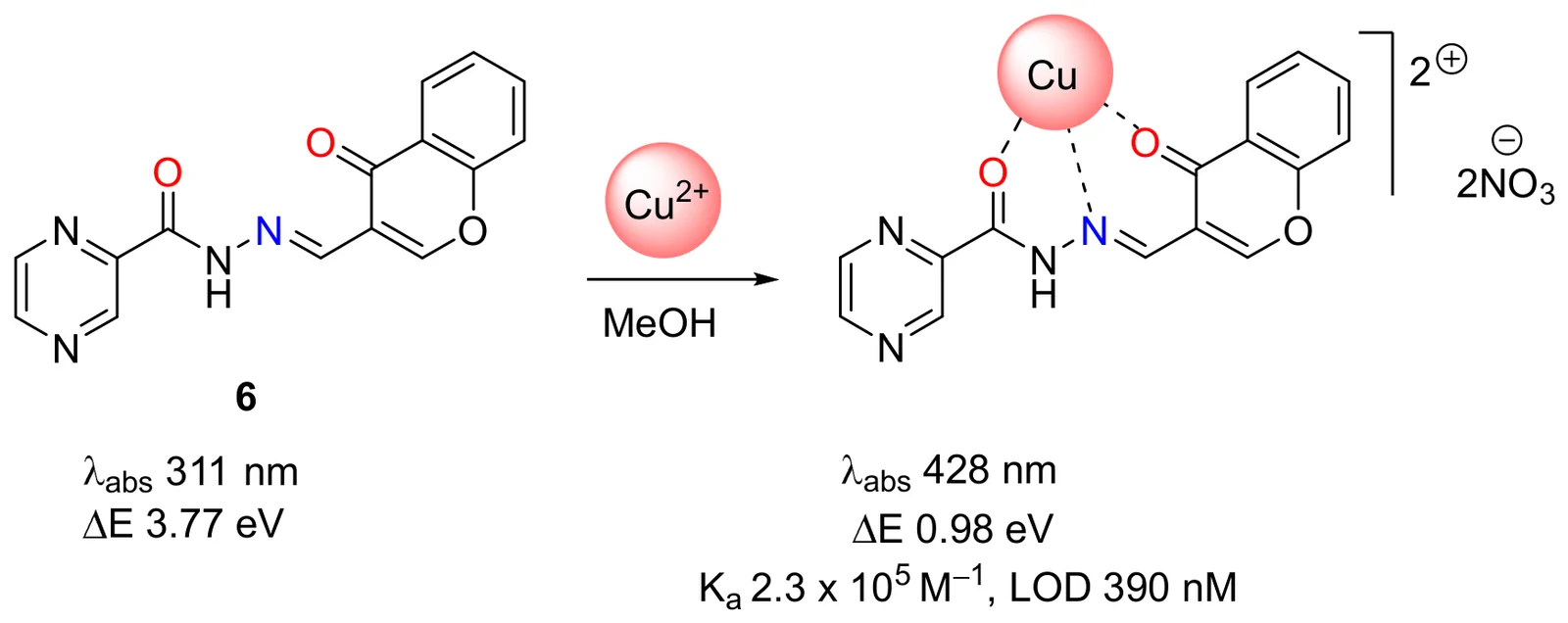
Molecular structure of chromone-based Schiff base **6** and the proposed interaction mode with Cu^2+^ upon addition of Cu(NO_3_)_2_ × H_2_O. Color coding: nitrogen atom involved in metal coordination is shown in blue; oxygen atoms involved in metal coordination are shown in red.

Tomer et al. [62] synthesized the chromone-based Schiff-base ligand **6** (Figure 8) by condensation of 3-formylchromone with pyrazine-2-carbohydrazide. Compound **6** exhibited an immediate (≈40 s) and selective colorimetric response to Cu^2+^ in methanol, with the solution changing from colorless to bright yellow; no interference was observed in the presence of a broad panel of competing metal ions (Na^+^, K^+^, Mg^2+^, Ca^2+^, Pb^2+^, Mn^2+^, Co^2+^, Ni^2+^, Ag^+^, Zn^2+^, Cd^2+^, Hg^2+^, Zr^2+^, Fe^3+^, Al^3+^ and Cr^3+^). The bathochromic shift from 311 to 428 nm is attributed to enhanced ICT upon O,N,O-tridentate complexation; no significant fluorescence response is observed, making **6** a purely colorimetric ICT-based sensor. Job’s plot and HRMS data (peak at *m*/*z* 355.9) confirmed a 1:1 binding stoichiometry for the **6**-Cu^2+^ complex. The association constant K_a_, determined using the Benesi–Hildebrand equation, was 2.3 × 10^5^ M^−1^, while the detection limit was 390 nM [62].

DFT calculations at the B3LYP level (6-311G(d,p) for **6**, LANL2DZ for Cu^2+^) revealed a substantial reduction in the HOMO–LUMO gap from 3.77 eV (free ligand) to 0.98 eV (complex), which is consistent with the observed bathochromic shift; however, the orbital energy difference should not be equated directly with the experimental absorption energy, and the proposed electron-transfer pathway remains suggestive rather than proven. The optimized geometry confirmed tridentate O,N,O-coordination of Cu^2+^, with Cu–O bond lengths of 1.9411 and 1.9763 Å and a Cu–N bond length of 2.1258 Å [62].

The sensor responded rapidly and operated effectively over a broad pH range (6–11), with optimal performance at pH 7.9. Its practical applicability was validated in real water samples (tap, groundwater, and canal water), with recoveries of 98–108% and relative standard deviation (RSD) values below 2.8% [62].

**Figure 9:**
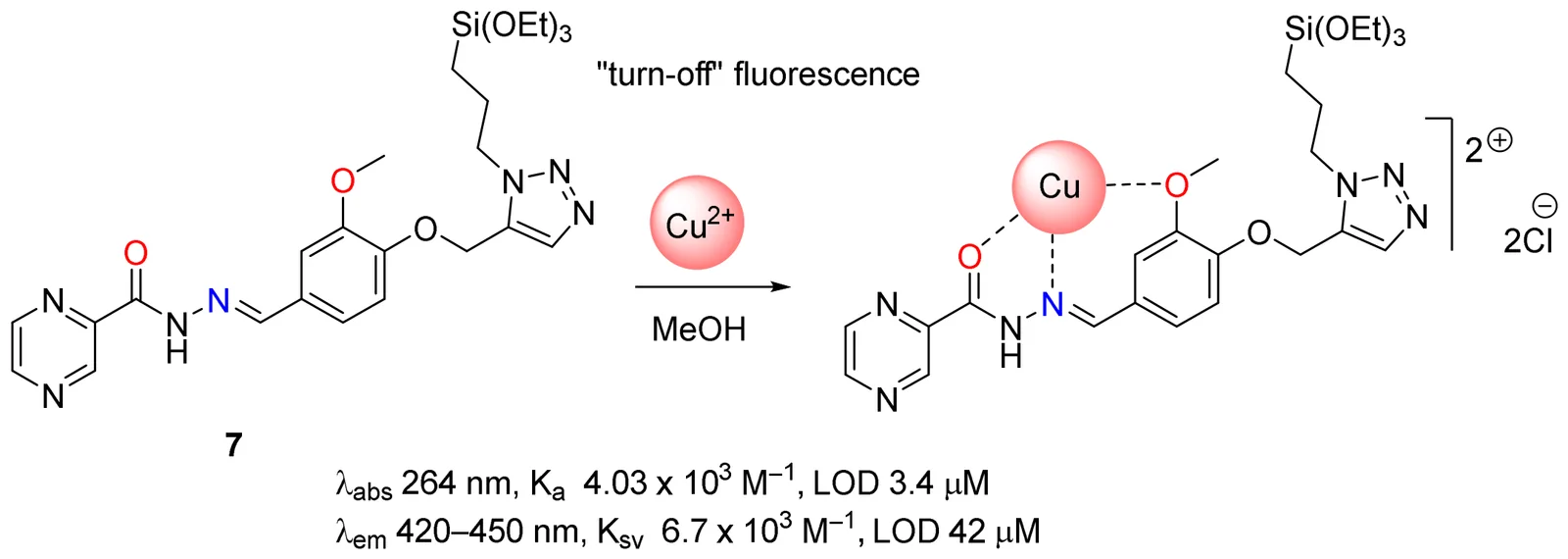
Pyrazine-based 1,2,3-triazole-linked organosilane **7** and proposed its binding mode with Cu^2+^ upon addition of CuCl_2_. Color coding: nitrogen atom involved in metal coordination is shown in blue; oxygen atoms involved in metal coordination are shown in red.

Another O,N,O-tridentate selective sensor for Cu^2+^ was designed by G. Sing et al. [63]. It is based on an 1,2,3-triazol-5-yl)methoxy)benzylidene)pyrazine-2-carbohydrazide **7** (Figure 9). Product **7** exhibited high selectivity toward Cu^2+^ over 14 other metal ions and responded in methanol with a hyperchromic shift in the UV–vis spectrum and pronounced fluorescence quenching. The “turn-off” response is ascribed to CHEQ operating through excitation energy transfer to the Cu^2+^ d-orbitals and the heavy-atom effect, rather than through simple static quenching.

A 1:1 stoichiometry for the **7**-Cu^2+^ complex was supported by Job’s continuous variation method and by the linearity of the Benesi–Hildebrand plot; reversibility was demonstrated by addition of EDTA. NMR analysis suggested coordination through the amide nitrogen atom and the methoxy oxygen substituent (Figure 9). The LODs were 3.4 µM (absorption) and 42 µM (fluorescence), with an association constant of 4.03 × 10^3^ M^−1^ and Stern–Volmer quenching constant of 6.7 × 10^3^ M^−1^. Notably, immobilization of probe **7** on Fe_3_O_4_@SiO_2_ magnetic nanoparticles lowered the detection limits to 2.7 µM and 0.86 µM for absorption and emission, respectively, corresponding to an almost 50-fold improvement in the fluorescence LOD [63].

**Figure 10:**
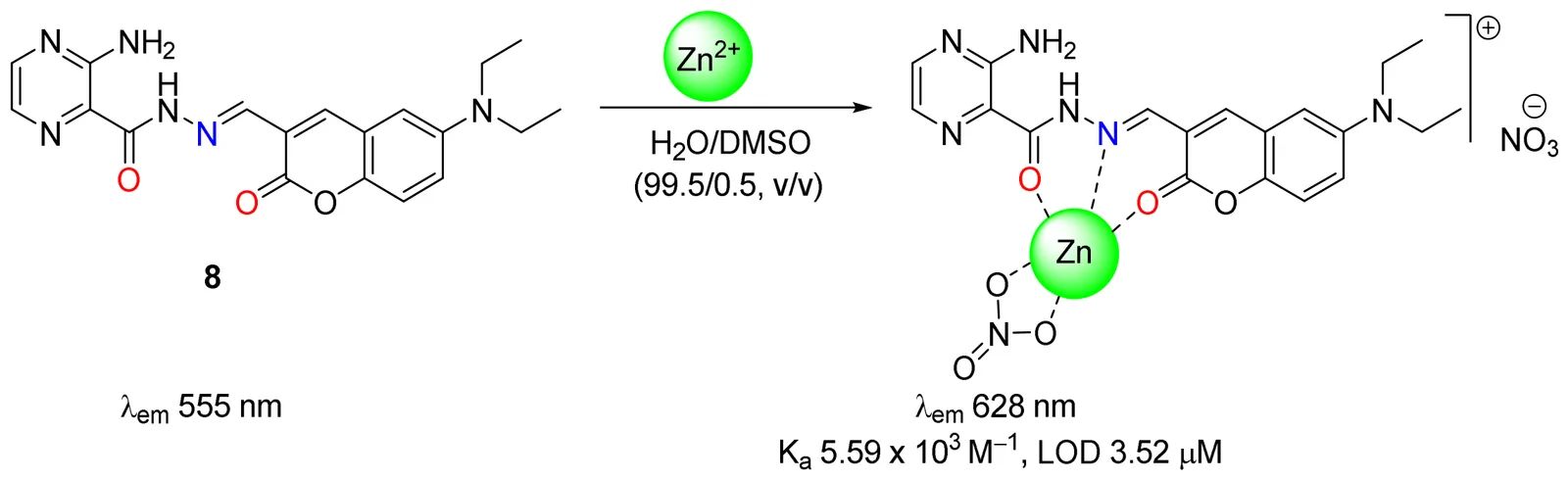
Chemosensor **8**, constructed from 3-amino-pyrazine-2-carbohydrazide and 7-diethylamino-3-formylcoumarin, and its binding mode with Zn^2+^ upon addition of Zn(NO_3_)_2_. Color coding: nitrogen atom involved in metal coordination is shown in blue; oxygen atoms involved in metal coordination are shown in red.

The condensation of 7-diethylamino-3-formylcoumarin with 3-amino-pyrazine-2-carbohydrazide provided an efficient route to the Schiff-base chemosensor **8** (Figure 10), capable of detecting Zn^2+^ ions in aqueous media [64]. Compound **8** exhibits a distinctive aggregation-induced ratiometric emission (AIRE) phenomenon in an aqueous buffer solution (99.5% H_2_O/DMSO); in this medium **8** displays orange emission with a maximum at 555 nm, whereas in pure DMSO it shows green fluorescence at 524 nm. Upon binding Zn^2+^, the aggregation-induced emission at 555 nm is quenched, while a new intense band appears in the far-red region at 628 nm (λ_ex_ = 470 nm), resulting in a large Stokes shift (158 nm) and a ratiometric response with the F_628_/F_555_ ratio increasing approximately 11-fold. Sensor **8** therefore enables selective Zn^2+^ detection in the presence of various competing metal ions, except Cu^2+^, which decreases the F_628_/F_555_ ratio of **8**-Zn^2+^ complex. The detection limit for Zn^2+^ was determined as 3.52 μM. Job’s plot analysis proposed a 1:1 binding stoichiometry, and the association constant obtained from the Benesi–Hildebrand equation was K_a_ = 5.59 × 10^3^ M^−1^. According to the ^1^H NMR and ESI-MS data, the **8**-Zn^2+^ complex formed under the studied condition corresponds to [Zn^2+^(**8**)(NO_3_)]^+^ (calcd *m*/*z* 506.08) and features an O,N,O-coordination mode (Figure 10). The experimental results are consistent with DFT calculations, which gave HOMO-LUMO gaps of 3.20 for **8** and 2.95 eV for the **8**-Zn^2+^ complex; the smaller gap for the Zn^2+^ complex suggests greater electronic stabilization upon coordination relative to the unchelated ligand; however, this parameter alone does not provide a measure of thermodynamic stability. Sensor **8** therefore operates through an AIRE mechanism; the in situ formed **8**–Zn^2+^ complex detects pyrophosphate via aggregate disassembly, extending the sensing functionality to anions.

Sensor **8** is considered as promising for biological applications owing to its far-red emission, ratiometric fluorescent output, and operation under physiological conditions. Notably, the in situ generated **8**–Zn^2+^ complex can be further employed for secondary recognition of pyrophosphate (P_2_O_7_^4−^, PPi) via an aggregate disassembly mechanism (LOD = 2.45 μM), thereby expanding the sensor’s functionality and highlighting its potential for bioimaging in HeLa cells.

**Figure 11:**
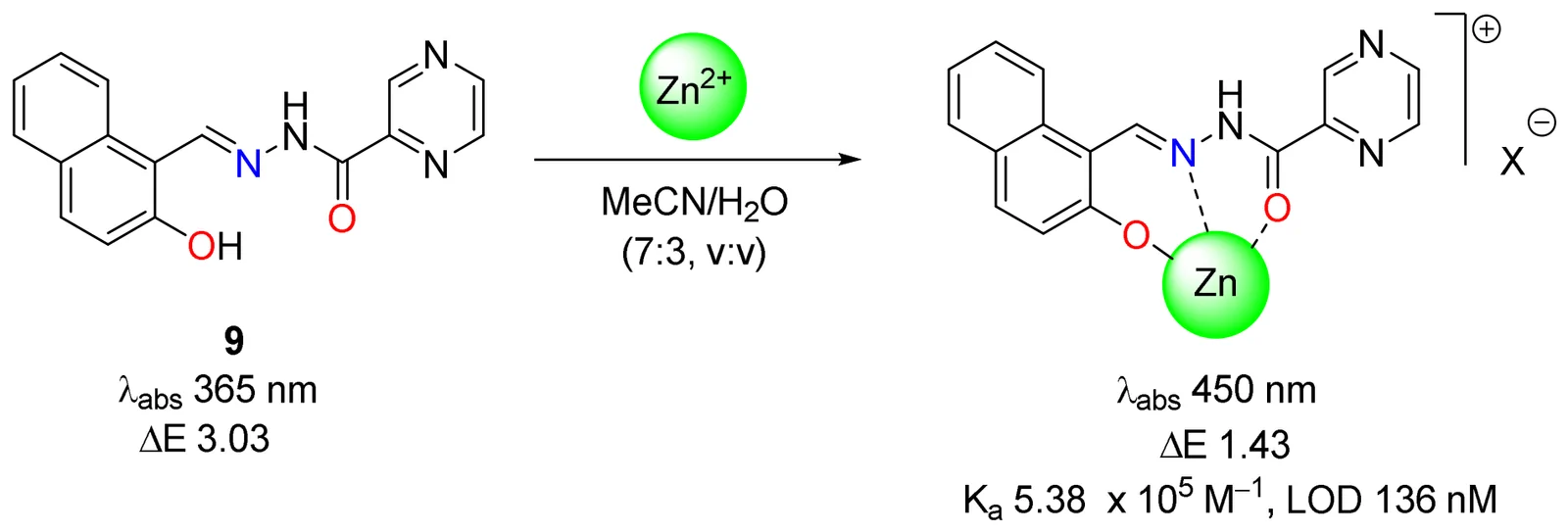
Chemosensor **9** and proposed interaction mode of **9** with Zn^2+^. The nature of anion X^−^ is not specified. Color coding: nitrogen atom involved in metal coordination is shown in blue; oxygen atoms involved in metal coordination are shown in red.

Dhanasekaran et al. [65] reported a pyrazine-carbohydrazide-based sensor **9** (Figure 11), synthesized by condensation of pyrazine-2-carbohydrazide with 2-hydroxy-1-naphthaldehyde, for Zn^2+^ detection by colorimetric and ratiometric methods in a partially aqueous medium (CH_3_CN:H_2_O, 7:3, *v*/*v*). The free ligand **9** exhibits an absorption band at 365 nm; upon addition of Zn^2+^ a bathochromic shift of 85 nm occurs, with the emergence of a new intense band at 450 nm and a visible color change from colorless to yellow [65]. The presence of an isosbestic point at 393 nm confirms formation of a new complex, while the ratiometric response ensures high selectivity for Zn^2+^ in the presence of a wide range of competing metal ions (Cu^2+^, Cd^2+^, Co^2+^, Pb^2+^, Ni^2+^, Mg^2+^, Mn^2+^, Cr^3+^, Fe^3+^, Al^3+^, and others). The ratiometric response arises from CHEF activated by deprotonation of the phenolic OH group upon Zn^2+^ coordination, as confirmed by ^1^H NMR titration.

The binding mechanism was elucidated by ^1^H NMR titration, IR spectroscopy, and HRMS, indicating coordination via the deprotonated hydroxyl group, the carbonyl oxygen, and the imine nitrogen, with formation of a 1:1 stoichiometric complex (Figure 11). This assignment was further supported by Job’s plot and DFT calculations. The limit of detection for sensor **9**, calculated from UV–Vis spectroscopy data, is 136 nM, demonstrating high sensitivity, while the high association constant (K_a_ = 5.38 × 10^5^ M^−1^, determined by the Benesi–Hildebrand method) indicates strong binding. Sensor **9** was successfully applied to determine Zn^2+^ in pharmaceutical samples (Zincovit tablets and syrup), in real water samples, and as test strips showing a color transition, confirming its potential for on-site analysis in various matrices.

**Scheme 3:**
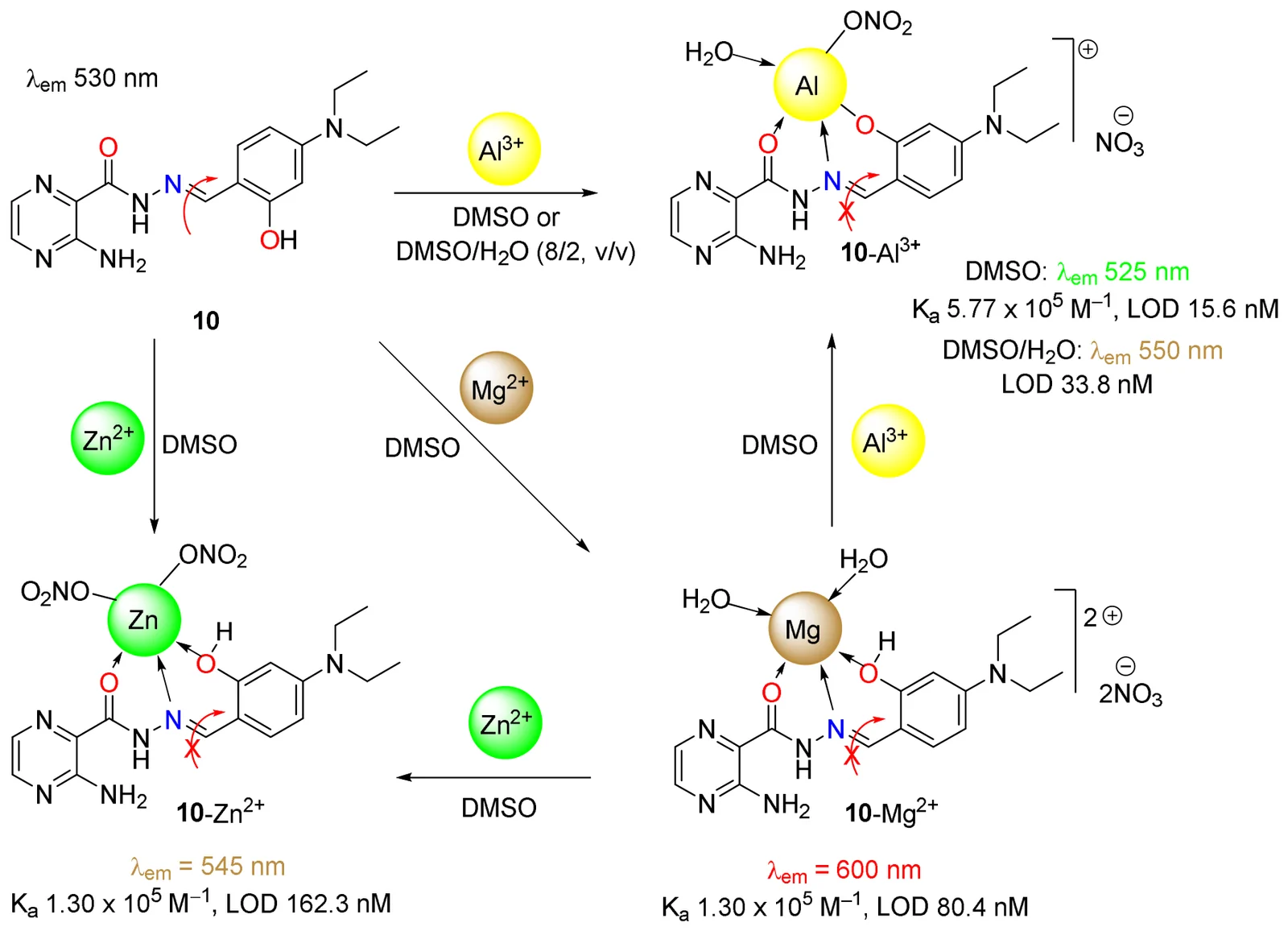
Schematic illustration of the binding interactions of pyrazine-based sensor **10** with Al^3+^, Mg^2+^, and Zn^2+^ ions upon addition of their nitrate salts. Color coding: nitrogen atoms involved in metal coordination are shown in blue; oxygen atoms involved in metal coordination are shown in red; the arrow indicated rotation of the molecular fragments shown in red.

Among the tridentate O,N,O chelators, salicylidene hydrazones are extensively used in chemosensing. A developed pyrazine-based acylhydrazone sensor **10** [66] acts as a multi-analyte fluorescent “turn-on” probe for Al^3+^, Mg^2+^, and Zn^2+^ ions in DMSO solution, displaying three distinct emission colors (green, red and yellow, respectively) and LODs of 15.6, 80.4 and 162.3 nM (Scheme 3). The sensing mechanism relies on inhibition of ESIPT upon metal coordination through the phenolic hydroxyl oxygen, carbonyl oxygen, and imine nitrogen (Scheme 3), which results in significant fluorescence enhancement. Notably, the **10-**Al^3+^ complex differ from **10**-Zn^2+^ and **10**-Mg^2+^ species, as evidenced by ESI-MS, FT-IR studies and ^1^H NMR studies. Binding of **10** to Al^3+^ is accompanied by deprotonation of the phenolic OH group, whereas the bivalent metals Zn^2+^ and Mg^2+^ coordinate to ligand **10** via donor–acceptor interaction without deprotonation (Scheme 3). The interaction between **10** and Al^3+^, Mg^2+^, and Zn^2+^, was completed within 120, 5, and 20 min, respectively, indicating that the reaction with Mg^2+^ is the most dynamically favorable among the three metal ions; the considerably longer interaction time for Al^3+^ may be attributed to covalent bond formation involving deprotonation.

The calculated binding constants for ligand **10** with Al^3+^, Zn^2+^ and Mg^2+^ are 5.77, 3.87 and 1.30 × 10^5^ M^−1^, respectively, reflecting the higher affinity of sensor **10** for Al^3+^ and Zn^2+^ over Mg^2+^. Moreover, the HOMO–LUMO energy gaps of the complexes (3.25, 3.46, and 3.41 eV for **10**-Al^3+^, **10**-Mg^2+^, and **10**-Zn^2+^, respectively) are lower than that of the free ligand (3.48 eV), suggesting increasing electronic stabilization in the order: **10** < **10**-Mg^2+^ < **10**-Zn ^2+^ < **10**-Al^3+^; this ordering should be regarded as indicative rather than definitive, since thermodynamic stability requires independent binding data. On this basis, the authors propose the **10**-Mg^2+^ system as a ratiometric sensor for Al^3+^ and Zn^2+^ operating via a metal-displacement mechanism (Scheme 3). Mechanistically, **10** operates through ESIPT inhibition combined with CHEF; for Al^3+^, ESIPT inhibition is accompanied by deprotonation of the phenolic OH, whereas Zn^2+^ and Mg^2+^ coordinate without deprotonation, leading to distinct emission colors (green, yellow, and red, respectively).

Additionally, in DMSO/H_2_O solution, sensor **10** selectively recognizes Al^3+^ with a “turn-on” response and a detection limit of 33.8 nM, which is approximately two-fold higher than that in pure DMSO (LOD = 15.6 nM). Furthermore, the in situ generated **10-**Al^3+^ complex serves as a “turn-off” sensor for fluoride anions (F^−^) due to the formation of a stable Al^3+^/F^−^ adduct, which regenerates the free sensor **10**. Consequently, compound **10** enables sequential detection of metal cations and anions, with demonstrated applicability for bioimaging in living HeLa cells [66].

**Scheme 4:**
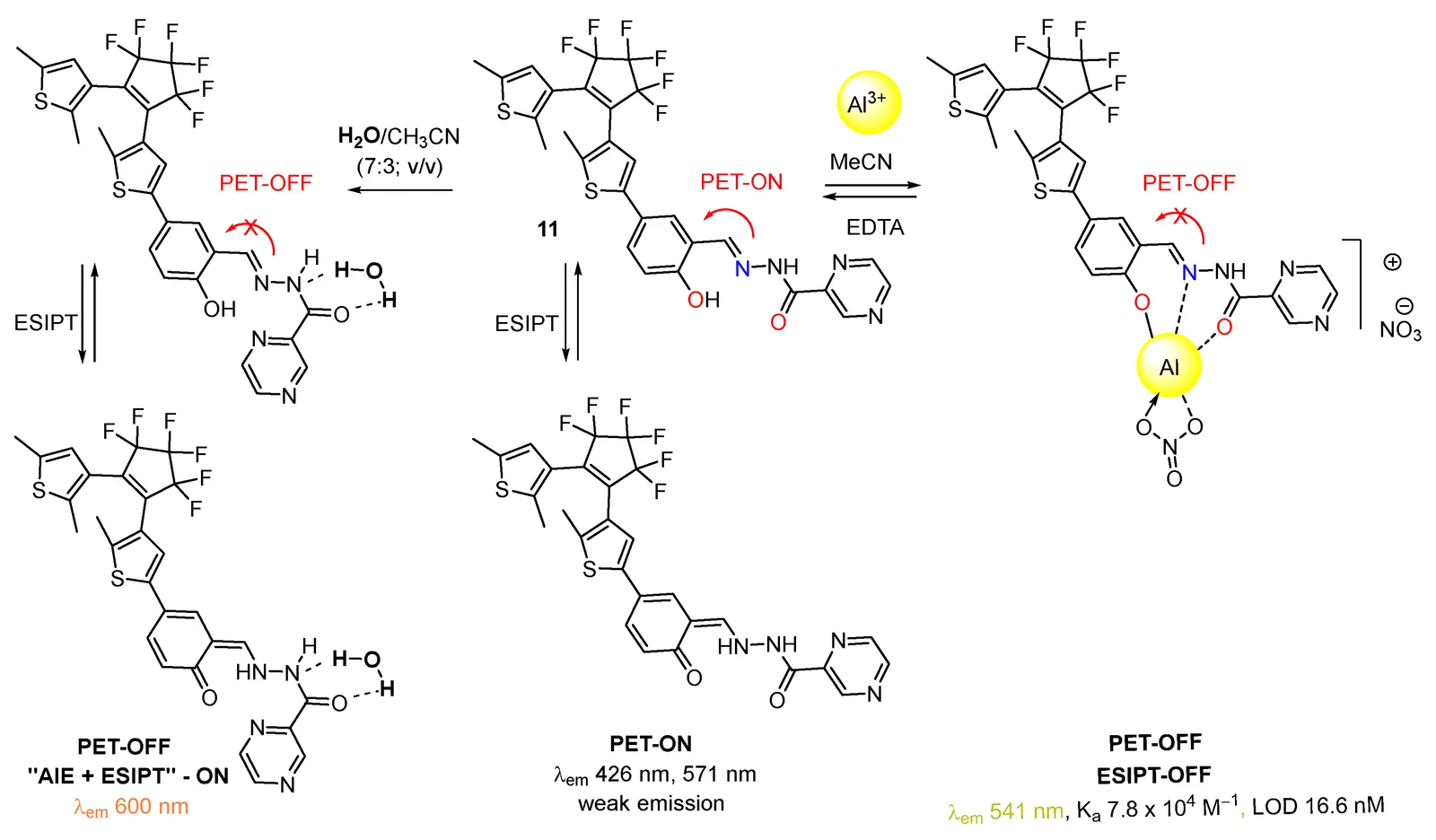
Molecular structure of sensor **11** and the plausible mechanism for fluorescent turn-on upon addition of H_2_O or Al(NO_3_)_3_. Color coding: nitrogen atom involved in metal coordination is shown in blue; oxygen atoms involved in metal coordination are shown in red; the proposed detection mechanism is shown in red.

The selective sensor **11** (Scheme 4), designed for Al^3+^ recognition and suitable for quantitative determination of this cation in real water samples, is based on a pyrazine-2-carbohydrazide condensation product with a salicylaldehyde-containing photochromic diarylethene derivative [67]. Sensor **11** exhibits an LOD of 16.6 nM and K_a_ of 7.8 × 10^4^ M^−1^. As in the case of sensor **10**, ligand **11** binds Al^3+^ through the deprotonated phenolic hydroxyl group, the imine (CH=N) nitrogen, and the carboxyl (C=O) oxygen in a 1:1 stoichiometry (Scheme 4). This binding mode is supported by an ESI-MS peak at *m*/*z* 710.40, assigned to [**11**+Al^3+^+NO_3_−H]^+^. The authors discuss in detail the mechanisms underlying the photophysical properties of **11**. The free ligand shows weak fluorescence due to a PET process, with two emission maxima at around 420 and 570 nm, which are attributed to tautomerization associated with ESIPT. Increasing the water content (>70 vol%) in the organic solvent induces AIE, accompanied by enhancement of the keto-isomer emission at 600 nm. In the presence of water, strong solvation of the polar N–H group weakens the PET process. The observed fluorescence “turn-on” thus arises from the synergistic action of intramolecular hydrogen bonding and solvation. Upon coordination of ligand **11** with Al^3+^, both PET and ESIPT processes are suppressed; concomitantly, intramolecular rotations are restricted and the molecular structure is rigidified, leading to a pronounced increase in emission intensity centered at 541 nm, accompanied by a blue shift of 30 nm and distinct yellow fluorescence, and underlies the high selectivity of **11** for Al^3+^ over a wide range of competing metal ions.

**Figure 12:**
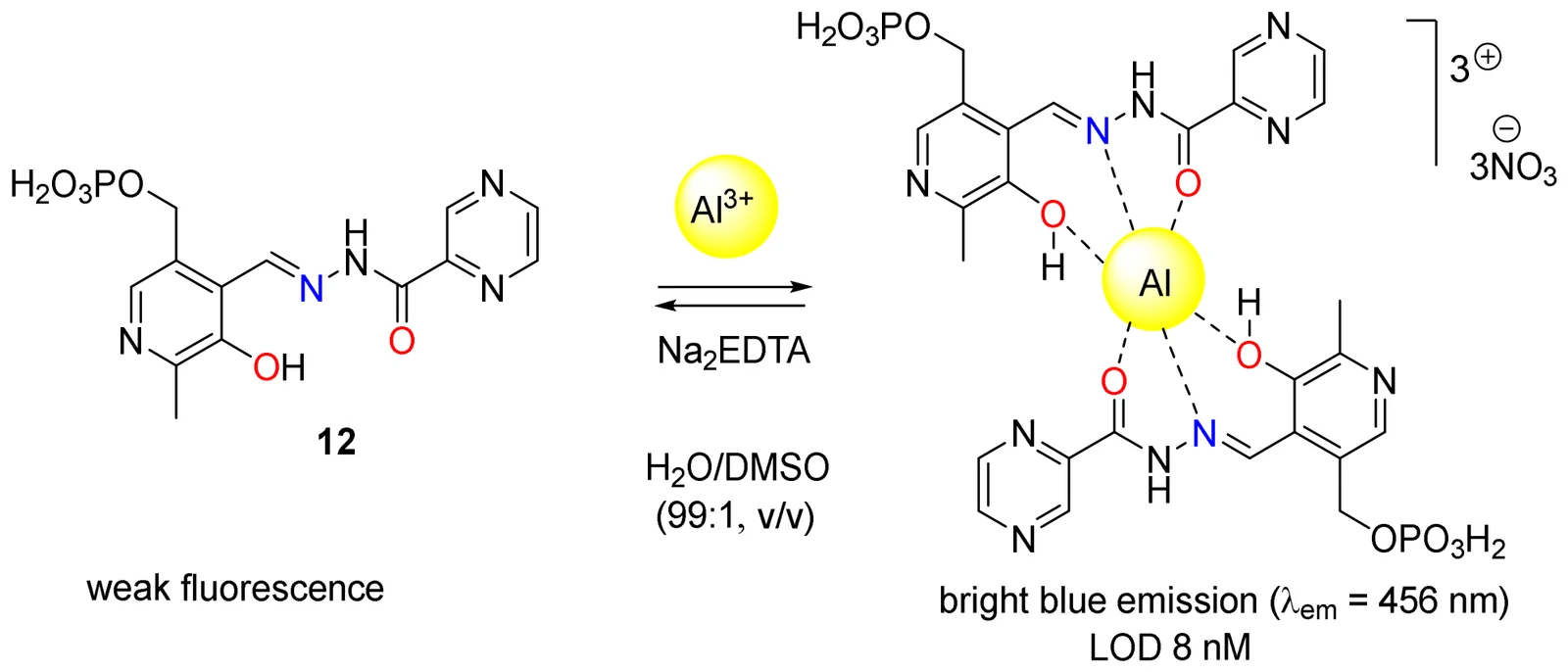
Vitamin B_6_-based fluorescent sensor **12** and its proposed interaction with Al^3+^ upon subsequent addition of Al(NO_3_)_3_. Color coding: nitrogen atom involved in metal coordination is shown in blue; oxygen atoms involved in metal coordination are shown in red.

Ligand **12** (Figure 12)**,** derived from pyridoxal-5′-phosphate (a vitamin B_6_ derivative) and pyrazine-2-carbohydrazide, binds Al^3+^ in 1 vol% DMSO solution, producing a selective “turn-on” fluorescence response at 456 nm (λ_ex_ = 410 nm) with a 20-fold enhancement of blue emission [68]. The recognition mechanism is based on restriction of rotation around the hydrazide bridge upon formation of a coordination compound. Authors showed that Na^+^, K^+^, Ca^2+^, Mg^2+^, Ba^2+^, Cd^2+^, Pb^2+^ and Cr^3+^ ions have almost no effect on fluorescent Al^3+^ recognition in water, whereas Cu^2+^ interferes with both qualitative and quantitative determination; in the presence Ni^2+^, Hg^2+^, UO_2_^2+^, Co^2+^ and Fe^3+^ ions, only qualitative determination of Al^3+^ is possible. The effect of a set of anions on the fluorescence of the **12**-Al^3+^ complex is negligible. In contrast, UV–vis spectroscopy is not suitable for selective recognition of metal ions, because interaction of probe **12** with several metals (Co^2+^, Ni^2+^, Cu^2+^, Hg^2+^, UO_2_^2+^, Al^3+^ and Ce^3+^) induces similar changes (from colorless to yellow) and comparable bathochromic shifts. The LOD for Al^3+^ is 8 nM (by the 3σ method), which is three orders of magnitude below the World Health Organization (WHO) guideline for drinking water (7.4 μM); the LOQ is 27 nM. The sensor exhibits reversibility upon addition of Na_2_EDTA and was successfully applied to the quantitative determination of Al^3+^ in real water samples (distilled, river, and mineral water) with high recovery rates of 86–99%.

A distinct Al^3+^ recognition mode (Figure 12) was proposed for chemosensor **12**, in contrast to the related compounds **10** and **11**. A 2:1 (**12**:Al^3+^) stoichiometry for the **12**-Al^3+^ complex was established from spectrofluorimetric titration data and mass spectrometry (peak at *m*/*z* 760.99). The binding mechanism was elucidated using ^1^H NMR titration, IR spectroscopy, and DFT calculations, which indicated coordination of Al^3+^ through the phenolic oxygen (without deprotonation), azomethine nitrogen, and carbonyl oxygen, forming an O,N,O chelate cavity. It was also established that the phosphate group does not participate in binding (confirmed by ^31^P NMR) [68].

A notable feature of probe **12** is the manifestation of AIE: both the free sensor and its Al^3+^ complex form nanoaggregates in aqueous media (sizes of ~1000 nm for **12** and ~3 nm for the **12**-Al^3+^ complex), which further enhances the fluorescence response. The 20-fold emission enhancement is attributed to CHEF assisted by AIE, with the formation of nanoaggregates (~3 nm) further amplifying the response.

**Figure 13:**
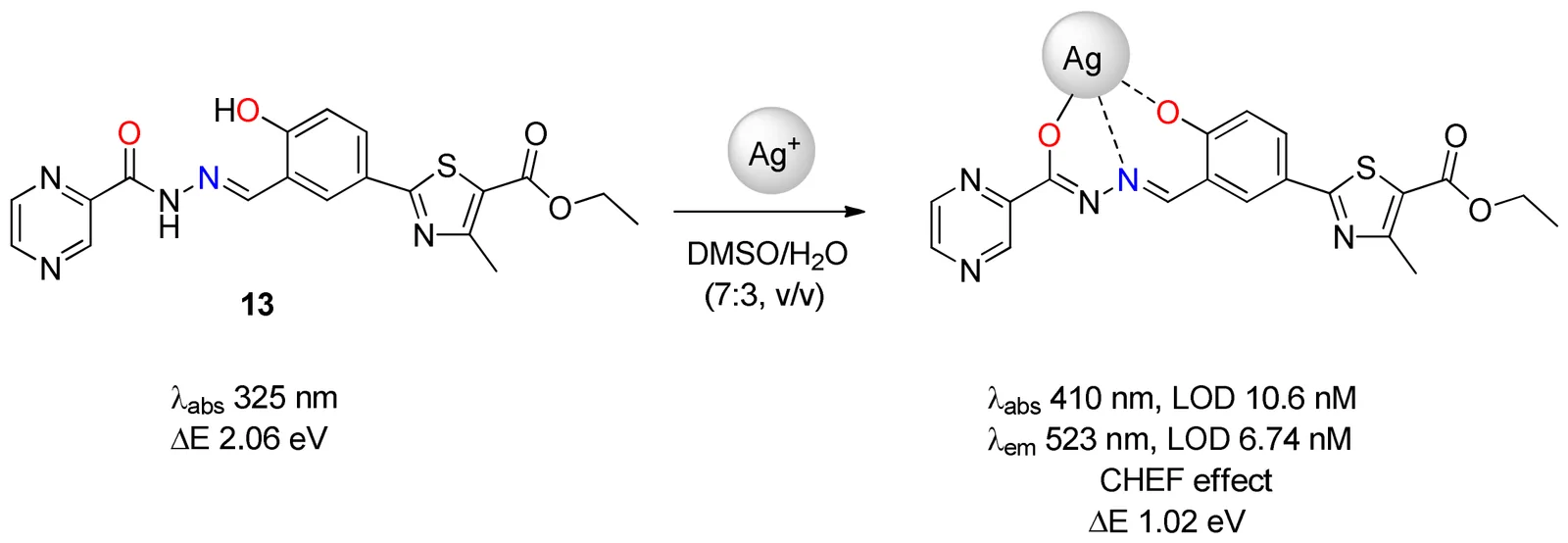
Thiazole-derived pyrazin-2-carbohydrazide chemosensor **13** and its proposed interaction with Ag^+^ cation upon addition of AgNO_3_. Color coding: nitrogen atom involved in metal coordination is shown in blue; oxygen atoms involved in metal coordination are shown in red.

In contrast to the Al^3+^-selective salicylidene hydrazones **10**–**12**, the thiazole-substituted chemosensor **13** (Figure 13) functions as a highly selective dual-channel probe for Ag^+^, operating through both colorimetric and fluorescent “turn-on” responses in a DMSO/H_2_O (7:3, *v*/*v*) medium [69]. The sensing mechanism relies on the coordination of Ag^+^ with the hydroxyl fragment, imine nitrogen and carbonyl oxygen of the Schiff base moiety (Figure 13), which effectively restricts the C=N isomerization and enhances the CHEF effect, as supported by ^1^H NMR, HRMS, and DFT studies. This complexation induces a distinct visual color change from pale yellow to blue under ambient light, accompanied by pronounced emission enhancement at 523 nm (λ_ex_ = 410 nm) under UV irradiation, with a 1:1 binding stoichiometry. Coordination of Ag^+^ restricts C=N isomerization and activates CHEF, producing a dual-channel colorimetric/fluorescent response with a distinct visual color change.

Sensor **13** exhibits remarkable sensitivity, with detection limits of 10.6 nM (absorption) and 6.74 nM (fluorescence), as determined by UV-vis and fluorescence titrations, respectively—values well below the WHO permissible limit for silver in drinking water (30 µM), demonstrating its suitability for potable water quality monitoring. Furthermore, **13** shows excellent selectivity over a wide range of competing cations, a rapid response time (<5 min), and reversible binding upon EDTA addition, making it operationally robust. Its practical applicability is convincingly validated through quantitative recovery experiments in real water samples (tap and lake water, 95–102% recovery), convenient paper-strip tests for on-site visual detection, and successful fluorescence imaging of intracellular Ag^+^ in MCF-7 breast cancer cells with negligible cytotoxicity (cell viability 75–91%), collectively positioning **13** as a versatile and promising chemosensor for environmental monitoring, point-of-use testing, and bioimaging applications [69].

**Scheme 5:**
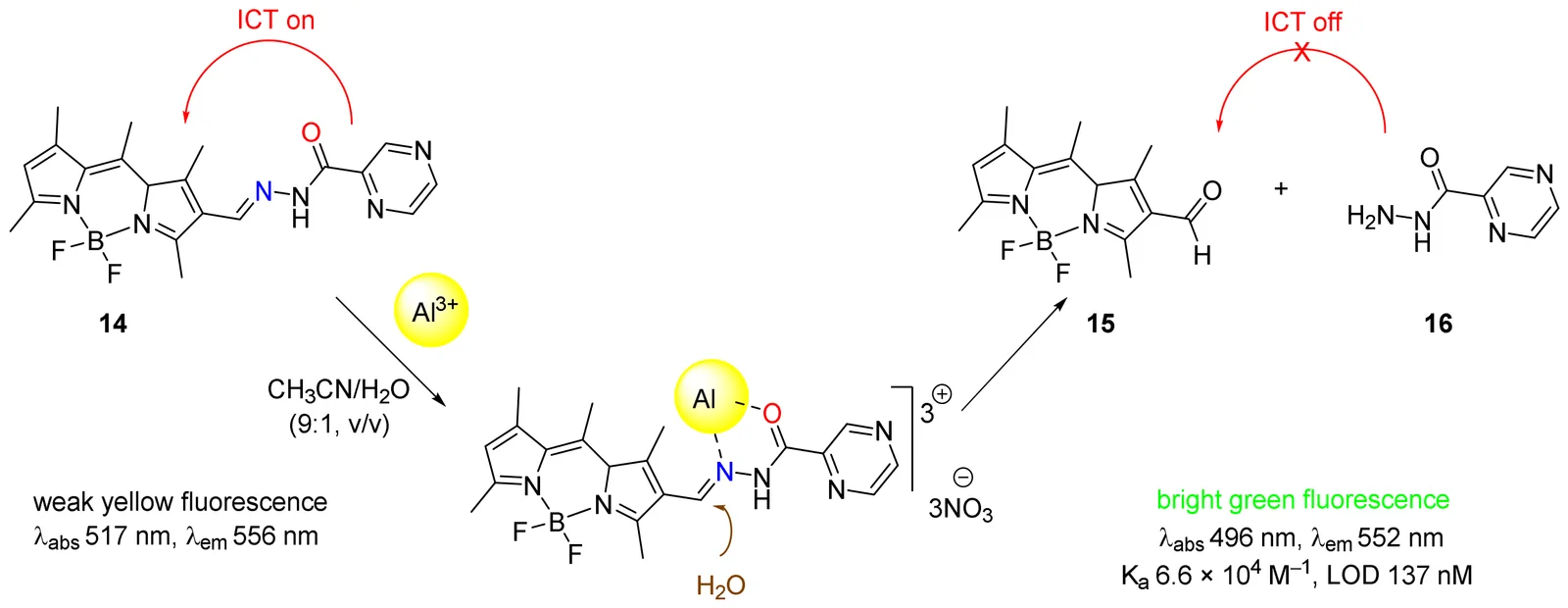
Molecular structure of BODIPY pyrazine-attached Schiff base chemosensor **14** and proposed mechanism for Al^3+^ sensing upon addition of Al(NO_3_)_3_ (>1.0 equiv.). Color coding: nitrogen atom involved in metal coordination is shown in blue; oxygen atom involved in metal coordination is shown in red; the proposed detection mechanism is shown in red.

A distinct mechanism for colorimetric and fluorescent detection of Al^3+^ in CH_3_CN/H_2_O (9:1, *v*/*v*) was reported for the BODIPY–pyrazine Schiff base chemosensor **14** (Scheme 5). Among a range of cations (Cs^+^, Ba^2+^, Al^3+^, V^3+^, Cr^3+^, Mn^2+^, Fe^3+^, Co^2+^, Ni^2+^, Cu^2+^ and Zn^2+^), only a large excess of Al^3+^ appreciably alters both the absorption and emission spectra, changing the solution color from pink to yellow under daylight and from pale orange to bright green under UV irradiation. In contrast to the coordination-based sensors discussed above, **14** operates as a chemodosimeter: excess Al^3+^ promotes hydrolysis of the imine bond (Scheme 5), converting the weakly fluorescent Schiff base into the green-emissive BODIPY-carbaldehyde **15**, with the pyrazine ring nitrogen atoms proposed to enhance nucleophilic attack on the imine system. Thus, a crucial role of the pyrazine core—specifically its ring nitrogen atoms—in governing the photophysical changes is suggested, in contrast to analogous benzene or pyridine rings [70]. The LOD of **14** for Al^3+^, calculated from fluorescence titration data, is 137 nM.

In addition, bioimaging studies demonstrated that chemosensor **14** enables selective identification of Al^3+^ ions in living cells, exhibiting a turn-on response both in vitro and in live-cell experiments; due to its cytocompatibility, it can be employed as a fluorescent marker [70].

In summary, pyrazine carboxamides and hydrazides represent the most extensively studied class of pyrazine-based cation sensors. Their popularity stems from the straightforward synthesis via amidation or Schiff-base condensation and the ability to form stable N,N,O-tridentate chelate complexes with a variety of metal ions, including Zn^2+^, Cu^2+^, Fe^3+^, Al^3+^, and Ag^+^. The sensing performance is highly tunable: variation in the aryl/hetaryl substituent on the amide or hydrazide moiety allows modulation of the binding affinity (K_a_ spanning from 10^3^ to 10^7^ M^−1^), detection limits (from nanomolar to micromolar), and selectivity patterns. Notably, while many of these sensors operate through a CHEF mechanism, the presence of paramagnetic ions such as Cu^2+^ and Fe^3+^ typically induces fluorescence quenching via CHEQ, offering complementary detection modes. The successful validation in real water samples, pharmaceutical formulations, and paper-strip tests highlights their practical potential, though mutual interference between competing cations, particularly Cu^2+^ and Fe^3+^, remains a challenge that requires careful molecular design to overcome.

### 2.2. 2-Acetylpyrazine Derivatives

2-Acetylpyrazine derivatives also belong to the family of pyrazine-based hydrazone Schiff-base ligands and have been successfully employed as “turn-on” fluorescent sensors for metal ions, in particular for Al^3+^ or Co^2+^, as reported in the sources cited below. A typical synthetic route includes condensation of the corresponding hydrazide with 2-acetylpyrazine in EtOH under reflux.

2-Acetylpyrazine derivatives typically act as N,N,O-tridentate ligands, in which the carbonyl oxygen, the imine nitrogen of the hydrazone bridge, and the pyrazine ring nitrogen cooperate in metal binding. The sensing response of this subclass is governed primarily by CHEF coupled with inhibition of C=N isomerization: coordination rigidifies the molecular framework, suppresses PET from the Schiff-base nitrogen to the fluorophore, and restricts non-radiative decay, resulting in pronounced “turn-on” fluorescence towards Al^3+^ and Co^2+^; in most cases, the proposed mechanisms rest on combined spectroscopic and computational evidence rather than on direct structural or thermodynamic data. The incorporation of rigid, highly emissive fluorophores such as pyrene or rhodamine 6G further enhances the photophysical performance and enables bioimaging applications.

**Figure 14:**
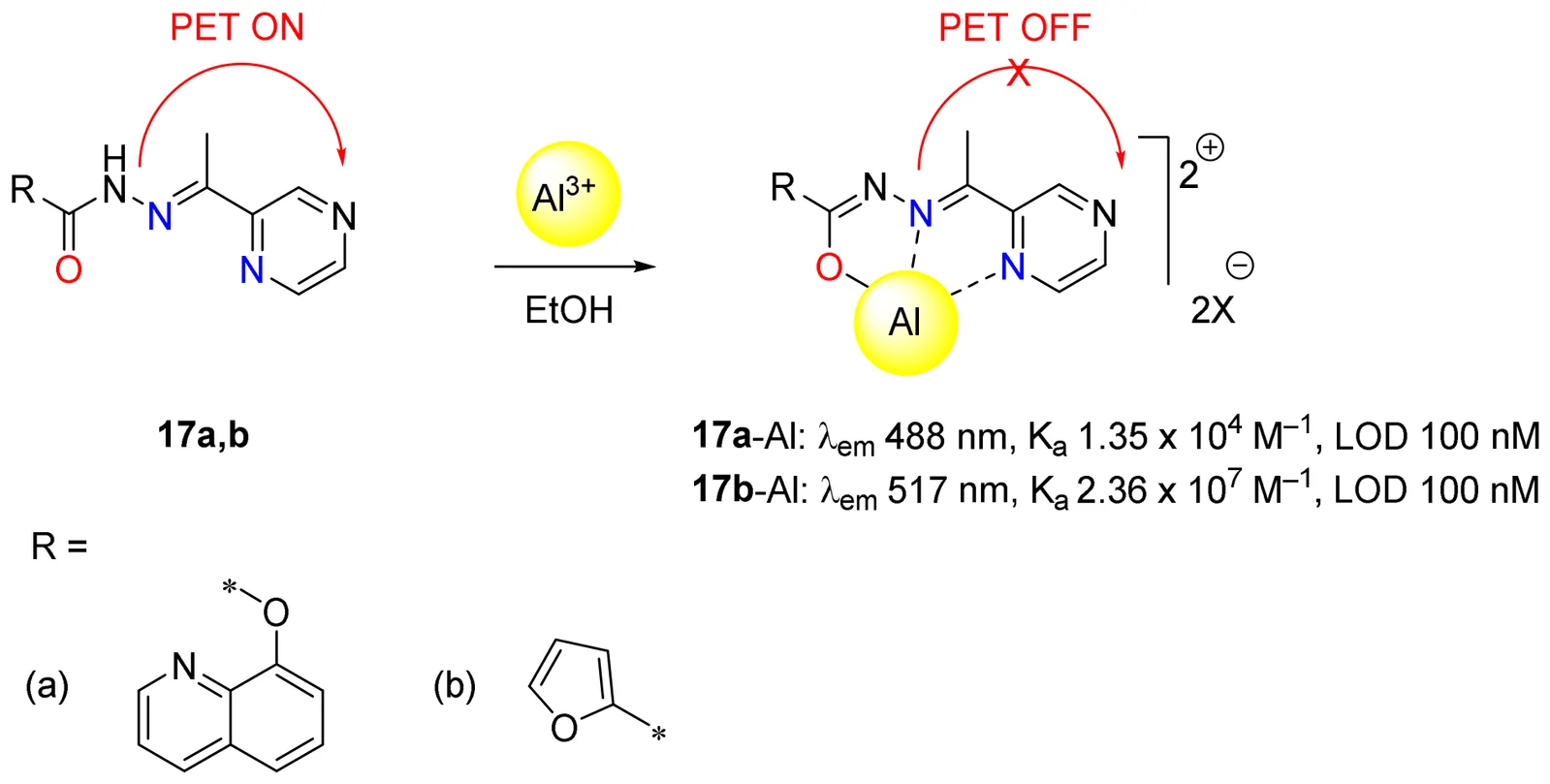
Proposed binding mode of (2-acetylpyrazine-(8′-hydroxyquinolineyl-2′-acetyl)hydrazones **17a** and 2-acetylpyrazine(2′-furanformyl)hydrazine **17b** with Al^3+^. The nature of anion X^−^ is not indicated. Color coding: nitrogen atoms involved in metal coordination are shown in blue; oxygen atom involved in metal coordination is shown in red; the proposed detection mechanism is shown in red.

**Figure 15:**
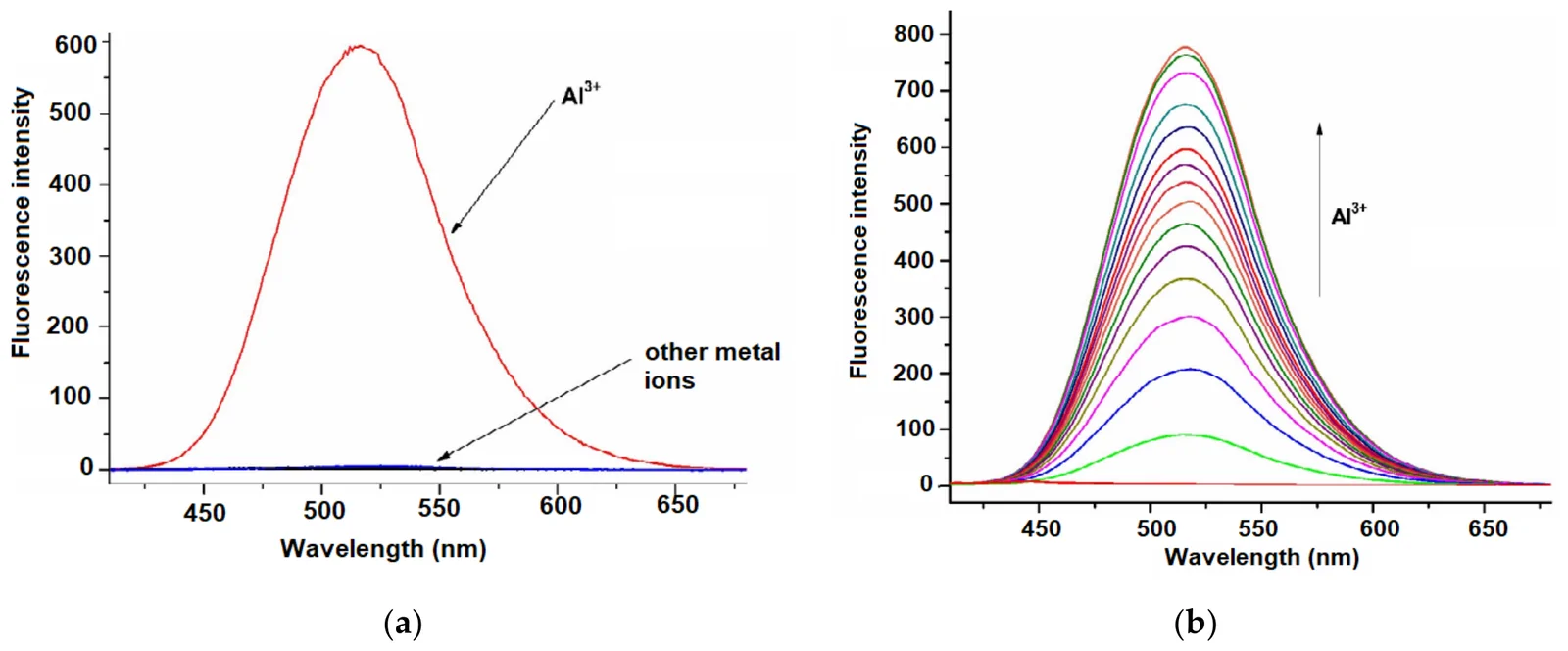
(**a**) Fluorescence spectra of **17b** (50 µM) upon addition of Al^3+^ (1 equiv.) and other metal ions (5 equiv.) in ethanol with an excitation at 382 nm; (**b**) Fluorescence spectra of **17b** (50 µM) upon the titration of Al^3+^ (from bottom to top: 0, 0.1, 0.2, 0.3, 0.4, 0.5, 0.6, 0.7, 0.8, 0.9, 1.0, 1.2, 1.4, 1.6, 1.8, 2.0 equiv., respectively) in ethanol with an excitation at 382 nm. Reproduced with permission of Royal Society of Chemistry [72].

Li et al. developed simple pyrazine-substituted sensors **17a** and **17b**, in which the hydrazone fragment incorporates quinolone (**17a**) or furan (**17b**) moieties (Figure 14) [71,72]. Both compounds function as highly selective and sensitive “turn-on” fluorescent chemosensors for Al^3+^ in ethanol (Figure 15). In their free state, sensors **17a,b** exhibit negligible fluorescence due to a PET process from the Schiff-base nitrogen atom to the pyrazine fragment. Upon coordination with Al^3+^, the metal ion binds to the carbonyl oxygen, the imine nitrogen, and one of the pyrazine nitrogen atoms in a 1:1 stoichiometry, as confirmed by Job’s plot analysis, ^1^H NMR titrations, and mass spectrometry. Both sensors are proposed to operate through CHEF coupled with inhibition of C=N isomerization, yielding 230-fold (**17a**) and 164-fold (**17b**) emission enhancements [71,72].

The selectivity of sensors **17a,b** for Al^3+^ was evaluated in the presence of 15 competing metal ions (Ba^2+^, Ca^2+^, Cd^2+^, Co^2+^, Cr^3+^, Cu^2+^, Fe^2+^, Fe^3+^, K^+^, Mg^2+^, Mn^2+^, Na^+^, Ni^2+^, Pb^2+^, Zn^2+^). Among them Cu^2+^ and Ni^2+^ (for **17a**) and Cu^2+^ and Fe^2+^ (for **17a**) completely quenched the fluorescence, whereas other metals caused slight quenching or did not interfere with Al^3+^ detection. Both sensors exhibit LOD on the order of 100 nM, but differ markedly in their binding constants: K_a_ = 1.35 × 10^4^ M^−1^ for **17a** and K_a_ = 2.36 × 10^7^ M^−1^ for **17b**.

The sensing systems **17a,b** are reversible: addition of Na_2_EDTA dissociates the **17**-Al^3+^ complex and quenches the fluorescence, while re-addition of Al^3+^ restores the emission signal, confirming their potential for practical applications.

**Figure 16:**
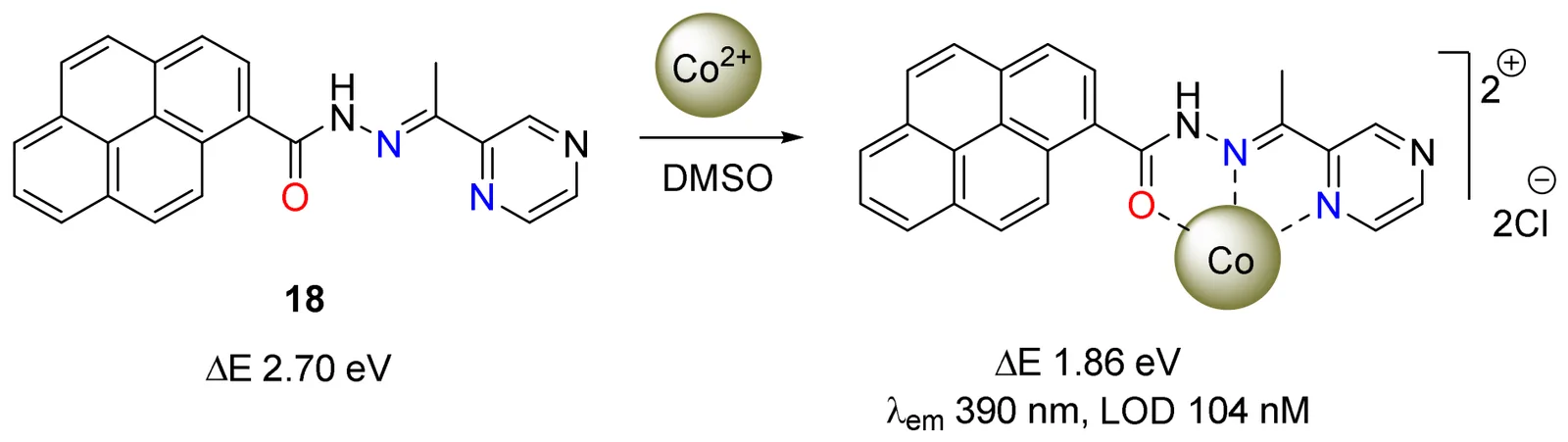
Pyrene-based fluorescent sensor **18** and its proposed mechanism for Co^2+^ sensing upon addition of CoCl_2_. Color coding: nitrogen atoms involved in metal coordination are shown in blue; oxygen atom involved in metal coordination is shown in red.

Later, a pyrazine-based fluorescent probe **18** (Figure 16) containing a pyrene fragment were designed [73]. The combination of a rigid, highly conjugated pyrene unit with a C(O)NHNC(CH_3_)-pyrazine receptor group afforded a highly effective probe for Co^2+^ ions. Its efficiency and selectivity are evidenced by an ultralow LOD value of 0.104 nM (6.15 ppb), which is well below the national safety standard for cobalt in drinking water (1.0 ppm, 16.9 µM) and by the absence of interference from competing metal ions and anions. The probe, which is weakly emissive in its free state (quantum yield Φ = 0.01), undergoes a dramatic fluorescence enhancement at 390 nm (λ_ex_ = 345 nm) upon binding Co^2+^, accompanied by a naked-eye-visible color change from colorless to pale yellow and a blue fluorescence turn-on under a 365 nm UV lamp. Notably, **18** operates as a “turn-on” sensor for paramagnetic Co^2+^ via CHEF, in contrast to the CHEQ behavior typically observed for paramagnetic ions; this unusual response is attributed to the rigid, highly conjugated pyrene fluorophore, which efficiently channels the absorbed energy into radiative decay.

Spectroscopic investigations, including FT-IR, HRMS, and DFT calculations, confirmed that Co^2+^ coordinates with the carbonyl oxygen, imine nitrogen, and pyrazine nitrogen atoms in a 1:1 stoichiometry. This coordination rigidifies the molecular structure, restricts non-radiative decay pathways, and is consistent with ICT, as suggested by a reduction in the HOMO–LUMO gap from 2.70 eV (free ligand) to 1.86 eV (**18**-Co^2+^); however, the gap reduction alone does not establish the ICT mechanism, which is supported by the combined spectroscopic and computational evidence. The practical utility of probe **18** is convincingly validated through quantitative recovery experiments in natural water samples (86–104% recovery), reusable test strips for on-site visual detection, and successful fluorescence imaging of intracellular Co^2+^ in HeLa cells with low cytotoxicity (cell viability >85% at 20 µM), positioning **18** as a promising tool for environmental monitoring, point-of-use testing, and biomedical diagnostics in cobalt-related health risk assessment [73].

**Figure 17:**
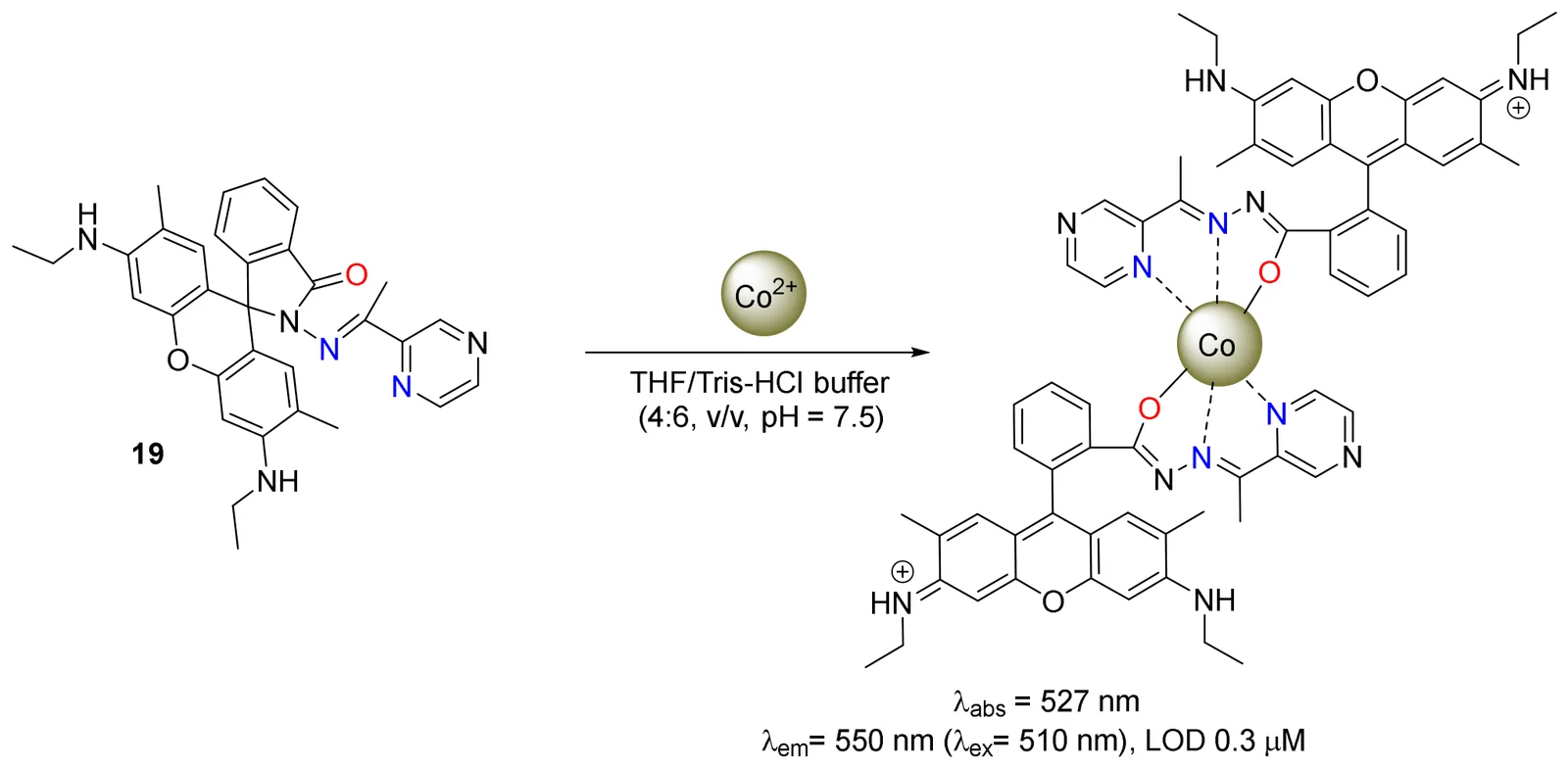
Proposed binding mode of sensor **19** with Co^2+^ upon addition of CoCl_2_. Color coding: nitrogen atoms involved in metal coordination are shown in blue; oxygen atom involved in metal coordination is shown in red.

Liu et al. reported a rhodamine 6G-based pyrazine-derived chemosensor **19** (Figure 17) designed for operation in living cells [74]. It is well established that the rhodamine lactam may transform from non-emissive spirolactam form to a fluorescent amide upon coordination; incorporation of a pyrazinyl group on the rhodamine framework enhances both the coordination ability and the water affinity of the sensor. Mechanistically, **19** operates through a chemodosimetric ring-opening process: coordination of Co^2+^ triggers ring opening of the rhodamine spirolactam, restoring the conjugated xanthene chromophore and activating CHEF. Sensor **19** displays excellent photophysical properties in the THF/Tris-HCl buffer (4:6, *v*/*v*, pH = 7.5), including a large molar absorption coefficient, long-wavelength absorption (at 527 nm, pink-orange solution) and emission (at 550 nm, pink solution), and a significant fluorescence enhancement of up to 56-fold upon Co^2+^ coordination. No other metal ions tested (Ba^2+^, Cd^2+^, Cu^2+^, Fe^3+^, Mn^2+^, Zn^2+^, Bi^3+^, Ca^2+^, Hg^2+^, Mg^2+^, Ni^2+^, Pb^2+^, Sr^2+^) exerted a noticeable influence on either the absorption or emission spectra, demonstrating the high selectivity of compound **19** toward Co^2+^. Notably, Al^3+^ was not included as a competing cation in this work [74] and in previously mentioned study on the pyrene derivative **18** [73].

The Co^2+^-induced fluorescent intensity of **19** showed an excellent linear response in the concentration range from 1 to 24 µM. The LOD was calculated as 0.31 µM based on 3σ/slope method [74]. In contrast to the pyrene derivative **18**, sensor **19** binds Co^2+^ at 2:1 stoichiometry as proposed in Figure 17, that was supported by Job’s plot analysis based on absorbance, ESI-MS, and spectrofluorimetric titration experiment.

In summary, all hydrazone-based 2-acetylpyrazine derivatives **17–19** act as N,N,O-tridentate ligands in their complexes with Al^3+^ or Co^2+^, with the pyrazine nitrogen atom participating directly in the binding process. The 2-acetylpyrazine scaffold, when functionalized with hydrazone moieties, provides a versatile platform for constructing “turn-on” fluorescent sensors. A key feature distinguishing this subclass is the direct participation of the pyrazine ring nitrogen in metal coordination, forming an N,N,O-tridentate pocket that effectively suppresses PET and C=N isomerization upon metal binding. The reported examples show high sensitivity towards Al^3+^ (LODs as low as 100 nM) and Co^2+^ (LOD down to 0.104 nM for the pyrene derivative **18**), with remarkable fluorescence enhancements of up to 230-fold. The incorporation of rigid, highly emissive fluorophores such as pyrene or rhodamine 6G further improves the photophysical properties and enables bioimaging applications. However, the selectivity of these systems is occasionally compromised by Cu^2+^ and Fe^2+^, which act as universal quenchers, suggesting that future designs should focus on optimizing the chelating cavity to minimize such interferences.

### 2.3. Aminopyrazine and Hydrazinopyrazine Derivatives

The family of 2-aminopyrazines and 2-hydrazinopyrazines, together with their heterocycle-substituted analogs, is readily accessible and can be straightforwardly modified at the amino/hydrazino group by aromatic carbonyl compounds under mild conditions [75,76]. The resulting amino-substituted derivatives (Schiff-base ligands) provide tailored structures for selective detection of a variety of analytes, including metal cations.

Aminopyrazine and hydrazinopyrazine sensors frequently operate through coordination-induced CHEF, ESIPT inhibition, or ratiometric ICT, with the pyrazine ring nitrogen directly involved in metal binding. Because the amino or hydrazino group at the 2-position provides an additional donor atom, these receptors readily form N,N,O-chelating cavities upon condensation with aromatic aldehydes or ketones. The resulting complexes are typically more rigid than those formed by carboxamide-based ligands, which is proposed to favor CHEF-based “turn-on” responses and, in several cases, enables ratiometric detection through the appearance of a new, red-shifted emission band.

**Figure 18:**
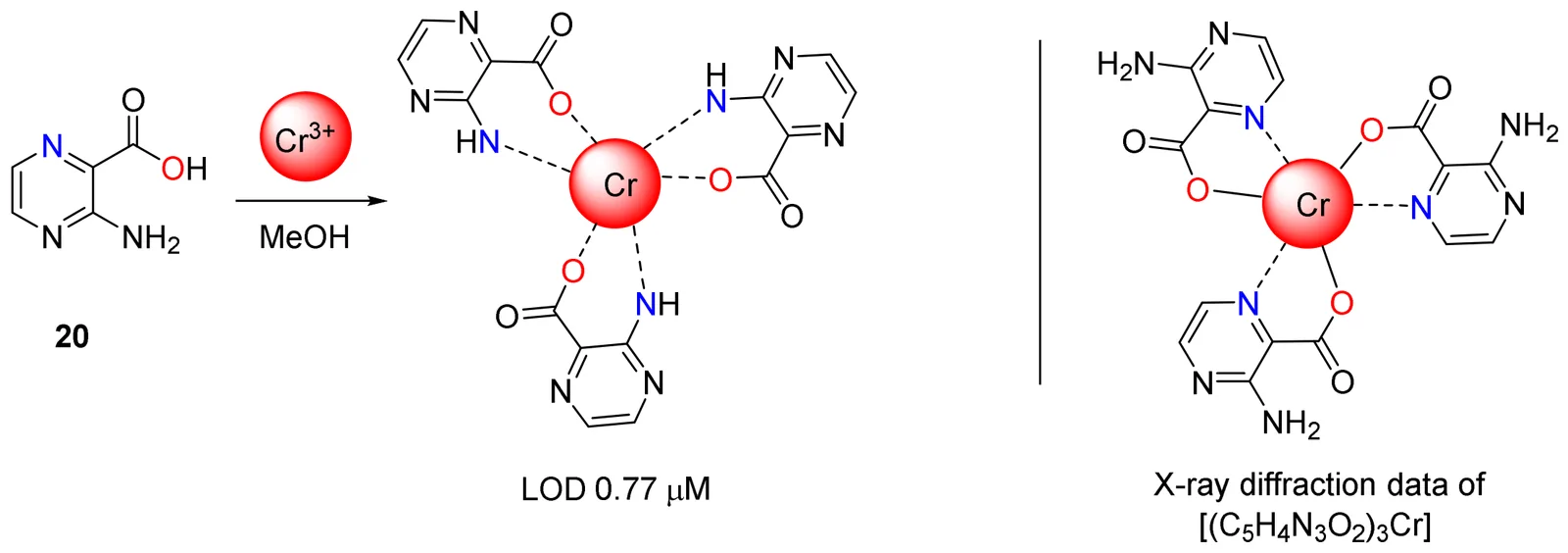
Proposed binding mode of 3-amino-2-pyrazinecarboxylic acid **20** with Cr^3+^ and the crystal structure of [(C_5_H_4_N_3_O_2_)_3_Cr] according to X-ray diffraction data. Color coding: nitrogen atoms involved in metal coordination are shown in blue; oxygen atoms involved in metal coordination are shown in red.

Notably, even non-functionalized 2-aminopyrazine exhibits sensitive behavior, as demonstrated by Chatterjee et al. [44], who reported the use of a small molecule, 3-amino-2-pyrazine carboxylic acid **20** (Figure 18), as an efficient spectrophotometric probe exhibiting a hyperchromic effect (absorption enhancement) for the selective detection of Cr^3+^ among competing cations (Al^3+^, Fe^3+^, Mn^2+^, Cu^2+^, Ni^2+^, Cd^2+^, Zn^2+^) and anions (F^−^, Cl^−^, NO_3_^−^, SO_4_^2−^, CrO_4_^2−^, Cr_2_O_7_^2−^) in MeOH. Sensor **20** operates through a coordination-induced hyperchromic effect rather than through CHEF or CHEQ; the crystal structure of the isolated Cr^3+^ complex reveals N,O-bidentate coordination, although the solution-state binding mode may differ. The hyperchromic effect should be regarded as a phenomenological description of the optical response rather than a complete mechanistic assignment. Moreover, compound **20** displays opposite spectroscopic responses toward Cr^3+^ and Cr^6^, that is important for distinguishing the highly toxic, carcinogenic, and mobile Cr^6+^ species from the less toxic, essential trace mineral Cr^3+^.

Upon increasing the Cr^3+^ ion concentration, the absorption band at 250 nm shows a concomitant increase in optical density. The LOD, calculated from the calibration curve at 250 nm using the 3σ/k method, was found to be 0.77 µM, demonstrating the high sensitivity of the sensor. Based on the spectroscopic responses (with the intensity at 350 nm as the output parameter), molecular logic gates were designed using **20** and Cr^3+^ as chemical inputs. Additionally, the ability of **20** to operate in the neutral pH range (6.0–8.0) was demonstrated, representing a promising feature for environmental monitoring.

Regarding the binding mode, the authors proposed formation of a **20**-Cr^3+^ complex through the nitrogen atom of amine moiety and the oxygen atom of the carboxylic acid group in a 3:1 stoichiometry (Figure 18). However, crystals obtained from reaction of **20** with Cr(NO_3_)_3_ × 6H_2_O revealed a in which **20** acts as an N,O-bidentate ligand coordinating through a carboxylate oxygen and a pyrazine nitrogen atom (Figure 18); spectroscopic evidence supporting the solution-state structure from UV–vis studies were not provided.

**Figure 19:**
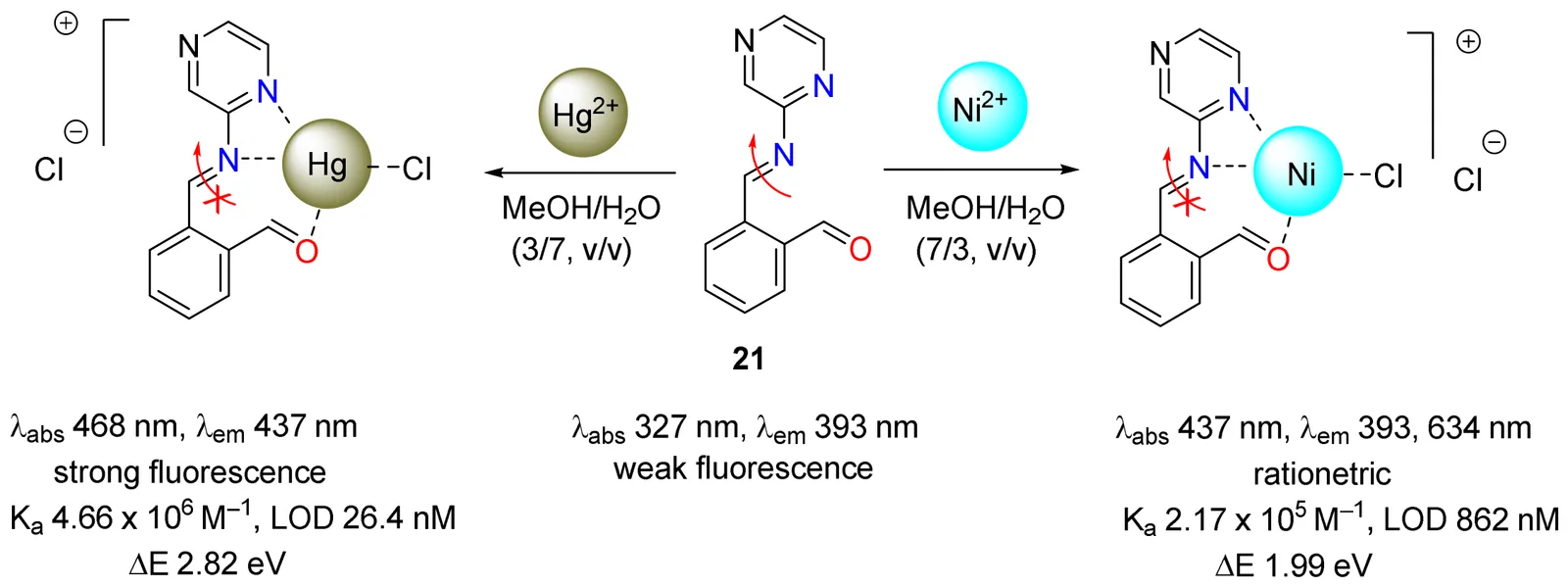
Molecular structure of sensor **21** and its proposed binding mode with Hg^2+^ and Ni^2+^ upon addition of their chlorides. Color coding: nitrogen atoms involved in metal coordination are shown in blue; oxygen atom involved in metal coordination is shown in red; the arrow indicated rotation of the molecular fragments shown in red.

Another representative aminopyrazine-derived chemosensor **21** (Figure 19) can be obtained via a simple synthetic approach by refluxing 2-aminopyrazine with phthalaldehyde in MeOH [77,78]. Kumar et al. subsequently demonstrated the ability of **21** to detect Hg^2+^ and Ni^2+^ with distinct colorimetric and fluorescent response in MeOH/H_2_O mixtures (3/7 and 7/3, *v*/*v*, respectively).

In its free state, a colorless solution of probe **21** absorbs with a maximum around 327 nm and exhibits weak fluorescence at 393 nm due to C=N isomerization in the excited state. However, the presence of Hg^2+^ or Ni^2+^ leads to the appearance of new band at around 468 nm and 437 nm, respectively, accompanied by a naked-eye color change. These results are consistent with LMCT between the ligand and the metal ion due to formation of stable chelate complexes. The fluorescence response of sensor **21** for Hg^2+^ and Ni^2+^ differ: upon addition of Hg^2+^, a bathochromic shift from 393 nm to 437 nm is observed with significant enhancement of intensity [77], whereas Ni^2+^ induces an additional emission band around 634 nm and simultaneously enhances both sensor bands at 393 and 634 nm giving ratiometric emission response [78].

Job’s plot and IR studies for both metal cations established that ligand **21** via N,N,O-coordination mode through the imine nitrogen, aldehyde oxygen, and pyrazine nitrogen atoms, forming stable 1:1 complexes, suppressing C=N isomerization, and activating the CHEF mechanism (Figure 19). According to DFT calculation, the HOMO–LUMO energy gap decreases from 4.09 eV in the free sensor **21** to 2.82 eV and 1.99 eV in the **21**-Hg^2+^ and **21**-Ni^2+^ complexes, respectively, which is consistent with ground-state electronic stabilization and rationalizes the observed spectral changes; however, this should not be interpreted as direct evidence of thermodynamic stability, which would require equilibrium binding data.

Sensor **21** exhibits excellent sensitivity, with LOD of 26.4 nM for Hg^2+^ and 0.862 µM for Ni^2+^; the latter is below the US EPA permissible limit for drinking water (1.2 μM). Moreover, Schiff base **21** shows remarkable selectivity over a wide range of competing metal ions and anions, with minimal interference from coexisting species; however, mutual interference between Hg^2+^ and Ni^2+^ was not studied. Mechanistically, the two analytes induce distinct responses: for Hg^2+^, coordination activates CHEF and produces a bathochromic shift; for Ni^2+^, a ratiometric response arises from the appearance of a new emission band at 634 nm.

Based on the color changing and spectral responses of **21**, molecular logic gates were investigated. The sensing process for Hg^2+^ is fully reversible upon addition of EDTA, enabling regeneration and recyclability; this reversible “on–off–on” switching behavior was successfully exploited to construct molecular logic functions (OR, AND, NOT, and NOR gates) and a sequential memory unit demonstrating “Writing–Reading–Erasing–Reading” and “Multi-write” activities in binary logic format. On the other hand, compound **21** can be applied to molecular logic functions with single and dual input signal amplification by employing chemical input (Ni^2+^) and light input (UV).

The practical utility of the probe **21** is convincingly validated through quantitative recovery experiments for Hg^2+^ demonstration in real water samples (tap, river, and bottled water, 72.5–108.3% recovery), convenient test-strip and silica-gel-based solid-state detection kits for on-site visual monitoring, and successful fluorescence imaging of intracellular Hg^2+^ in HeLa cells with low cytotoxicity, positioning **21** as a versatile and promising chemosensor for environmental monitoring, point-of-use testing, and biomedical diagnostics in mercury-related health risk assessment [77].

**Figure 20:**
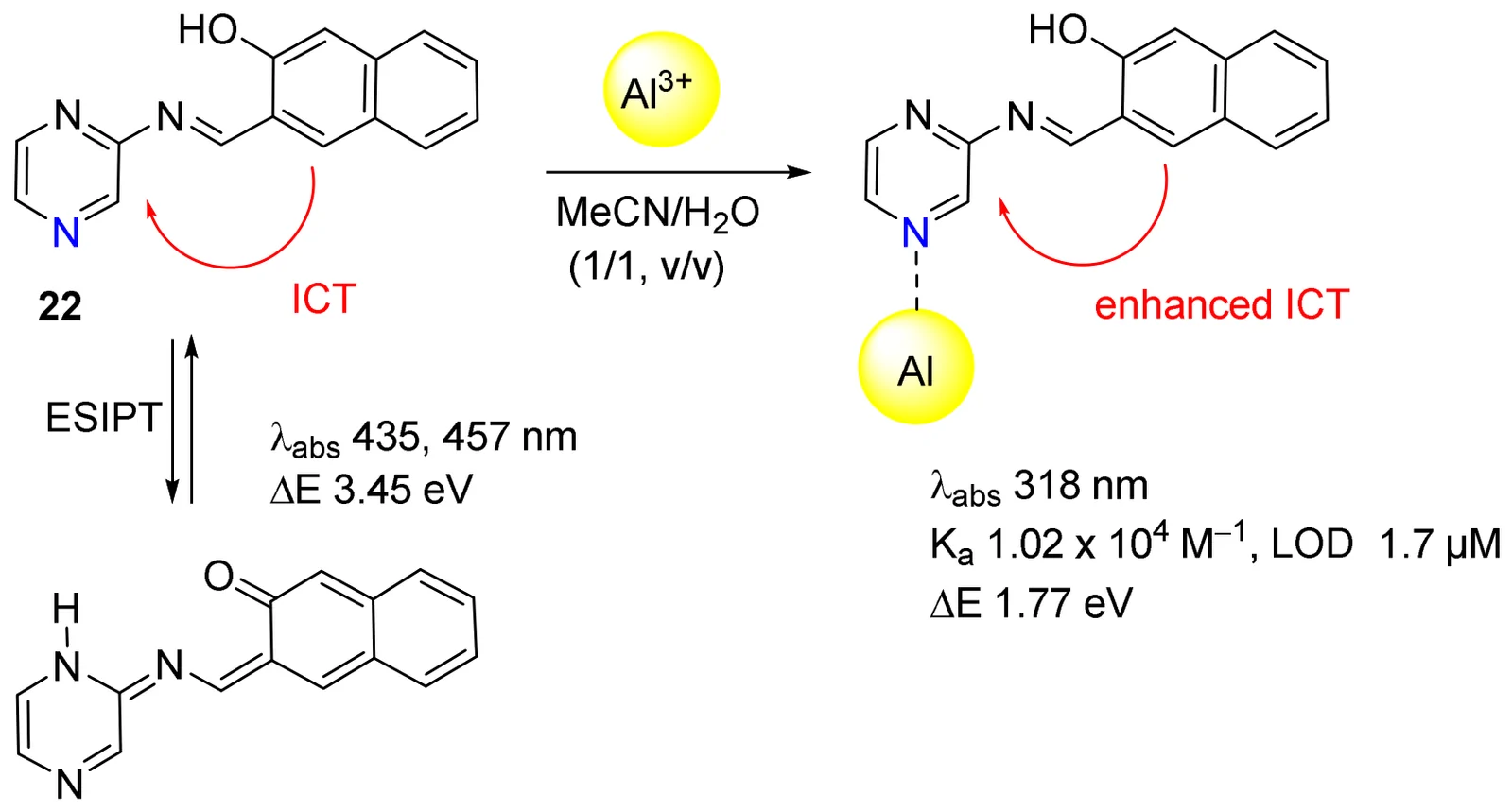
Molecular structure of sensor **22** and its proposed binding mode with Al^3+^ upon addition of Al_2_(SO_4_)_3_. Color coding: nitrogen atom involved in metal coordination is shown in blue; the proposed detection mechanism is shown in red.

Introduction of a 2-hydroxynaphthalen-1-yl fragment into 2-aminopyrazine afforded the ESIPT-active sensor **22** (Figure 20), which showed a rapid colorimetric response to Al^3+^ [79]. Owing to the extended conjugation of the receptor with the fused naphthalene ring system, the free sensor **22** absorbed at around 450 nm in CH_3_CN/H_2_O (1:1, *v*/*v*) within a physiologically relevant pH window. Selectivity studies showed that other tested metal ions (Na^+^, K^+^, Cu^2+^, Fe^3+^, Zn^2+^, Ni^2 +^, and others) had no significant effect on the absorption band or solution color, confirming the probe’s ability to detect Al^3+^ in complex systems. Moreover, sensor **22** responded rapidly and reversibly to Al^3+^, making it suitable for rapid, repeated, real-time detection.

Stoichiometric analysis by Job’s plot and mass spectrometric suggested formation of a 1:1 **22**-Al^3+^ complex (Figure 20) with strong affinity between the components (K_a_ = 1.02 × 10^4^ M^−1^); a conclusion further supported by ^1^H NMR spectroscopy and DFT calculations. The authors proposed that Al^3+^ binds monodentantly to the pyrazine nitrogen, enhancing the ICT character of the system (Figure 20). Together with ESIPT, this interaction suppresses the π–π* transitions of the naphthyl fragment, resulting in the observed colorless complex [79]. Monodentate binding of Al^3+^ to the pyrazine nitrogen enhances ICT and suppresses ESIPT, producing a colorless complex; the response is therefore best described as ESIPT/ICT dual-mechanism sensing. The probe **22** was successfully applied to test strips for practical on-site Al^3+^ detection and also used to construct a logic gate with Al^3+^ acting as a chemical input.

**Scheme 6:**
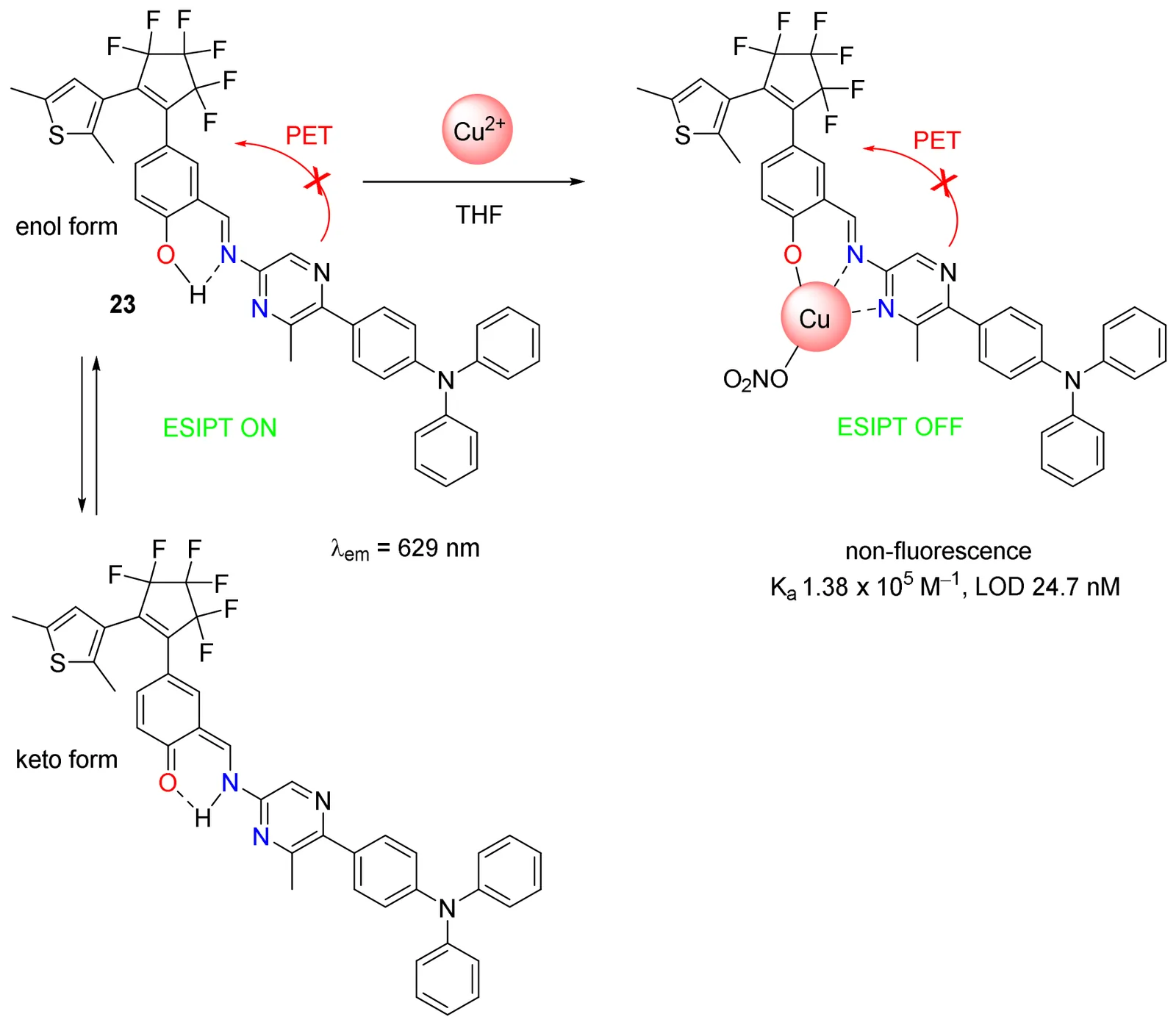
Tautomerization of sensor **23** and the proposed coordination mode with Cu^2+^ upon addition of Cu(NO_3_)_2_. Color coding: nitrogen atoms involved in metal coordination are shown in blue; oxygen atom involved in metal coordination is shown in red; the proposed detection mechanism is shown in red and green.

An original D-π-A-type fluorescent chemosensor **23** (Scheme 6) for Cu^2+^, featuring a hexafluorocyclophentene unit as an acceptor and a pyrazine unit as a donor was prepared by Fu Shi et al. [80]. Due to the presence of a mobile hydrogen atom, an ESIPT process occurs in THF solution, accompanied by inhibition of PET. The extended conjugated structure of **23** is characterized by a low-energy absorption peak at 413 nm and a broad fluorescence emission band centered at 629 nm (orange solution color under UV–light). Upon addition of 19 commonly used metal cations, only Cu^2+^ causes complete fluorescence quenching with visually detectable changes (color turns from orange to black), and no interference is observed in competing experiments. Coordination of Cu^2+^ simultaneously restricts ESIPT, activates CHEQ via paramagnetic quenching, and modulates PET, resulting in complete fluorescence quenching. Fluorescent titration supports a linear-response quenching with saturation at 2.0 equiv. of Cu^2+^ and an LOD of 24.7 nM. The quenching process is explained by a combination of ESIPT restriction due to participation of OH group in coordination, a CHEQ effect, and the paramagnetic nature of Cu^2+^. Notably, the stability of the complex **23**-Cu^2+^ is so high that addition of EDTA does not reverse the fluorescence.

According to Job’s plot analysis, ^1^H NMR spectroscopy, and ESI-MS, a 1:1 stoichiometry and an N,N,O-binding mode (Scheme 6) was proposed. The high selectivity and sensitivity were reproduced in the qualitative test-strips experiments, where Cu^2+^ induced not only selective fluorescence quenching relative to other metals but also progressive color changes from orange to dark as a function of concentration. Finally, sensor **23** can be applied to detect Cu^2+^ in practical water samples with high accuracy from 97.2% to 103.4%.

**Figure 21:**
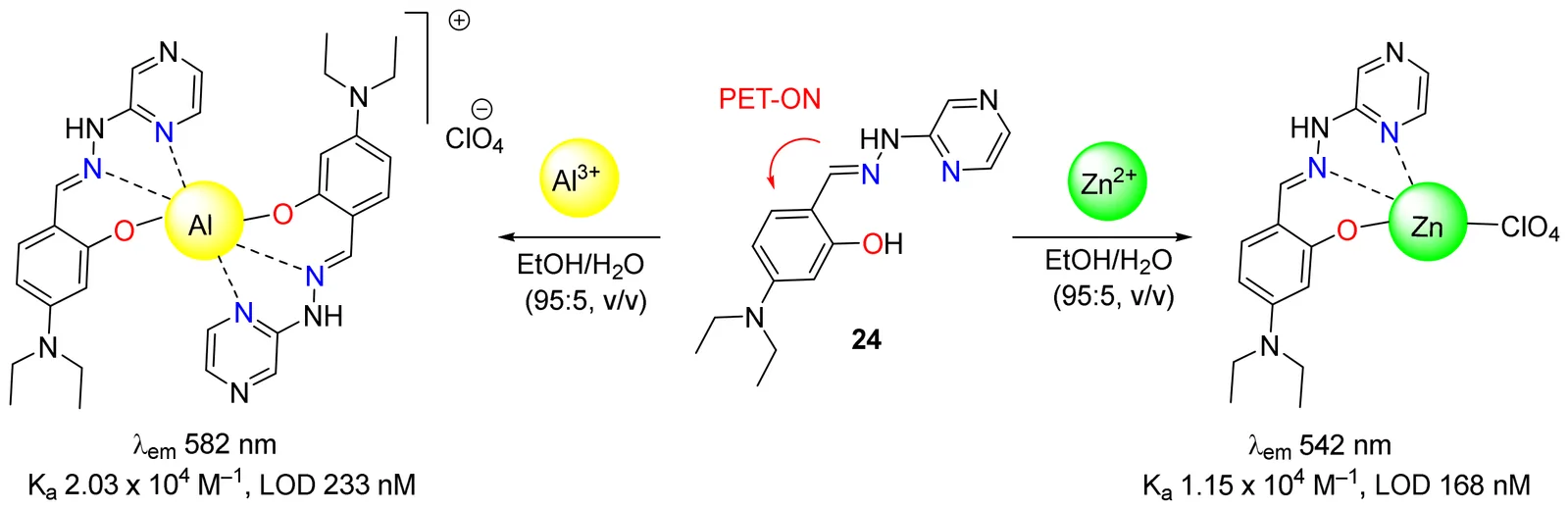
Molecular structure of chemosensor **24** and its proposed binding mode with Al^3+^ and Zn^2+^ upon addition of Al(ClO_4_)_3_ or Zn(ClO_4_)_2_. Color coding: nitrogen atoms involved in metal coordination are shown in blue; oxygen atom involved in metal coordination is shown in red; the proposed detection mechanism is shown in red.

**Figure 22:**
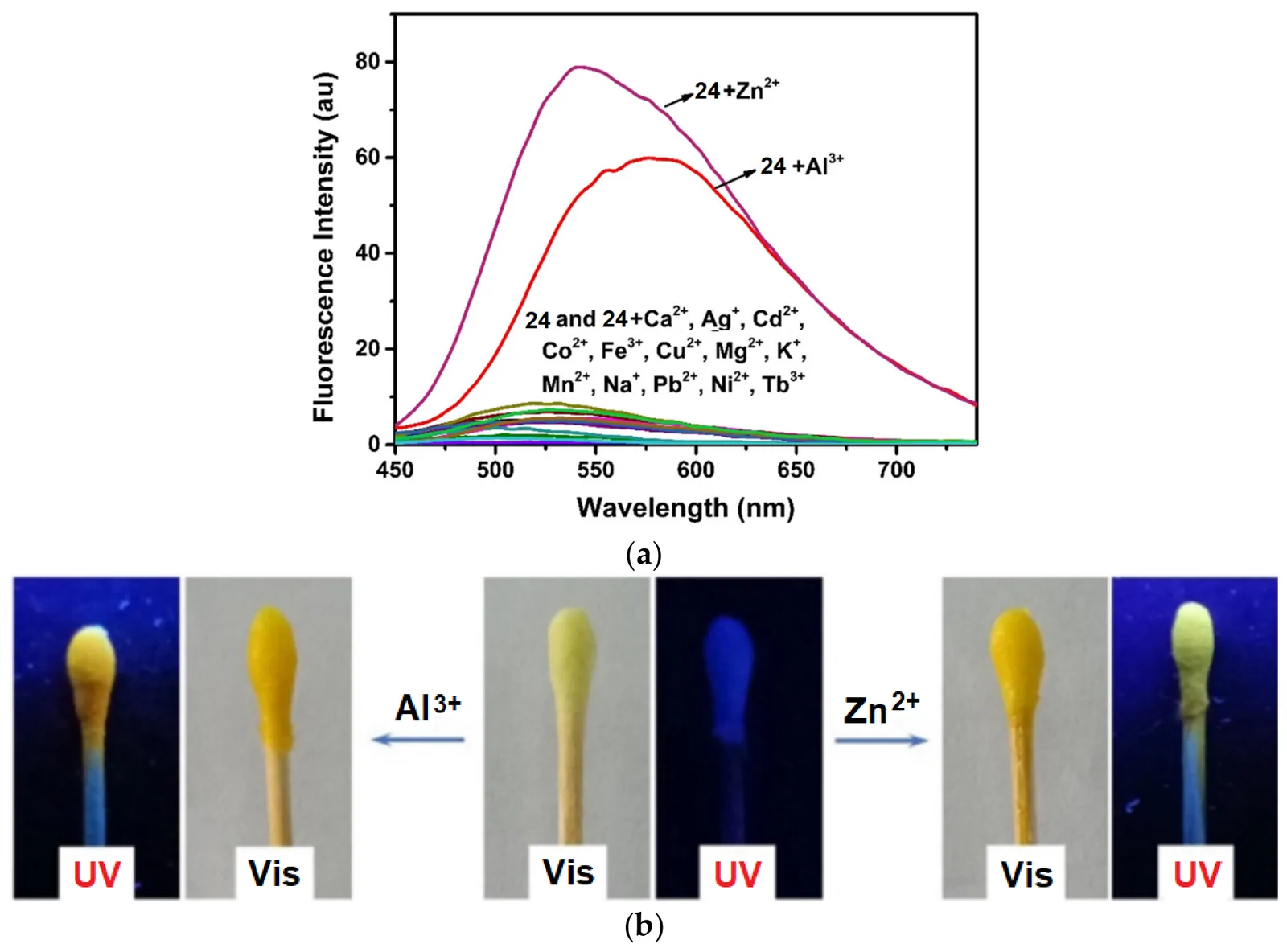
(**a**) Fluorescence spectrum changes of **24** (50 μM) on addition of various metal ions (2.0 equiv.) in ethanol–water (95:5, *v*/*v*) (λ_ex_ = 366 nm): purple line—spectrum of **24** upon addition of Zn^2+^; red line—spectrum of **24** upon addition of Al^2+^; the other lines correspond to the free sensor **24** and to its spectra upon addition of Ca^2+^, Ag^+^, Cd^2+^, Co^2+^, Fe^3+^, Cu^2+^, Mg^2+^, K^+^, Mn^2+^, Na^+^, Pb^2+^, Ni^2+^,, and Tb3^+^. (**b**) The color changes in the **24**-coated cotton swab in the absence and presence of Al^3+^ and Zn^2+^. Reproduced from [81] licensed under Creative Commons BY 4.0.

A hydrazinopyrazine derivative **24**, synthesized by condensation of 4-(diethylamino)salicylaldehyde and 2-hydrazinopyrazine in ethanol (Figure 21), acts as a fluorescent sensor for Al^3+^ and Zn^2+^ with a “turn-on” signal via the CHEF mechanism [81]. The free probe, upon excitation at 366 nm, exhibits weak emission due to the PET process. Coordination with Al^3+^ or Zn^2+^ via the imine nitrogen, phenolic oxygen, and pyrazine nitrogen suppresses PET and activates CHEF, resulting in significant fluorescence enhancements at different wavelengths–582 nm for Al^3+^ and 542 nm for Zn^2+^ (Figure 22a)–enabling dual-ion recognition.

Job’s plot, mass spectrometry, UV–Vis, NMR, and FT-IR data suggest that chemosensor 24 binds Al^3+^ in a 2:1 stoichiometry and Zn^2+^ in a 1:1 stoichiometry (Figure 21). Coordination is mediated by the phenolic oxygen, imine nitrogen, and pyrazine nitrogen atoms. Sensor 24 therefore acts as a dual-ion CHEF probe, binding Al^3+^ in a 2:1 and Zn^2+^ in a 1:1 stoichiometry, with PET suppression and CHEF activation responsible for the observed emission enhancements. The sensor **24** demonstrates high selectivity over competing metal ions (except for Cu^2+^ and Fe^2+^) and synergetic effect between Al^3+^ and Zn^2+^. Reversibility was established by multiple alternate addition of EDTA and Me^2+^, with detection limits of 0.233 µM for Al^3+^ 0.168 µM for Zn^2+^, both lying below the enforceable drinking water standards proposed by the WHO (7.4 µM for Al^3+^ and 76 µM for Zn^2+^). Practical applicability was demonstrated in real water and drug samples, as well as on coated swabs for naked-eye detection (Figure 22b).

Aminopyrazine and hydrazinopyrazine derivatives offer a distinct advantage in terms of synthetic accessibility and structural diversity. The presence of an additional amino or hydrazino group at the 2-position of the pyrazine ring creates a versatile N,N,O-chelating center when combined with suitable carbonyl or hydroxyl substituents. This class has produced sensors for a wide range of cations, including Cr^3+^, Hg^2+^, Ni^2+^, Al^3+^, Zn^2+^, and Cu^2+^, with detection limits ranging from nanomolar to micromolar levels. A notable trend is the frequent involvement of the pyrazine nitrogen in metal binding, which is proposed to enhance the rigidity of the complex and is thought to activate CHEF or ESIPT mechanisms. The practical utility of these sensors has been convincingly demonstrated through real-water analysis, test-strip-based naked-eye detection, and live-cell imaging. However, the selectivity remains a concern: many aminopyrazine-based probes respond to multiple metal ions, and the development of truly specific sensors for individual cations requires further rational design, particularly through the introduction of sterically or electronically tuned substituents.

### 2.4. Aryl and Hetaryl Pyrazines

This section covers aryl(hetaryl)-substituted pyrazines, excluding MOF-based materials. While pyrazinecarboxamides, hydrazones, and aminopyrazines predominantly rely on N,N,O-chelating pockets for metal binding, aryl- and heteroaryl-substituted pyrazines exploit a different design principle. In this class, the pyrazine core is directly conjugated with aromatic or heteroaromatic substituents, which extends the π-system, modulates the frontier orbital energies, and in many cases facilitates AIE in mixed aqueous–organic media. The absence of flexible hydrazide or amide linkers often results in more rigid structures, where metal coordination occurs primarily through the nitrogen atoms of the pyrazine and/or the substituents, rather than through peripheral donor groups. This structural simplicity, combined with synthetic accessibility via transition-metal-catalyzed cross-couplings or oxidative cyclisation, makes arylpyrazines attractive scaffolds for the development of cost-effective, solid-state sensors. The reported examples show selective “turn-off” fluorescence responses toward Fe^3+^, Pb^2+^, Cu^2+^, and Co^2+^, with detection limits in the micromolar range and successful application in real water samples and on test strips. In contrast to the MOF-based materials incorporating polytopic pyrazine linkers, the discrete small molecules discussed herein offer the advantages of straightforward synthesis, well-defined molecular structures, and solution-processability. This section provides a concise overview of the current literature on aryl- and heteroaryl-substituted pyrazines as cation chemosensors, highlighting their structural diversity, sensing performance, and remaining challenges.

Aryl- and hetaryl-substituted pyrazines predominantly exhibit “turn-off” responses based on PET, AIE disruption, or FRET, with the pyrazine ring acting as the primary coordination site. In contrast to the carboxamide- and hydrazide-based receptors discussed above, these sensors lack flexible amide or hydrazone linkers; metal coordination therefore occurs mainly through the nitrogen atoms of the pyrazine core and/or the conjugated substituents. The extended π-system of these molecules shifts absorption and emission to longer wavelengths and, in mixed aqueous–organic media, promotes AIE, which can be modulated by analyte binding.

**Figure 23:**
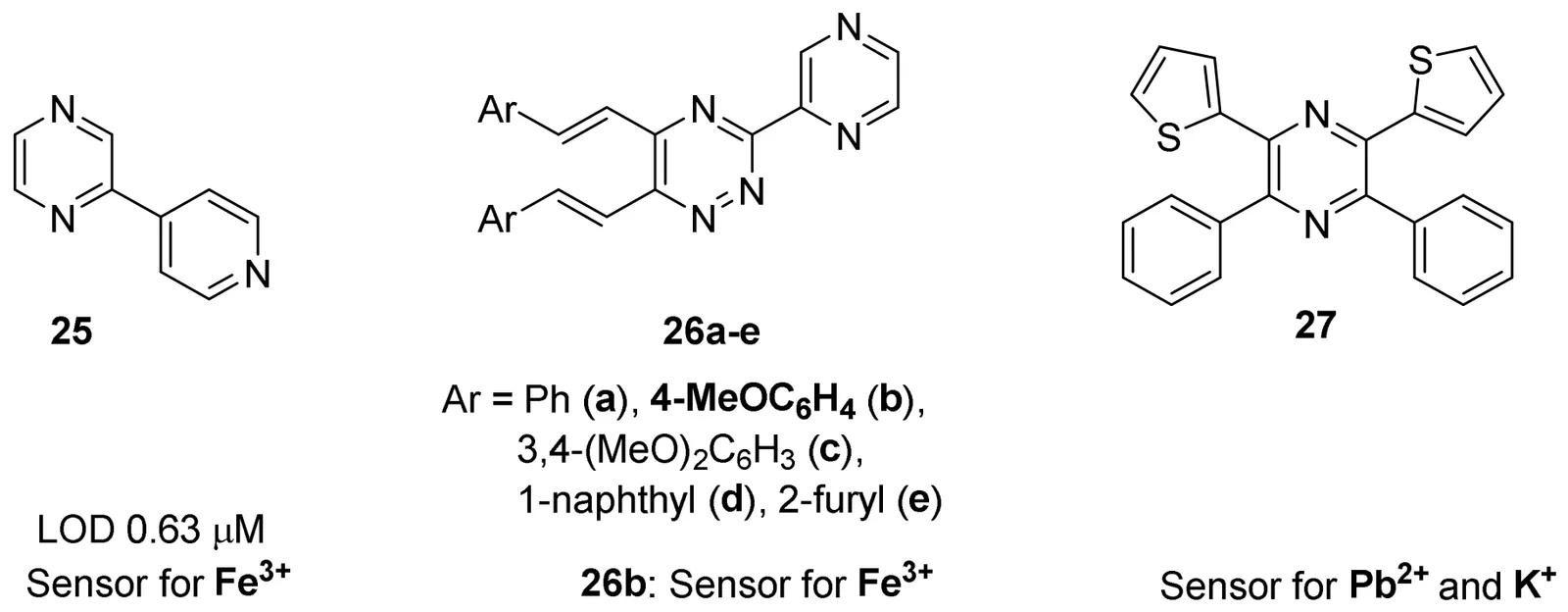
Molecular structure of sensors **25**–**27**.

Aryl and heteroaryl substituted pyrazines, including (pyridine-4-yl)pyrazine **25** (Figure 23), are synthesized via a tandem imine formation–oxidative cyclization between the corresponding aryl/heteroaryl methylketones and 1,2-diamines under an oxygen atmosphere [82]. Compound **25** demonstrated selective and specific recognition of Fe^3+^ by quenching its emission at 440 nm. In addition, based on the linear response in the 0.5–15 μM range and a micromolar limit of detection (0.63 μM), this probe was used to measure Fe^3+^ concentration in tap and drinking water, yielding satisfactory recovery rates in the range of 92.69–105.49%. Mechanistically, **25** operates as a “turn-off” fluorescent probe for Fe^3+^, presumably through paramagnetic quenching (CHEQ) by the d^5^ Fe^3+^ ion; however, the exact mechanism was not established in the original report and warrants further investigation.

Another group of mono-substituted pyrazine derivatives **26** bearing triazinyl fragment (Figure 23) was synthesized by condensation of 1,6-diphenylhexa-l,5-diene-3,4-dione with pyrazine-2-carboxtrisamidrazone in boiling methanol and can be considered as promising chemosensors [83]. Similarly to **25**, counterpart **26b** appears to be sensitive toward Fe^3+^. Upon the addition of Fe^3+^ ions at the micromolar concentration level to a solution of compound **26b** in acetonitrile, a distinct alteration in its fluorescent behavior was observed, with the initial green emission shifting to a reddish-brown color. The binding selectivity was corroborated by the absence of similar spectral changes in the presence of other tested metal species, including Fe^2+^, Cu^2+^, and Co^2+^, thereby confirming the specificity of derivative **26b** for ferric ions [83]. The “turn-off” response of **26b** towards Fe^3+^ is attributed to CHEQ/paramagnetic quenching, although the precise binding mode and the contribution of other non-radiative pathways remain to be clarified.

2,6-Diphenyl-3,5-di(thiophen-2-yl)pyrazine **27** (Figure 23) and a series of tetraarylpyrazines were synthesized by a Ni-catalyzed dimerization of 2*H*-azirines, and their potential as turn-off fluorescence probes was demonstrated using derivative **27** as an example [84]. The aggregated system of **27** in the H_2_O/THF (9:1, *v*/*v*) mixture exhibited emission at 390 nm, which was quenched in the presence of Pb^2+^ and K^+^, presumably due to disruption of the aggregate structure and enhancement of non-radiative decay. Quenching by Pb^2+^ and K^+^ is attributed to disruption of the AIE-active aggregates, which enhances non-radiative decay; the mechanism therefore differs fundamentally from classical CHEQ.

**Figure 24:**
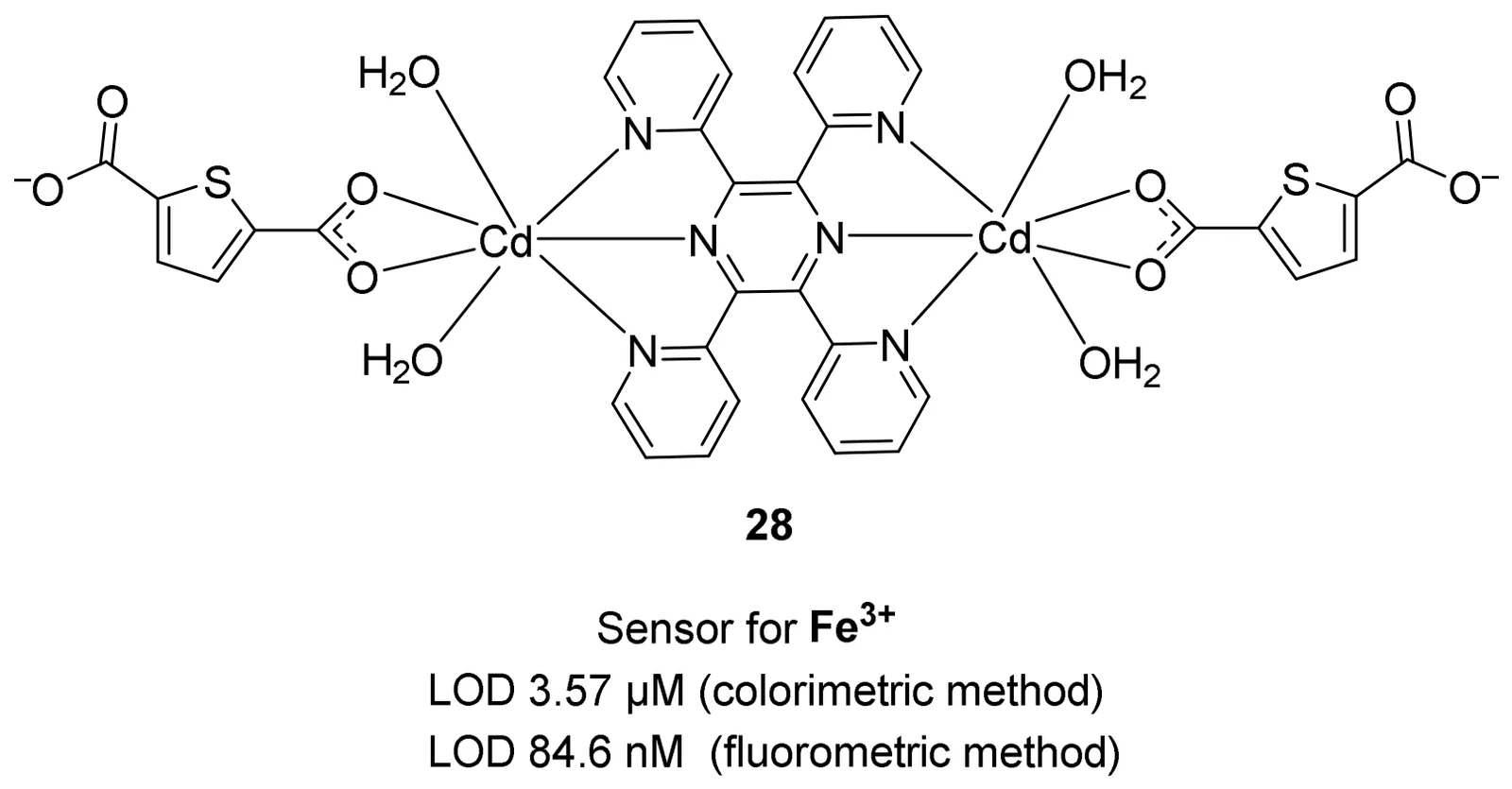
Molecular structure of dual-mode coordinated chemosensor **28** based on pyrazine ligand.

The binuclear Cd^2+^ complex **28** (Figure 24) obtained from (tetrapyridin-2-yl)pyrazine and 2,5-thiophene dicarboxylate has emerged as a dual-mode chemosensor for the detection of Fe^3+^ in DMF/H_2_O (3/7, *v*/*v*) with HEPES (*N*-2-hydroxyethylpiperazine-*N*′-2-ethanesulfonic acid) as a buffer [85]. The presence of Fe^3+^ causes a change in solution color from colorless to pink and appearance of red-shifted absorption peak near 520 nm (shifted by 195 nm), whereas no competing metal cation, including bivalent (Zn^2+^, Cu^2+^, Fe^2+^, Hg^2+^, Co^2+^, Ni^2+^, Mn^2+^, Pd^2+^, Pb^2+^) or a broad range of trivalent cations (Cr^3+^, Al^3+^, Gd^3+^, Ru^3+^, Ir^3+^, Eu^3+^, Sm^3+^) interferes with the absorption spectrum, making **28** a selective chemosensor. The appearance of this band is proposed to arise from ICT process arising from complexation. Compound **28** demonstrates a high level of efficiency and selectivity in the detection of Fe^3+^, with an LOD of 3.57 µM, a broad pH range of 2–12 and, no anionic interference, and high sustainability after six cycles of consecutive addition of Fe^3+^ and EDTA.

Moreover, probe **28** displays selective fluorescence quenching by Fe^3+^ upon excitation at 325 nm and titration experiments reveal even higher sensitivity (LOD of 0.0846 µM or 84.6 nM) compared to the colorimetric method. Sensing by **28** is proposed to involve ICT and FRET, with Stern–Volmer analysis revealing a transition from static to dynamic quenching upon increasing analyte concentration and a high quenching constant (K_SV_ = 1.57 × 10^5^ M^−1^). A significant spectral overlap of **28** and its **28**-Fe^3+^ complex indicates a strong interaction driven by the FRET mechanism. The **28**-Fe^3+^ composite exhibits enhanced excited-state stability, with a lifetime roughly ten times longer than that of the free ligand **28**.

X-Ray photoelectron spectroscopy analysis suggests that Fe^3+^ interacts specifically with the free carboxylate oxygen atoms of the sensor **28**, causing notable binding energy shifts, while nitrogen, cadmium, and sulfur components remain chemically inert.

In contrast to the functionalized pyrazine derivatives discussed above, aryl- and hetaryl-substituted pyrazines exploit a simpler design principle: direct conjugation of the pyrazine core with aromatic or heteroaromatic substituents. This approach extends the π-system, modulates frontier orbital energies, and, in some cases, facilitates AIE in mixed aqueous–organic media. The reported examples are predominantly ‘turn-off’ sensors for Fe^3+^ and Pb^2+^, with detection limits in the micromolar range. While this class offers the advantages of rigid molecular structures, straightforward synthesis via cross-coupling or oxidative cyclization, and solution-processability, it remains relatively underexplored compared to the hydrazide/carboxamide derivatives. The limited number of examples and the absence of systematic structure–activity relationship studies suggest that aryl-substituted pyrazines represent a promising, yet largely untapped, direction for the development of cost-effective and solid-state sensor materials.

### 2.5. Other Substituted Derivatives of Pyrazine

Other substituted pyrazine derivatives also represent an important class of fluorescent chemosensors with tunable sensing properties. Although most pyrazine-based sensors are discrete small molecules, the same design principles have been extended to macromolecular platforms. The two examples discussed in this section—ditopic xanthenone sensors **29a–c** and carbazole–pyrazine conjugated polymers **30a**–**c**—illustrate how the pyrazine moiety can be incorporated into more complex architectures without losing its dual role as a coordination site and an electronic modulator. The sensors in this section are proposed to operate through PET suppression (**29a**–**c**) or PET-based static quenching (**30a**–**c**), illustrating the versatility of the pyrazine scaffold beyond classical chelating motifs. In the ditopic xanthenone sensors **29a**–**c**, the pyrazine fragment lowers the pKa of the peripheral amine donors and reduces the apparent Zn^2+^ affinity, thereby suppressing background emission and improving the “turn-on” response. In the conjugated polymers **30a**–**c**, PET between the carbazole donor and the pyrazine acceptor provides a sensitive transduction pathway for Cd^2+^ detection.

**Figure 25:**
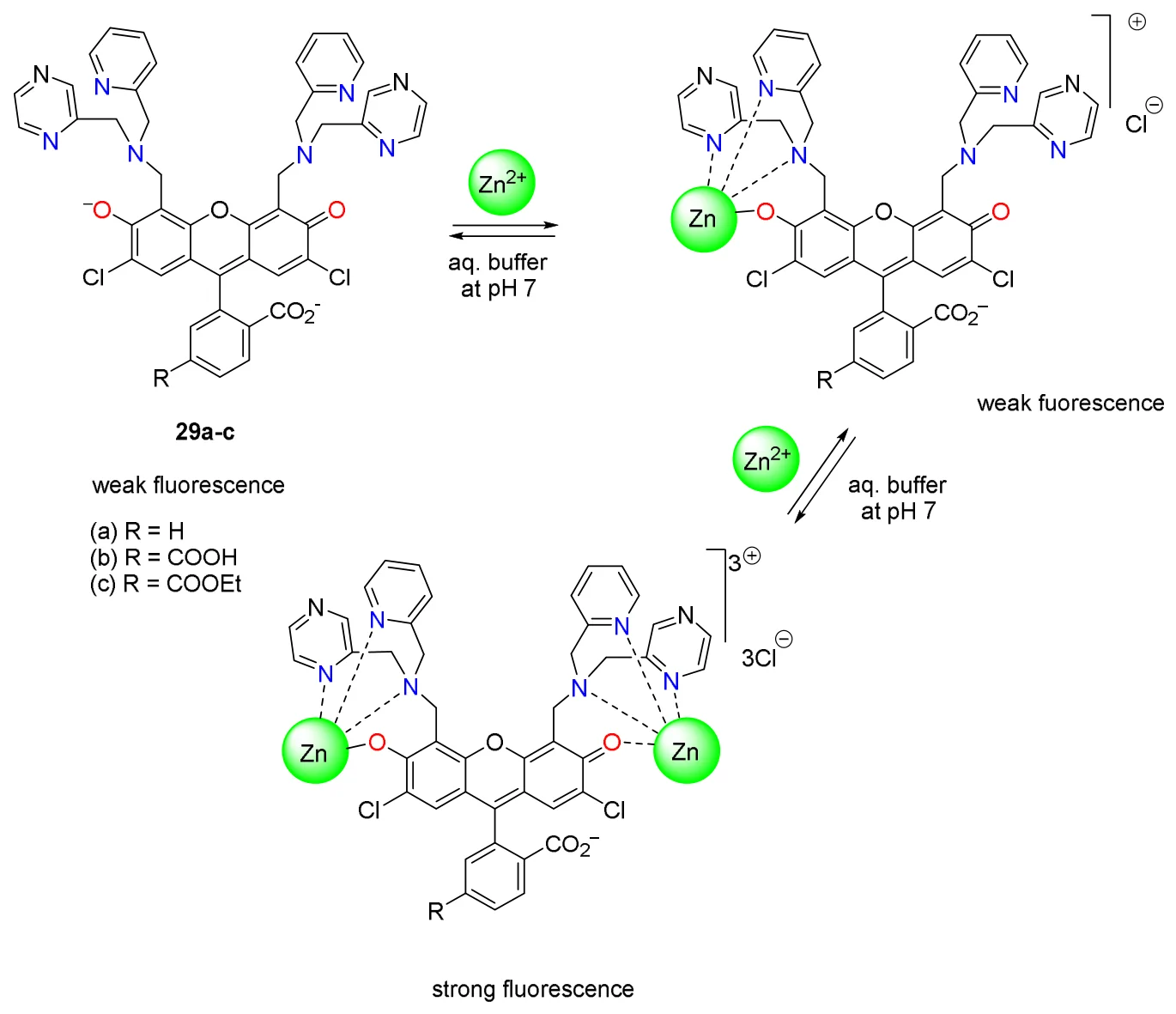
Molecular structure of fluorescein-based chemosensors **29a**–**c** and the proposed Zn^2+^-binding upon addition of ZnCl_2_ at pH 7.0. Color coding: nitrogen atoms involved in metal coordination are shown in blue; oxygen atoms involved in metal coordination are shown in red.

Xanthenone systems **29a**–**c**, functionalized with di(2-picolyl)amine and (2-picolyl)-(pyrazin-2-ylmethyl)amine fragments (Figure 25), were reported as ditopic fluorescent sensors for Zn^2+^ [86,87]. The presence of the pyrazine fragment was found to be essential, as it lowers the pKa and reduces the apparent Zn^2+^ affinity, as demonstrated by competitive studies of compound **29a** and its derivatives bearing two di(2-picolyl)amino groups; by replacing a pyridine unit with a pyrazine moiety, probe **29a** shows reduced background emission and an improved turn-on response compared to related compound [86]. Probes **29a**–**c** exhibit a unique two-step fluorescence response upon binding one versus two equivalents of zinc, enabling quantification of mobile zinc from the position of the titration maximum. Mechanistically, binding of one and then two Zn^2+^ ions sequentially is proposed to suppress PET and is thought to activate CHEF, enabling quantification of mobile zinc from the position of the titration maximum. In aqueous buffer (pH 7) the probes **29a**–**c** are non-fluorescent because PET from the peripheral amine donors to the excited xanthone core quenches emission (Φ < 0.05). Addition of Zn^2+^ (saturation at 2 equiv.) coordinates to the azine nitrogens and likely the phenolic oxygen, inducing a hypsochromic absorbance shift and suppressing PET. Metal binding therefore restores radiative decay and raises the fluorescence quantum yield. The process is reversible: strong chelators displace Zn^2+^ and recover the original spectral and emissive properties.

Selectivity studies showed that alkali and alkaline earth metal cations had no effect on fluorescence, whereas the paramagnetic transition metals Co^2+^, Ni^2+^, and Cu^2+^ caused complete and effectively irreversible quenching. Fe^2+^, Mn^2+^, Cd^2+^ and Hg^2+^ also interfered with the emission, but this effect was reversible and could be suppressed by addition of Zn^2+^ through displacement.

The authors focused on developing and analyzing ditopic fluorescent sensors **29a**–**c** for the quantitative determination of mobile Zn^2+^ in biological systems, and suggested that the same principles could be extended to other ditopic sensors for related analytes.

**Figure 26:**
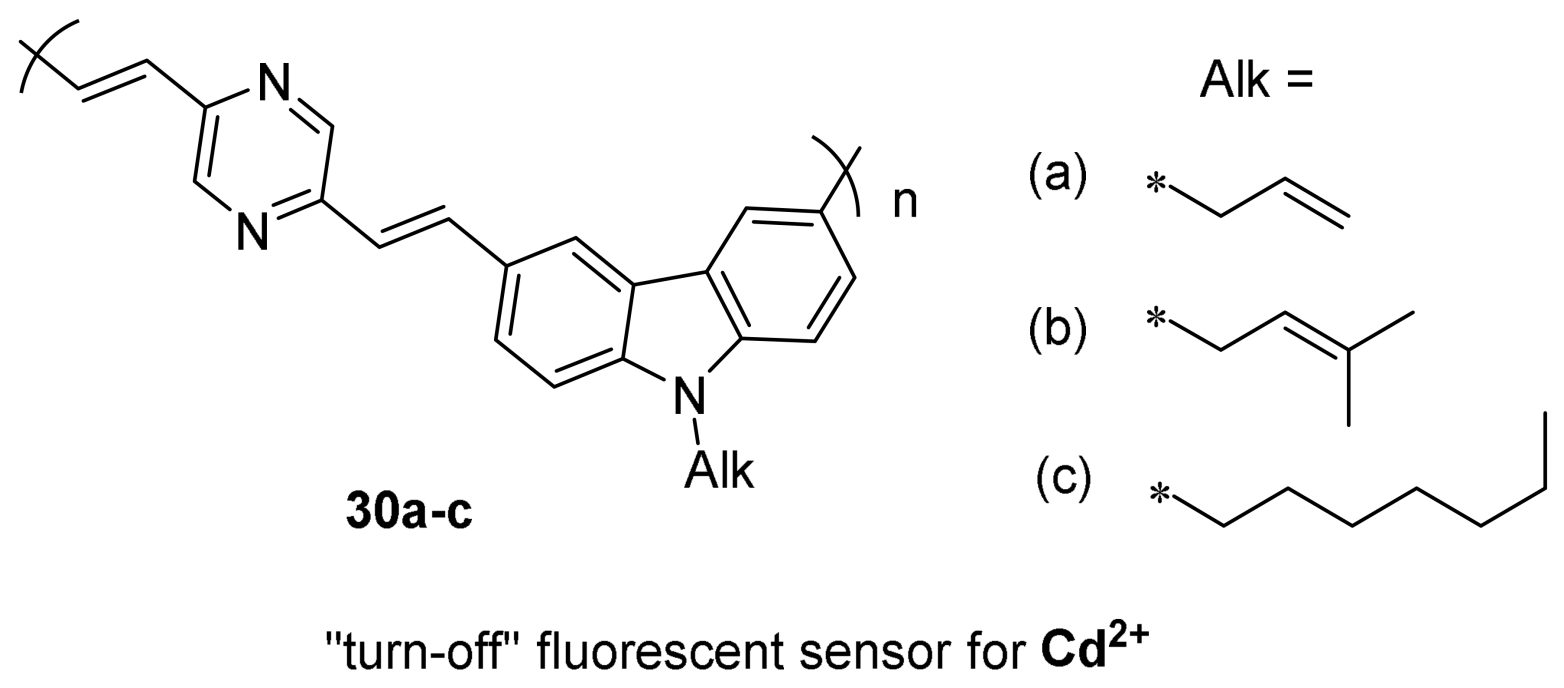
Molecular structure of carbazole functionalized pyrazine conjugated polymers **30a**–**c**.

π-Conjugated polymers **30a**–**c**, containing donor carbazole and acceptor pyrazine motifs (Figure 26), were synthesized by Wittig condensation [88]. In addition to their solvatochromic behavior, high thermal stability, and nanometer-level surface roughness—properties promising for optical applications such as light-emitting diodes or photovoltaics—these polymers exhibit fluorescent sensing ability toward Cd^2+^, with selectivity over various metal ions (Li^+^, Na^+^, K^+^, Mg^2+^, Ca^2+^, Mn^2+^, Ni^2+^, Cd^2+^, Cu^2+^, Zn^2+^, and Hg^2+^). Addition of Cd^2+^ solution to polymers **30a**–**c** in a THF/H_2_O mixture (1/1, *v*/*v*) leads to quenching of the fluorescent intensity due to PET between the heterocycles (carbazole and pyrazine) and Cd^2+^. Cd^2+^ detection proceeds via PET-based static quenching between the carbazole donor and the pyrazine acceptor, as suggested by the linear Stern–Volmer relationship.

Fluorescent titration experiments demonstrated that all three polymers can detect micromolar concentrations of Cd^2+^. Their fluorescence quenching followed a linear Stern–Volmer relationship, indicative of static quenching. The K_sv_ value was in the range of 1.8–2.3 × 10^4^ M^–1^ with LODs of 9, 13, and 8 µM, respectively.

These examples highlight the versatility of pyrazine-based platforms and underscore their strong potential for the rational design of advanced metal-ion sensors. Beyond the main structural classes, several other pyrazine derivatives have been explored as cation sensors, including xanthenone-based ditopic systems, π-conjugated polymers, and carbazole-functionalized materials. These examples highlight the remarkable structural versatility of the pyrazine scaffold, which can be integrated into complex architectures ranging from small-molecule fluorophores to macromolecular systems. The ditopic sensors **29a**–**c** demonstrate the potential for quantitative zinc detection in biological systems through a two-step fluorescence response, while the conjugated polymers **30a**–**c** illustrate the possibility of extending pyrazine-based sensing to polymer platforms. Although these systems are less numerous in the literature, they underscore the broad applicability of pyrazine chemistry beyond conventional small-molecule sensors and suggest that future developments may benefit from interdisciplinary approaches combining organic synthesis, polymer chemistry, and supramolecular design.

Inspection of the literature reveals that the sensing performance of pyrazine-based chemosensors is intimately linked to the structural motif of the ligand. Pyrazine carboxamides and hydrazides predominantly operate through N,N,O-tridentate chelation, with the amide/hydrazide carbonyl providing an additional donor atom that enhances binding affinity and selectivity. In contrast, aminopyrazine and hydrazinopyrazine derivatives often involve the pyrazine ring nitrogen directly in metal coordination, forming more rigid complexes that favor CHEF-based “turn-on” responses. Aryl- and hetaryl-substituted pyrazines, while structurally simpler, offer extended conjugation and AIE properties that are advantageous for solid-state and aggregation-based sensing. Annelation further expands the photophysical toolbox by shifting emission to longer wavelengths and introducing additional coordination sites. The following sections provide a detailed examination of each structural class, highlighting both the common principles and the unique features that govern their sensing behavior.

## 3. Annelated Pyrazines

### 3.1. Pyrazines Annelated with Five-Membered Carbo(hetero)cycle

Annelation is a useful strategy for tuning molecular properties, particularly for shifting the absorption maximum to longer wavelengths through extension of the π-conjugated system. Annelated pyrazines exploit coordination-induced ICT, charge-transfer quenching, or Lewis-acid coordination, with the extended π-system shifting the response to longer wavelengths. Fusion of the pyrazine core with five-membered carbo- or heterocycles increases the electron-accepting character of the ring and provides additional coordination sites, while the rigidified polycyclic framework suppresses non-radiative decay. The reported examples are proposed to operate through distinct mechanisms, ranging from colorimetric ICT transitions (**31**) and charge-transfer quenching (**32a–c**) to Lewis-acid-induced spectral shifts (**33, 34**) and electron/energy-transfer quenching in conjugated polymers (**35a,b**). The annelated pyrazine scaffold has also been incorporated into conjugated polymers. The two thieno[3,4-*b*]pyrazine-based copolymers **35a,b** discussed below are included here because they exploit the same electron-accepting character of the pyrazine ring and the same electron/energy-transfer quenching mechanisms as their small-molecule counterparts, thereby illustrating the generality of the design principles discussed in this review.

**Figure 27:**
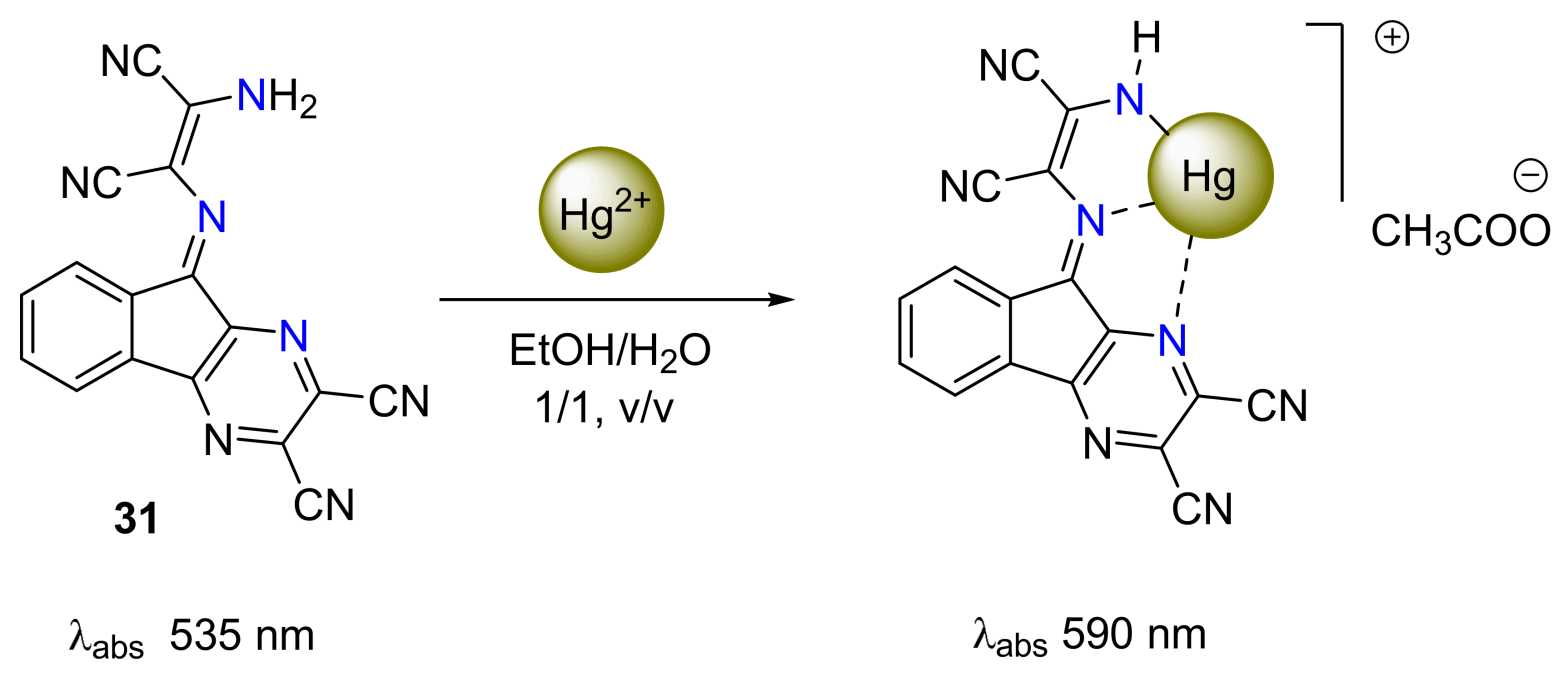
A ninhydrin-based chemosensor **31** and its proposed binding mode with Hg^2+^ upon addition of (CH_3_COO)_2_Hg. Color coding: nitrogen atoms involved in metal coordination are shown in blue.

Pyrazine sensor **31** (Figure 27), prepared by condensation of ninhydrin with diaminomaleonitrile in EtOH [89], functions as a selective and operationally simple molecular switch for the synergetic detection of Hg^2+^ and CH_3_COO^−^/F^−^ in an EtOH/H_2_O mixture, producing a distinct colorimetric transition from purple to blue. The free receptor **31** shows an absorption maximum at 535 nm, which undergoes a bathochromic shift to 590 or 570 nm (depending on the experiment conditions) upon coordination with Hg^2+^ in the presence of acetate or fluoride anion. Coordination of Hg^2+^ in the presence of acetate or fluoride induces an ICT transition, shifting the absorption maximum from 535 to 590 nm; the sensor therefore operates through coordination-induced ICT rather than through CHEF or CHEQ. The probe exhibits a high binding constant of (2.85 ± 0.38) × 10^6^ M^−1^ and selective response over both cation and anion spacers. Moreover, the sensing response is reversible (by subsequent addition of Hg(CH_3_COO)_2_ and tetrabutylammonium iodide) and can be cycled multiple times without loss of sensitivity. From ^1^H NMR, IR and UV-vis spectral titration studies, Hg^2+^ probably coordinates with **31** in a 1:1 stoichiometry via the amino group (deprotonated by Hg(CH_3_COO)_2_) and the nitrogen atoms of the imine group and pyrazine ring (Figure 27). UV–vis data indicate that chemosensor **31** exhibits selective AND-type logic behavior for the specified inputs.

**Figure 28:**
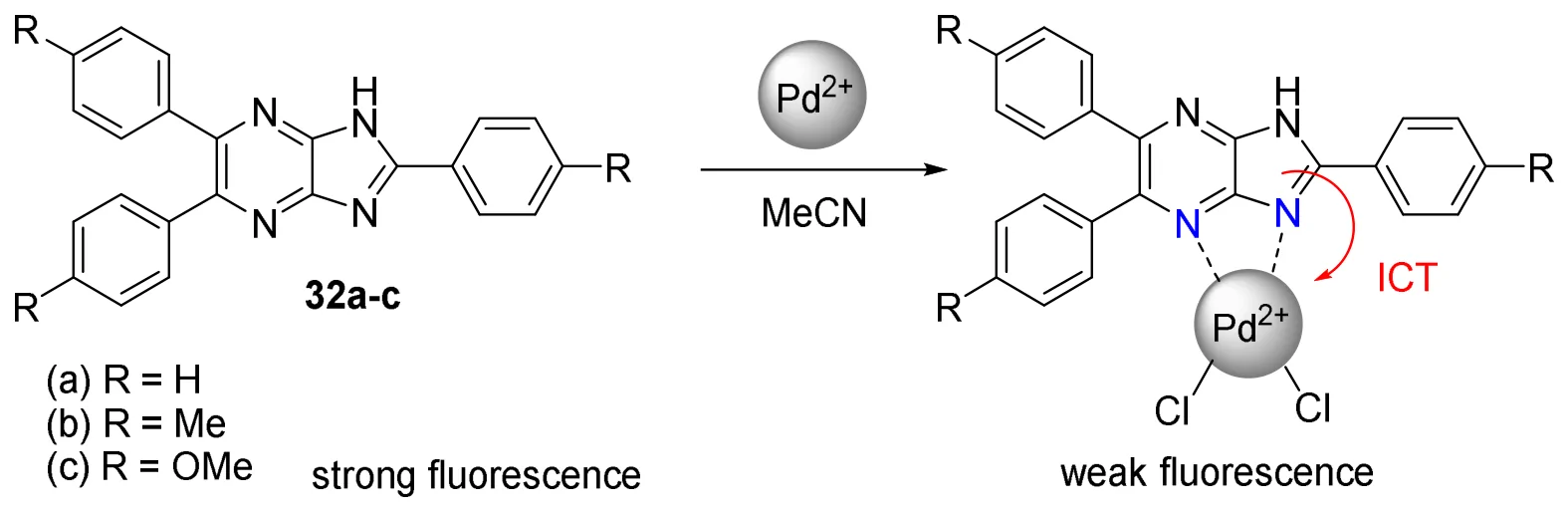
Molecular structure of 1*H*-imidazo[4,5-*b*]pyrazine sensor **32a**–**c** and proposed binding mode with Pd^2+^ upon addition of PdCl_2._ Color coding: nitrogen atoms involved in metal coordination are shown in blue; the proposed detection mechanism is shown in red.

Later, Y. Zi et al. reported triaryl 1*H*-imidazo-annelated pyrazine derivatives **32a**–**c** (Figure 28) acting as fluorescent sensors for palladium species (Pd^2+^/Pd^0^) through a fluorescence quenching response [90]. Palladium sensing proceeds via charge transfer from the sensor to the metal, resulting in efficient fluorescence quenching that is independent of the palladium oxidation state. Coordination of palladium induces charge transfer from the sensor to the metal, which is proposed to lead to a significant decrease in emission intensity. This response is highly selective, as other metal ions (including Hg^2+^ and Pt^2+^) do not cause comparable quenching, and the effect is independent of the palladium oxidation state, ligands, or counter-anion. A 1:1 binding stoichiometry between the sensor and Pd^2+^ was established (Figure 28), and the system operates over a pH range of 3–7.

**Figure 29:**
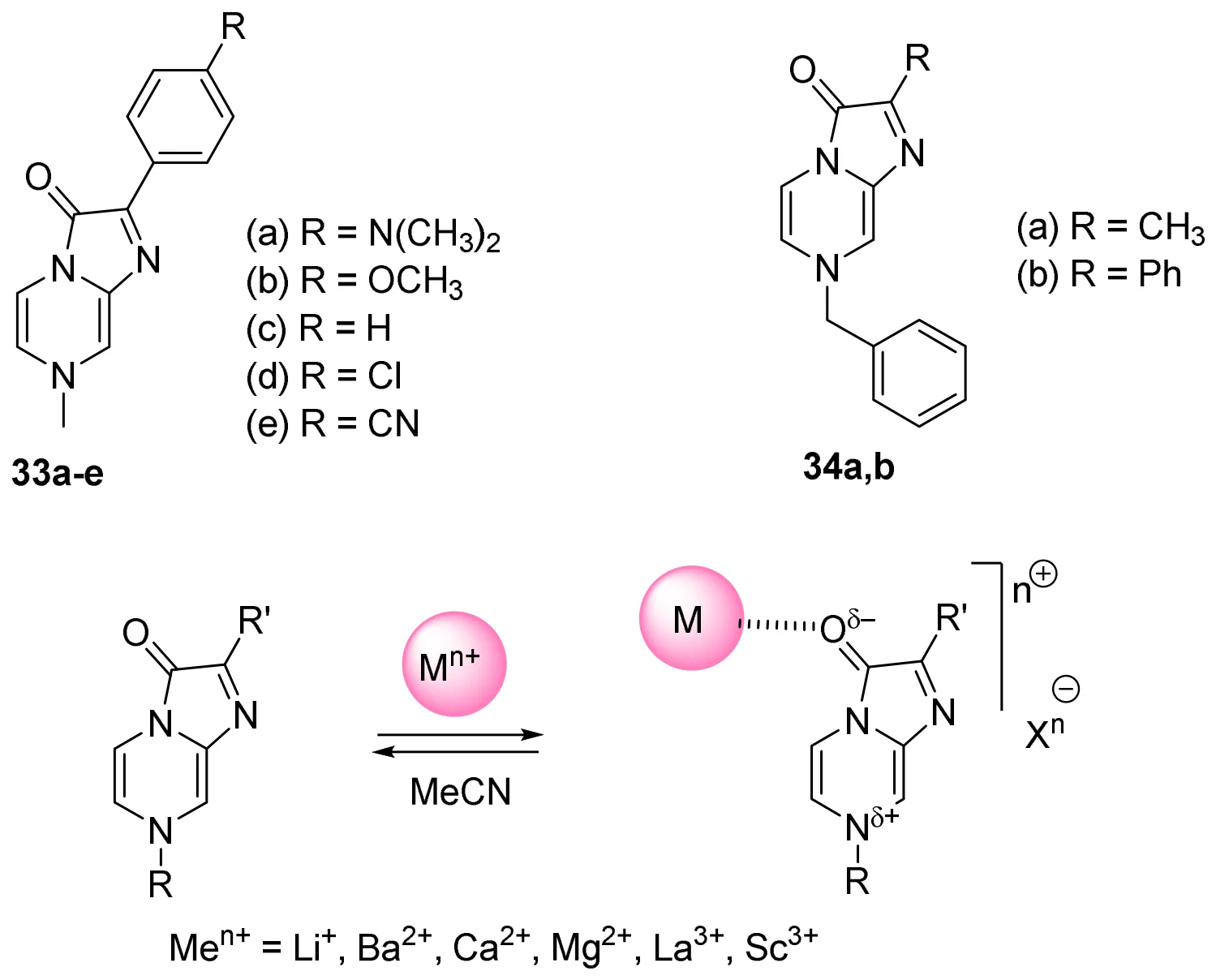
Molecular structures of imidazo[1,2-*a*]pyrazin-3(7*H*)-one sensors **33a**–**e** and **34a,b** and schematic representation of their interaction with various metal cations upon addition of perchlorate or trifluoromethanesulfonate salts (X^n−^).

Takamuki et al. systematically investigated another imidazo-annelated pyrazine scaffold, namely, imidazo[1,2-*a*]pyrazin-3(7*H*)-ones **33a**–**e** and **34a,b** (Figure 29), correlating their structure with photophysical properties [91,92]. Metal-ion complexation induces characteristic colorimetric changes in the cyano-substituted derivative **33e**, supporting its use as a promising colorimetric sensor for Lewis-acidic metal ions [91]. In continuation of the work derivatives of 7-benzylimidazo[1,2-*a*]pyrazin-3(7*H*)-one **34a,b** act as colorimetric and fluorometric sensors for Lewis-acidic metal ions in aprotic media (e.g., MeCN). Sensing arises from coordination of the metal ion (M**^n^**^+^) to the carbonyl oxygen atom of the imidazopyrazinone core (Figure 29), which is proposed to stabilize imidazopyrazinium resonance form [92]. This interaction causes a blue shift in the absorption and emission bands and increases the fluorescence quantum yield. Mechanistically, coordination of Lewis-acidic metal ions to the carbonyl oxygen stabilizes the imidazopyrazinium resonance form, causing a blue shift in the absorption and emission bands and an increase in the fluorescence quantum yield. The magnitude of the spectral shift and intensity enhancement correlates with the Fukuzumi Lewis acidity parameter (ΔE): higher metal-ion acidity produces larger spectral changes. Accordingly, compounds **34a,b** enable quantitative determination of Li^+^, Mg^2+^, Ca^2+^, Ba^2+^, Sc^3+^ and La^3+^ based on their Lewis acidity in oxygen-free media [92].

**Figure 30:**
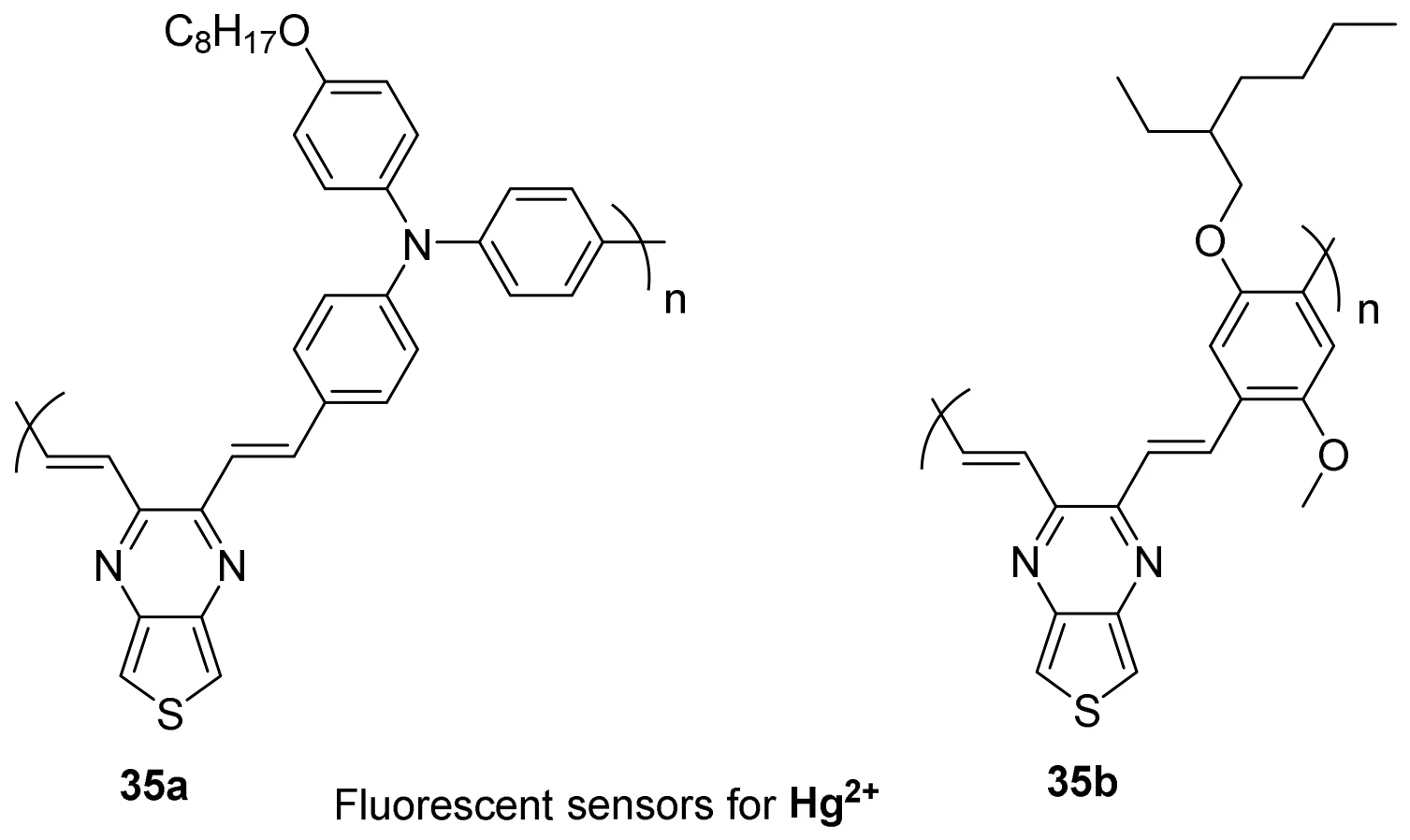
Molecular structure of the D-π-A copolymers **35a** and **35b**.

Two D–π–A conjugated polymers, **35a** and **35b**, incorporating an electron-accepting thieno[3,4-*b*]pyrazine unit in the backbone and either a triphenylamine or phenylene donor (Figure 30), have been reported [93].

In THF, both polymers are emissive: **35a** shows red emission at 644 nm, whereas **35b** emits yellow-green at 546 nm. Upon addition of Hg^2+^ the fluorescence of both materials is efficiently quenched, a process attributed to interaction of Hg^2+^ with the thienopyrazine segments that facilitates electron- or energy-transfer quenching pathways. At 300 ppm Hg^2+^ **35a** exhibits a 79% decrease in fluorescence intensity with a K_SV_ = 1.1 × 10^4^ M^−1^, while **35b** shows 60% quenching and two-fold higher K_SV_ of 2.1 × 10^4^ M^−1^. Hg^2+^ quenches the emission of **35a,b** through electron- or energy-transfer pathways involving the thienopyrazine segments, with the more electron-rich triphenylamine donor in 35a enhancing the sensitivity. Both polymers display pronounced selectivity for Hg^2+^ over a range of competing cations (Al^3+^, Ba^2+^, Cd^2+^, Co^2+^, Cu^2+^, Ni^2+^, K^+^, Mg^2+^, Mn^2+^, Pb^2+^ and Na^+^), with Hg^2+^ quenching efficiencies 2–7 times greater than those of other ions. Moreover, both polymers are soluble in common organic solvents and thermally stable, with 5% mass-loss temperatures above 360 °C for **35a** and above 240 °C for **35b**. On this basis, derivative **35a** appears to be the more promising candidate due to its superior properties, including higher thermal stability. The enhanced sensitivity and selectivity of **35a** are proposed to arise from the stronger electron-donating character of the TPA unit. Overall, these results indicate that thienopyrazine-containing conjugated polymers are promising platforms for fluorescent chemosensors for Hg^2+^ ions.

Annelation of the pyrazine core with five-membered carbo- or heterocycles represents a powerful strategy for tuning the photophysical properties and expanding the sensing capabilities of pyrazine-based chemosensors. The extension of the π-conjugated system typically shifts the absorption and emission maxima to longer wavelengths, which is advantageous for biological imaging applications. The reported examples include sensors for Hg^2+^, Pd^2+^, and Lewis-acidic metal ions, with detection mechanisms ranging from CHEQ to colorimetric changes based on Lewis acidity. Notably, the imidazopyrazinone derivatives **33** and **34** represent a unique approach to metal-ion sensing based on the stabilization of resonance forms upon coordination, enabling quantitative determination of Li^+^, Mg^2+^, Ca^2+^, and other ions. However, the number of studies in this area remains limited, and the full potential of annelated pyrazines, particularly those incorporating AIE-active or photochromic units, has yet to be systematically explored.

### 3.2. Quinoxalines

Among annelated pyrazine derivatives, a special place is occupied by quinoxalines—benzoannulated pyrazines—which, due to their electron-deficient aromatic framework, high photostability, and convenient structural tunability, are widely used for the design of chromogenic and fluorogenic chemosensors. In a recent comprehensive review, Kayogolo et al. [10] summarized the literature from 2018 to 2024 on the application of quinoxaline derivatives for the detection of cations, anions, and neutral molecules, including recognition mechanisms, detection limits, and practical aspects of their use. Earlier related work also surveyed quinoxaline derivatives as supramolecular building blocks for metal cation detection, providing specific solution examples, optical interaction mechanisms, detection limits, and solvent effects [94]. In this chapter of the manuscript, we present data on new quinoxaline-based sensors for metal ions published after the review by Kayogolo and co-workers [10].

Quinoxaline-based sensors operate through coordination-induced hyperchromic effects, static quenching, multi-mechanistic CHEF, or ICT, with several examples validated in real water samples and biological fluids. The additional benzene ring fused to the pyrazine core enhances the electron-deficient character of the heterocycle, extends the π-conjugated system, and provides a convenient handle for further functionalization at the 2- and 3-positions. As a result, quinoxaline derivatives are proposed to display a broader mechanistic diversity than their non-annulated counterparts, including ESIPT blocking, PET suppression, and C=N isomerization rigidification within a single molecular scaffold.

**Figure 31:**
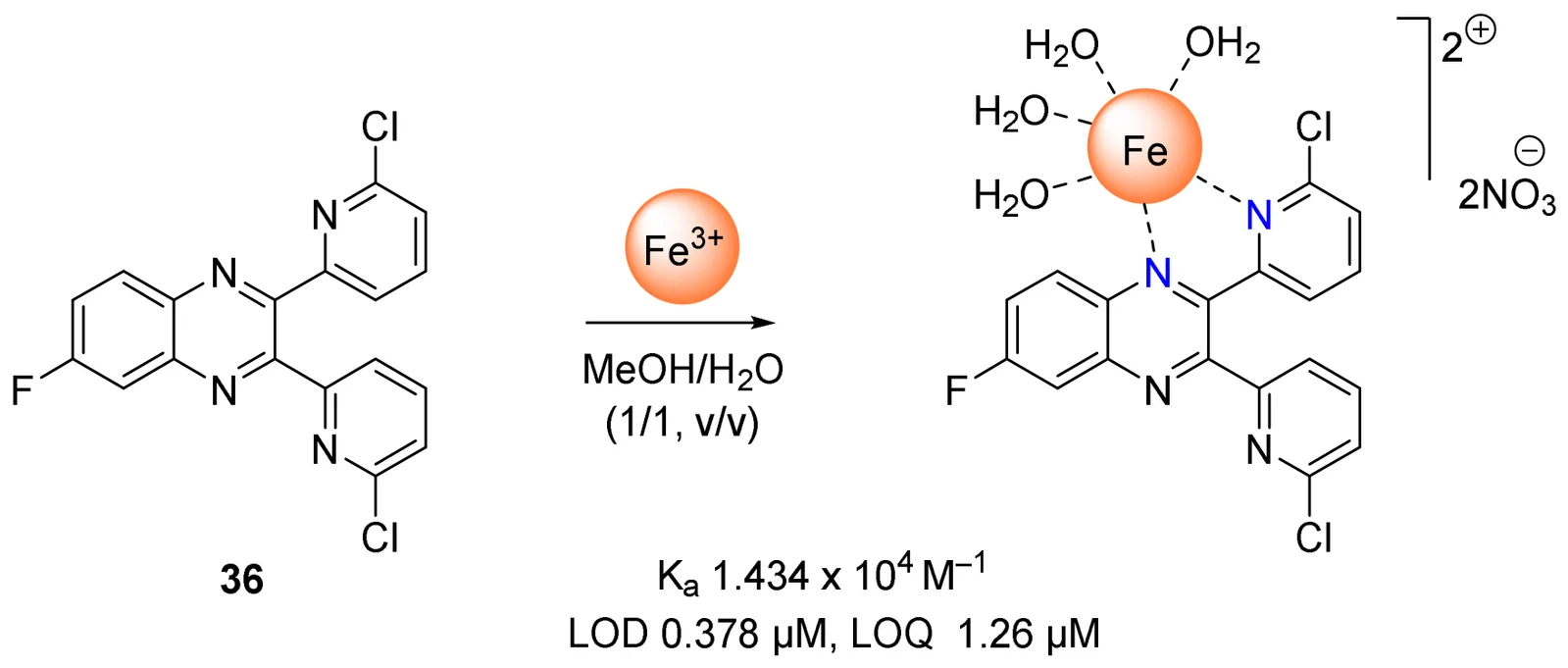
Molecular structure of 2,3-bis(6-chloropyridin-2-yl)-6-fluoroquinoxaline **36** and its binding mode with Fe^3+^ upon addition of FeCl_3_ × 6H_2_O. Color coding: nitrogen atoms involved in metal coordination are shown in blue.

2,3-Bis(6-chloropyridin-2-yl)-6-fluoroquinoxaline **36** (Figure 31), prepared by condensation of 4-fluorobenzene-1,2-diamine with 1,2-bis(6-chloropyridin-2-yl)ethane-1,2-dione, detects Fe^3+^ via coordination to the quinoxaline core and pyridine nitrogen atoms [95]. Binding induces a visible color change and a hyperchromic response in the UV–vis spectrum, whereas other tested metal cations do not affect the absorption band. Coordination of Fe^3+^ to the quinoxaline and pyridine nitrogen atoms produces a hyperchromic response in the UV-vis spectrum, without a pronounced fluorescence “turn-on”; the response is therefore best described as purely colorimetric.

Job’s plot and molar ratio analysis (absorbance at 280 nm versus [Fe^3+^]) indicate a 1:1 stoichiometry between probe **36** and Fe^3+^. FT-IR measurements support the proposed binding mode and stoichiometry (Figure 31). The association constant K_a_ was estimated as 1.434 × 10^4^ M^−1^. The LOD and LOQ are 0.378 µM and 1.26 µM, respectively. Applicability to environmental samples was confirmed by analysis of tap water, which gave accurate recoveries with relative errors < 3.93%, demonstrating the probe’s suitability for detecting Fe^3+^ in environmental samples. Moreover, the complex **36**-Fe^3+^ remained stable at physiological pH = 7.4, indicating potential probe **36** for biological applications [95].

**Figure 32:**
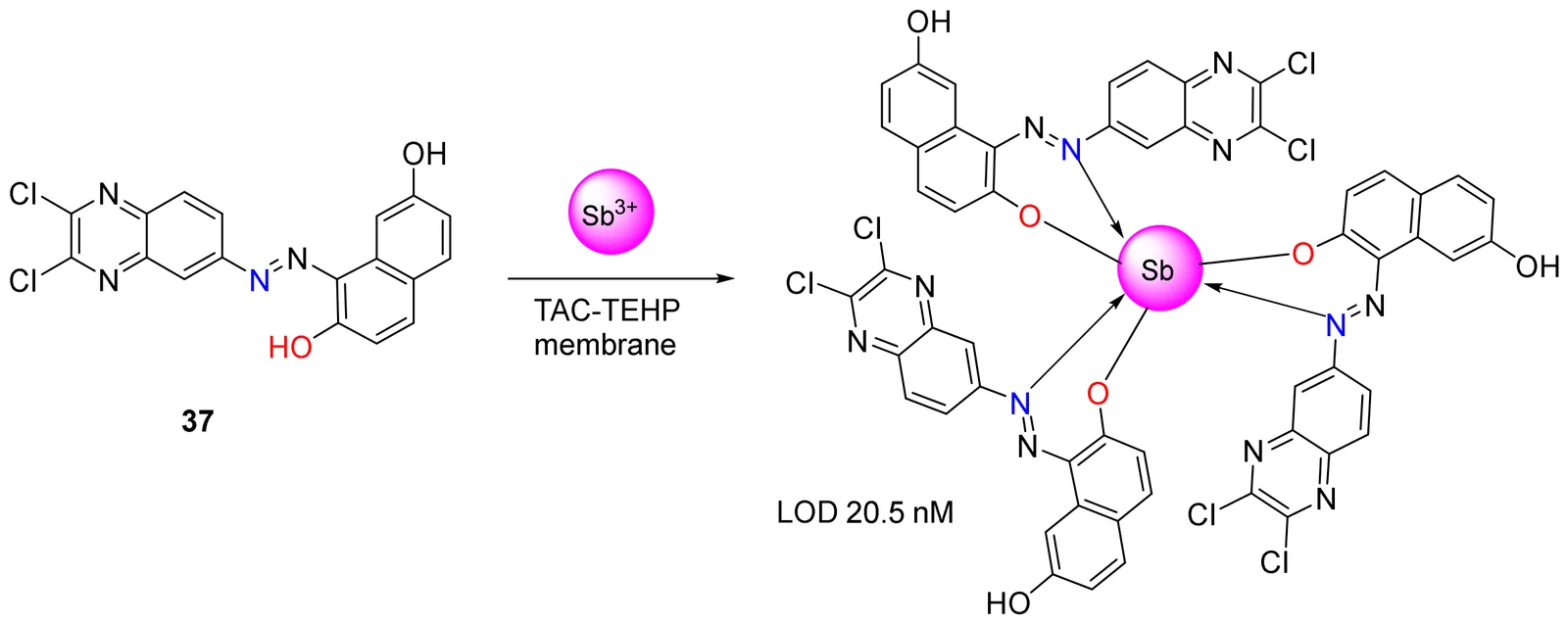
Molecular structure of 2,3-dichloro-6-(2,7-dihydroxynaphth-1-ylazo)quinoxaline **37** and its binding mode with Sb^3+^ upon addition of potassium antimony tartrate. Color coding: nitrogen atoms involved in metal coordination are shown in blue; oxygen atoms involved in metal coordination are shown in red.

Al Zbedy and co-workers [96] developed a highly selective and regenerative optical sensor (optode) for Sb^3+^ based on a triacetylcellulose membrane incorporating 2,3-dichloro-6-(2,7-dihydroxynaphth-1-ylazo)quinoxaline **37** (Figure 32) as the chromogenic ionophore and tri-(2-ethylhexyl)phosphate (TEHP) as a plasticizer. The sensing principle relies on the formation of a colored **37**-Sb^3+^ complex (3:1 stoichiometry) within the polymer matrix, giving rise to a distinct absorbance band at 665 nm. The optode operates through formation of a colored **37**–Sb^3+^ complex (3:1 stoichiometry) within the polymer matrix, i.e., via coordination-induced colorimetric complexation rather than through a fluorescent mechanism.

The optode **37** exhibited a linear response in the range of 8.0–144 ng mL^−1^, with LOD of 2.5 ng mL^−1^ (ca. 20.5 nM for Sb^3+^) and a response time of 4–5 min. The sensor demonstrated excellent selectivity over a wide range of interfering ions, complete regeneration within 2 min using 0.1 M HCl, and good durability over multiple cycles. The method was successfully validated for total antimony determination (following reduction in Sb^5+^ to Sb^3+^ with ascorbic acid and KI) in water, biological (blood, urine, kidney, femur), and food samples, highlighting its practical utility for environmental and biomedical monitoring.

**Figure 33:**
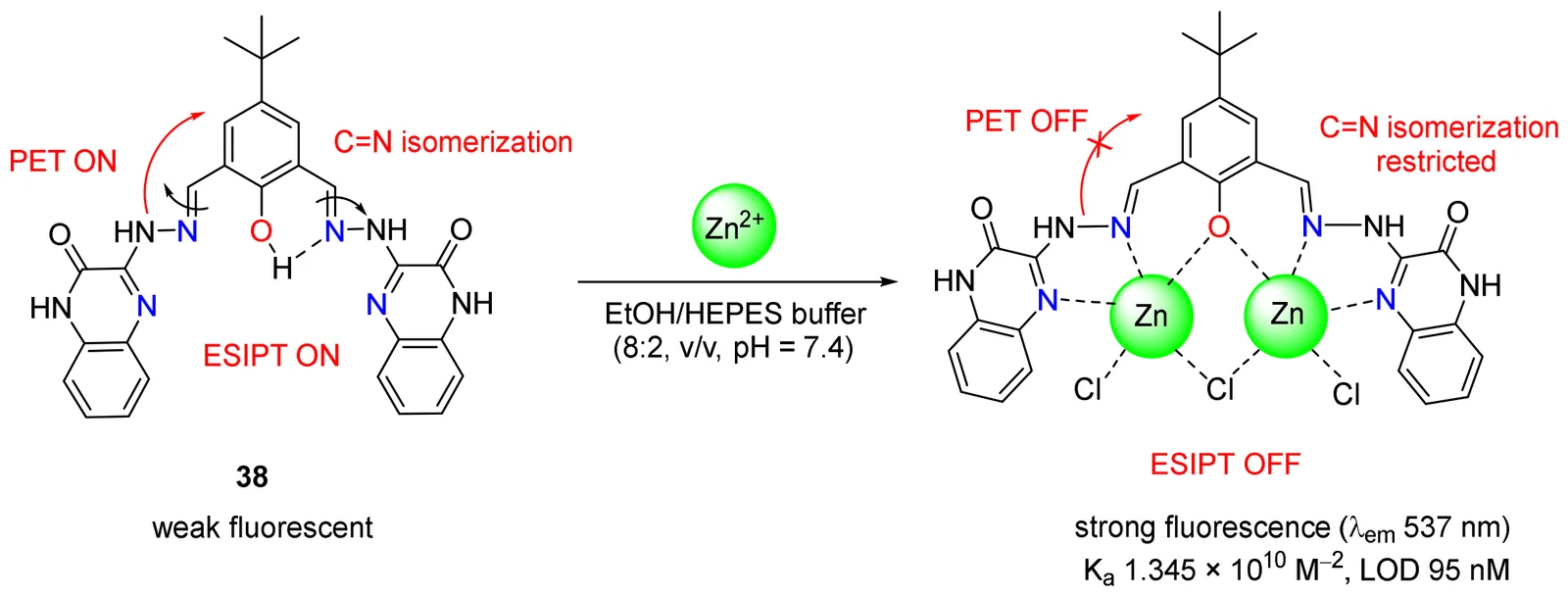
Molecular structure of sensor **38** and its binding mode with ZnCl_2_. Color coding: nitrogen atoms involved in metal coordination are shown in blue; oxygen atom involved in metal coordination is shown in red; the proposed detection mechanisms are shown in red.

Kumar et al. [97] developed a polydentate hydrazone ligand **38** (Figure 33), synthesized from 4-*tert*-butyl-2,6-diformylphenol and 3-hydrazineylquinoxalin-2(1*H*)-one, as a highly selective “turn-on” fluorescent sensor for Zn^2+^ ions over a broad range of competing metal ions, including Cd^2+^ and Hg^2+^. In its free state, **38** exhibits weak emission due to the synergistic operation of three non-radiative pathways: ESIPT, PET, and C=N isomerization. Upon coordination with Zn^2+^ in a 1:2 (**38**:Zn^2+^) stoichiometry, the phenolic proton is displaced; Zn^2+^ binding simultaneously blocks ESIPT, suppresses PET, restricts C=N isomerization, and activates CHEF, producing a dramatic “turn-on” response; the mechanism is therefore best described as multi-mechanistic CHEF (Figure 33). This multi-mechanistic inhibition results in a dramatic fluorescence enhancement at 537 nm, accompanied by a distinct yellowish-green color of solution visible to the naked eye. The sensor **38** operates effectively in the biologically compatible pH range of 6–12, exhibits reversible binding with EDTA, and achieves a detection limit of 95 nM, making it suitable for Zn^2+^ monitoring in real water samples.

The 2:1 binding stoichiometry and coordination mode were unequivocally confirmed by single-crystal X-ray diffraction of the isolated complex [Zn_2_(**38**)Cl_3_]·2H_2_O·2.5DMF, in which ligand **38** adopts a monoanionic pentadentate bridging coordination geometry (Figure 33). DFT and TD-DFT calculations indicate that formation of the [Zn_2_(**38**)Cl_3_] complex is proposed to stabilize the frontier orbitals and is consistent with a reduction in the HOMO-LUMO gap of ligand **38**, in agreement with the experimentally observed slight bathochromic shift in the absorption maximum of **38** upon coordination of Zn^2+^.

**Figure 34:**
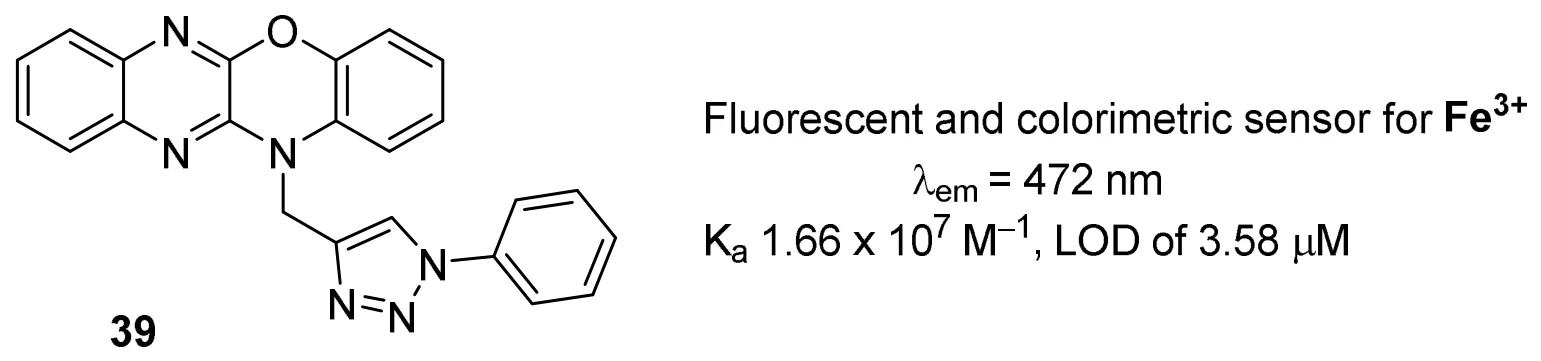
Quinoxaline–triazole conjugate **39** as a selective turn-off fluorescent and colorimetric sensor for Fe^3+^ in methanol.

A quinoxaline–triazole conjugate **39** (Figure 34), developed by Narayan et al. [98] acts as a selective “turn-off” fluorescent and colorimetric sensor for Fe^3+^ ions in methanol. The probe **39** exhibits a single absorption band at 378–391 nm (π→π* transition) and emission at ~472 nm. Upon interaction with Fe^3+^, a hypsochromic shift in the absorption spectrum is observed and the fluorescence is completely quenched, while no significant response is detected for other competing metal ions (Co^2+^, Na^+^, Ni^2+^, Zn^2+^, Cu^2+^, Cr^3+^, Mg^2+^, Hg^2+^, Al^3+^, Fe^2+^, Ce^3+^, K^+^) or anions (F^−^, Cl^−^, Br^−^, I^−^, CN^−^). Job’s plot analysis confirmed a 1:1 binding stoichiometry, with a binding constant of 1.66 × 10^7^ M^−1^. However, elucidation of the detailed binding mode by a combination of methods (ESI-MS, ^1^H NMR titration, and FT-IR) is challenging due to the paramagnetic nature of the Fe^3+^ ion and the absence of well-defined coordination-induced shifts in the FT-IR spectrum.

The quenching mechanism of **39** toward Fe^3+^ was established as static, originating from ground-state complexation, as evidenced by temperature-dependent Stern–Volmer studies and fluorescence lifetime measurements (1.499 ns for **39** vs. 1.503 ns for the **39**-Fe^3+^ complex) [98]. Temperature-dependent Stern–Volmer studies and fluorescence lifetime measurements support a static quenching mechanism originating from ground-state complexation, rather than from dynamic collisional quenching.

The sensor **39** exhibits a rapid response time (5 s), operates effectively over a broad pH range (2–12), and demonstrates excellent thermal and photostability. The LOD for Fe^3+^ was determined to be 3.58 μM, which is below the WHO (5.0 µM) and US EPA (5.4 µM) guidelines for iron in drinking water. Reversibility was confirmed using EDTA, enabling sequential “turn-off–on–off” cycles. The practical utility of **39** was validated in real water samples (river, tap, groundwater, and distilled water), with recovery rates ranging from 71% to 91%.

**Figure 35:**
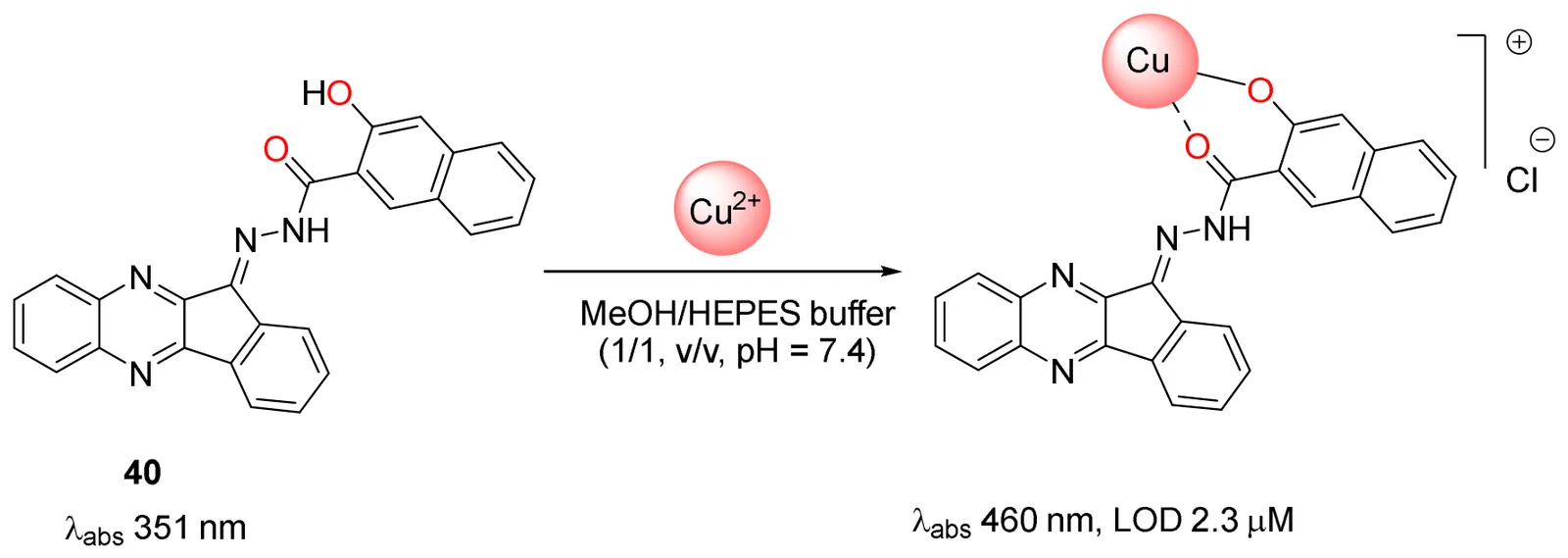
Molecular structure of sensor **40** and its binding mode with Cu^2+^ upon addition of CuCl_2_. Color coding: oxygen atoms involved in metal coordination are shown in red.

Another representative conjugated quinaxoline derivative **40** (Figure 35), proposed by Mondal and Manivannan, functions as a colorimetric chemosensor toward Cu^2+^ [99]. Compound **40** was synthesized by reacting 11*H*-indeno[1,2-*b*]quinoxalin-11-one with 3-hydroxy-2-naphthoic hydrazide in ethanol, forming an O,O-coordination center for specific Cu^2+^ binding. Involvement of the carbonyl-O and phenolate-O atoms in coordination with Cu^2+^ (Figure 35) was confirmed by IR and NMR experiments.

The sensory signal manifests as yellow coloring its MeOH/HEPES buffer solution and the appearance of red-shifted absorption band from 351 to 460 nm; the signal is not disturbed by other competing metal cations, providing selectivity of the described sensor **40**. Coordination of Cu^2+^ through the carbonyl and phenolate oxygen atoms is proposed to induce an ICT transition, shifting the absorption maximum from 351 to 460 nm; the response is purely colorimetric. From the titration of **40** with CuCl_2_, the LOD was determined to be 2.3 µM, which is lower than the permissible limit in drinking water specified by the WHO. The LOQ value was 8.0 µM, and the binding constant was determined to be 1.13 × 10^4^ M^−1^. Other sensing parameters were determined as follows: pH range of 5–10, response time of 50 s, and reversibility of coordination–dissociation process.

Notably, the **40**-Cu^2+^ complex formed in situ is sensitive toward cysteine and ATP. The interaction mechanism between **40**-Cu^2+^ and cysteine involves reduction of Cu^2+^ to Cu^0^, accompanied by the release of free **40** and cystine. Recognition of ATP is based on displacement of the ligand **40** by ATP due to a higher binding constant value (8.9 × 10^4^ M^−1^ for ATP-Cu^2+^ vs. 1.13 × 10^4^ M^− 1^ for **40**-Cu^2+^). The color and spectrum of both solutions return to those of pure **40**.

Chemosensor **40** demonstrated practical, rapid, and naked-eye detection of Cu^2+^ ions in real water samples as well as on paper strips, affording slightly higher detection limits of 5.01–5.5 μM and 3.39 µM, respectively, compared to laboratory results [99].

**Figure 36:**
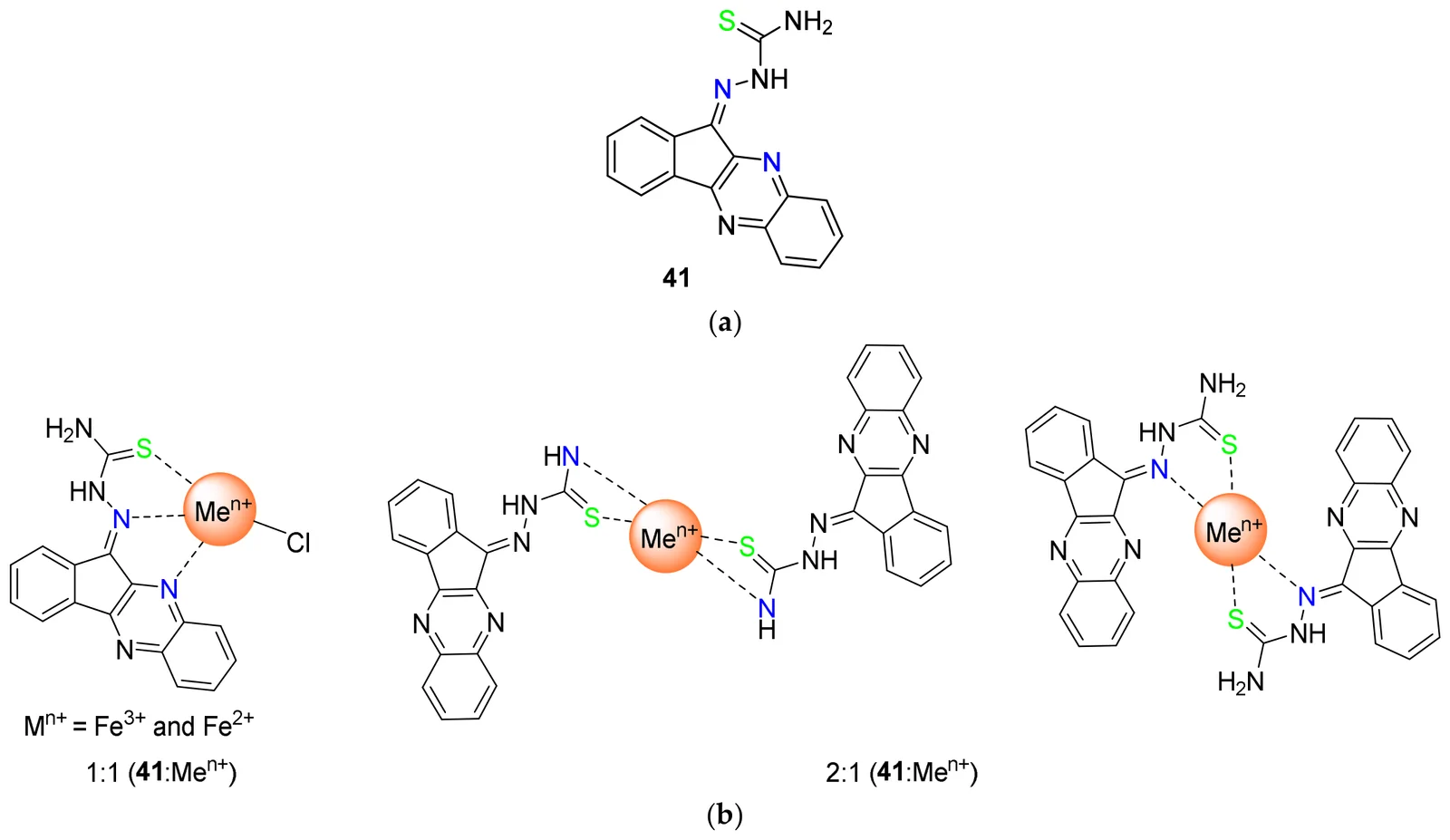
(**a**) Molecular structure of quinoxaline–thiosemicarbazide conjugate **41**; (**b**) proposed binding modes of **41** with metal cations in DMSO/H_2_O (8/2, *v*/*v*) upon addition of the corresponding nitrate or chloride salt. Color coding: nitrogen atoms involved in metal coordination are shown in blue; sulfur atom involved in metal coordination is shown in green.

Functionalization of 11*H*-indeno[1,2-*b*]quinoxalin-11-one with thiosemicarbazide in MeOH produced polycyclic chemosensor **41** (Figure 36a), which functions as a chromogenic naked-eye chemosensor capable of distinguishing ten different transition metal ions (Fe^3+^, Fe^2+^, Co^2+^, Ni^2+^, Cu^2+^, Zn^2+^, Pb^2+^, Ag^+^, Cd^2+^, and Hg^2+^) in aqueous DMSO solution through distinct colorimetric responses [100]. The sensing mechanism relies on multiple coordination sites within the thiosemicarbazide moiety, including the azomethine nitrogen, thiocarbonyl sulfur, and the acidic N–H proton. Upon interaction with metal ions, the complex **41**-Me^n+^ produces significant bathochromic shifts (up to 470 nm for Cu^2+^) and visible color changes ranging from yellow to black, orange, or brown. Multi-site coordination through the azomethine nitrogen, thiocarbonyl sulfur, and the acidic N–H proton is proposed to produce distinct ICT-based colorimetric responses for ten transition metal ions, allowing naked-eye discrimination within a single sensor platform. Job’s plot analysis revealed a 1:1 stoichiometry for Fe^3+^/Fe^2+^ with binding constants of 2.31–1.22 × 10^3^ M^−1^, respectively, and a 2:1 (ligand:metal) stoichiometry for other metals, with K_a_ values, ranging from 8.70 × 10^8^ to 1.36 × 10^11^ M^−2^ exhibiting the high affinity. The proposed binding structures are summarized in Figure 36b.

The sensor achieved LODs in the range of 3.94–0.08 μM, with the lowest value for Cd^2+^, and the practical utility of **41** was demonstrated through paper-strip-based visual detection, offering a simple, rapid, and cost-effective platform for multi-analyte screening in environmental and analytical applications. Notably, the same structure **41** was shown to be inert toward different metal cations in MeCN and to selectively recognize the F^−^ in MeCN [101], highlighting the influence of the medium on the recognition properties of **41**.

So, quinoxalines, as benzo-annulated analogs of pyrazine, have emerged as a mature and extensively explored class of chemosensors, as comprehensively reviewed by Kayogolo and co-workers. The additional benzene ring enhances the electron-deficient character of the pyrazine core and provides a convenient handle for further functionalization. The sensors discussed in this section, published after the aforementioned review, demonstrate the continued evolution of quinoxaline-based probes: from simple colorimetric sensors for Fe^3+^ and Cu^2+^ (**36**, **40**) to sophisticated systems capable of distinguishing multiple transition metal ions through distinct colorimetric responses (**41**), and turn-on fluorescent probes for Zn^2+^ (**38**) with crystallographically confirmed binding modes. The successful application of these sensors in real water samples, biological fluids, and on paper strips supports their practical relevance. However, the selectivity of quinoxaline-based sensors remains a recurring challenge, as many probes respond to multiple metal ions, and the development of truly orthogonal sensing systems for complex matrices is still an open challenge.

### 3.3. Pyridopyrazines

Due to the additional pyridine nitrogen atoms in the fused pyrido[2,3-*b*]pyrazine ring, it has a greater electron-accepting ability than quinoxaline, despite their similar heterocyclic frameworks. Pyrido[2,3-*b*]pyrazines display the most diverse mechanisms among the annulated systems discussed in this review, including AIEE, PET/energy-transfer quenching, photochromic switching, and thermally activated delayed fluorescence (TADF)/ICT modulation, reflecting their enhanced electron-accepting character. The additional pyridine nitrogen atom in the fused ring is thought to increase the electron deficiency in the scaffold relative to quinoxaline and may provide an extra coordination site, making these compounds particularly attractive for the detection of soft and borderline metal ions such as Hg^2+^, Sn^2+^, and Zn^2+^. Their extended conjugation also shifts emission into the orange-to-red region, which is advantageous for biological imaging and for the design of ratiometric sensors.

**Figure 37:**
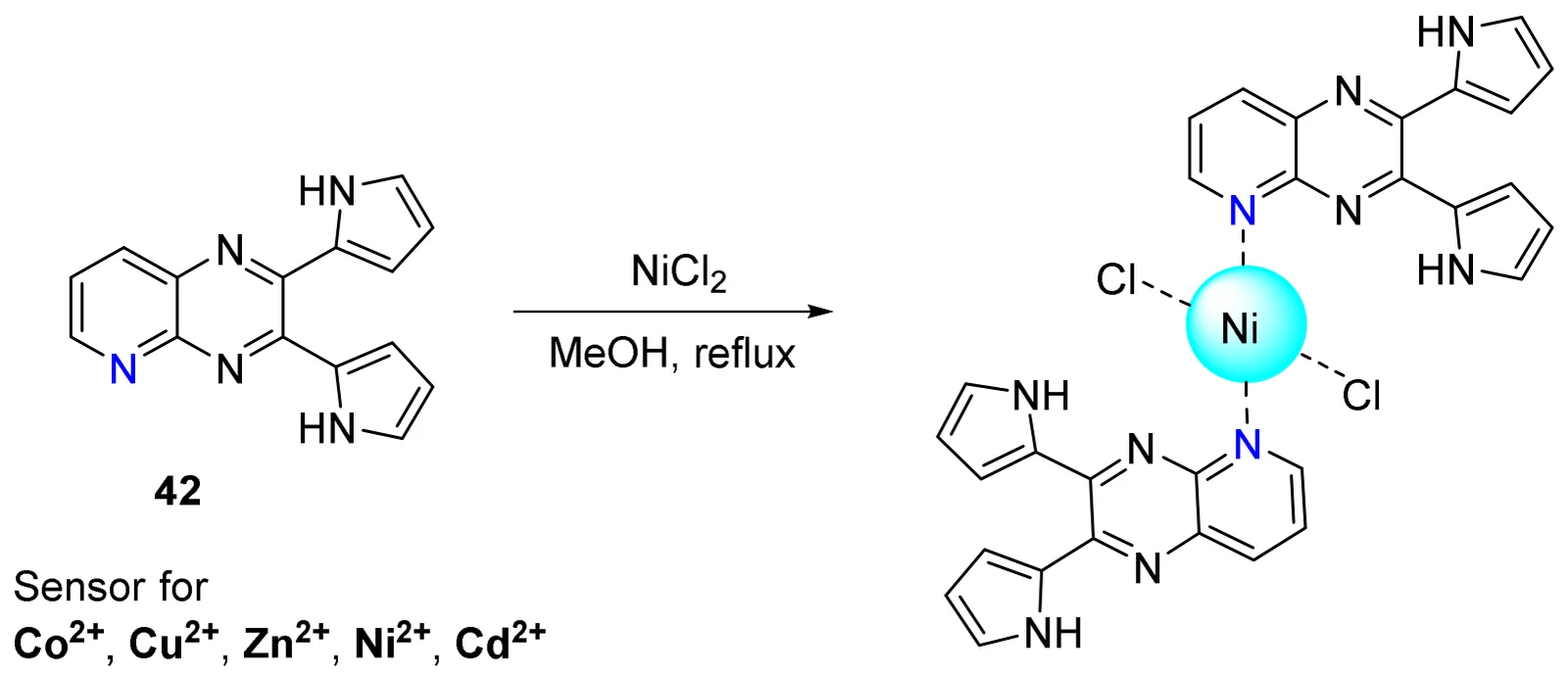
Molecular structure of 2,3-di(1*H*-2-pyrrolyl)pyrido[2,3-*b*]pyrazine **42** and its binding mode with NiCl_2_. Color coding: nitrogen atom involved in metal coordination is shown in blue.

2,3-Di(1*H*-2-pyrrolyl)pyrido[2,3-*b*]pyrazine **42** (Figure 37) exhibits pronounced changes in both its absorption spectrum and visible color upon addition of several metal cations (Co^2+^, Cu^2+^, Zn^2+^, Ni^2+^ and Cd^2+^) in acetonitrile. Upon titration emission spectrum of **42**-Me^2+^ appears with λ_max_ at 496 nm, while the presence of an isosbestic point indicates an equilibrium between **42** and the **42**-Me^2+^ complex during titration [102]. Coordination of transition metals to the pyridine and pyrrole nitrogen atoms generates a new emission band at 496 nm; the mechanism resembles CHEF, although the contribution of conformational rigidification has not been quantified. The determined association constants (K_a_ = 0.68 × 10^5^–15 × 10^5^ M^−1^) reveal varying affinities for transition metals, following the order Cd^2+^ < Co^2+^ < Zn^2+^ < Ni^2+^ < Cu^2+^. Complexation mode include pyridine nitrogen atom that showing its essential role in detecting properties. The pyridine nitrogen is involved in coordination and is essential for sensing behavior. The proposed binding mode was supported by the X-ray diffraction of the **42**-Ni^2+^ complex, obtained by refluxing **36** with NiCl_2_ (2:1 equiv.) in MeOH: the Ni^2+^ center is coordinated by two **42** ligands and two chloride ions in a distorted tetrahedral geometry [102].

**Figure 38:**
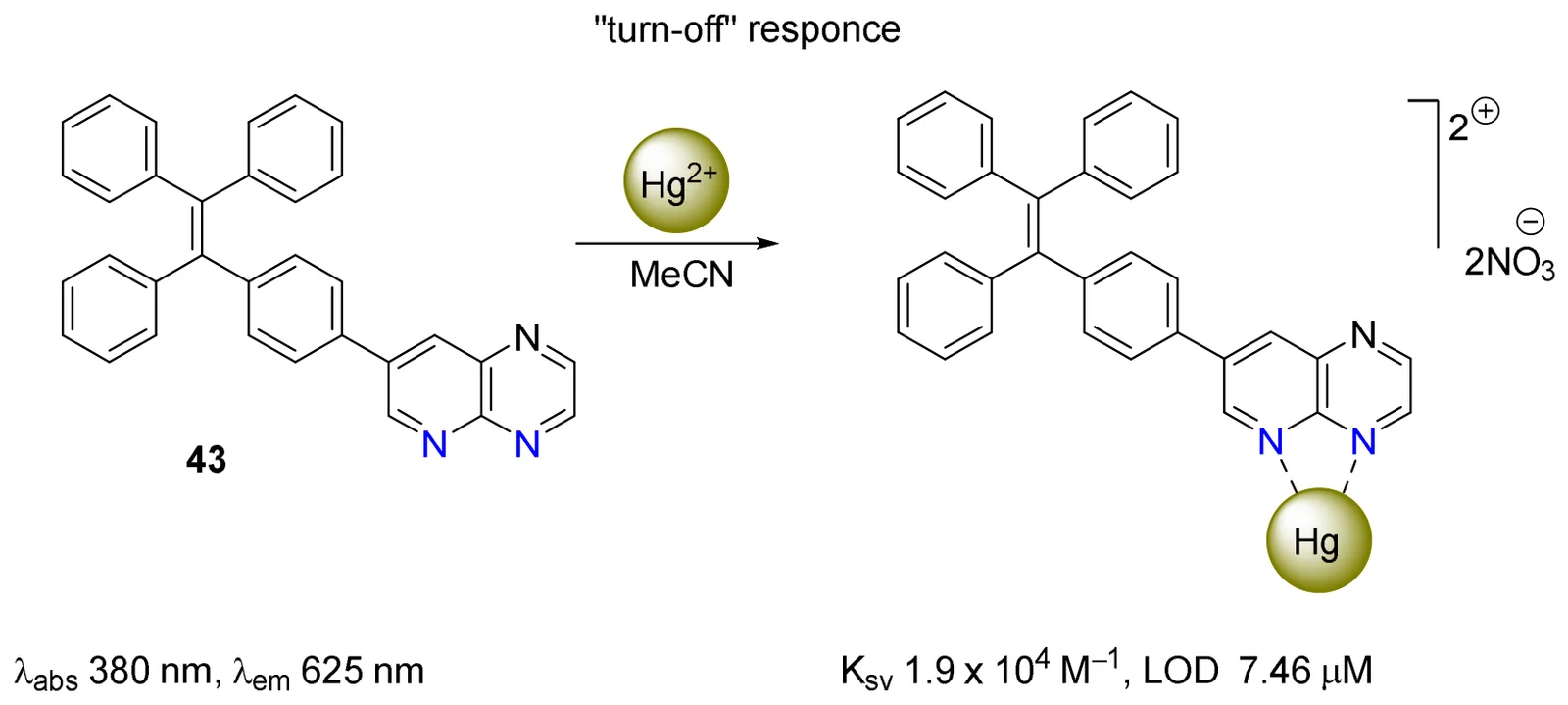
Molecular structure of tetraphenylethene-substituted pyrido[2,3-*b*]pyrazine **43** and its binding mode with Hg^2+^ upon addition of Hg(NO_3_)_2_. Color coding: nitrogen atoms involved in metal coordination are shown in blue.

**Figure 39:**
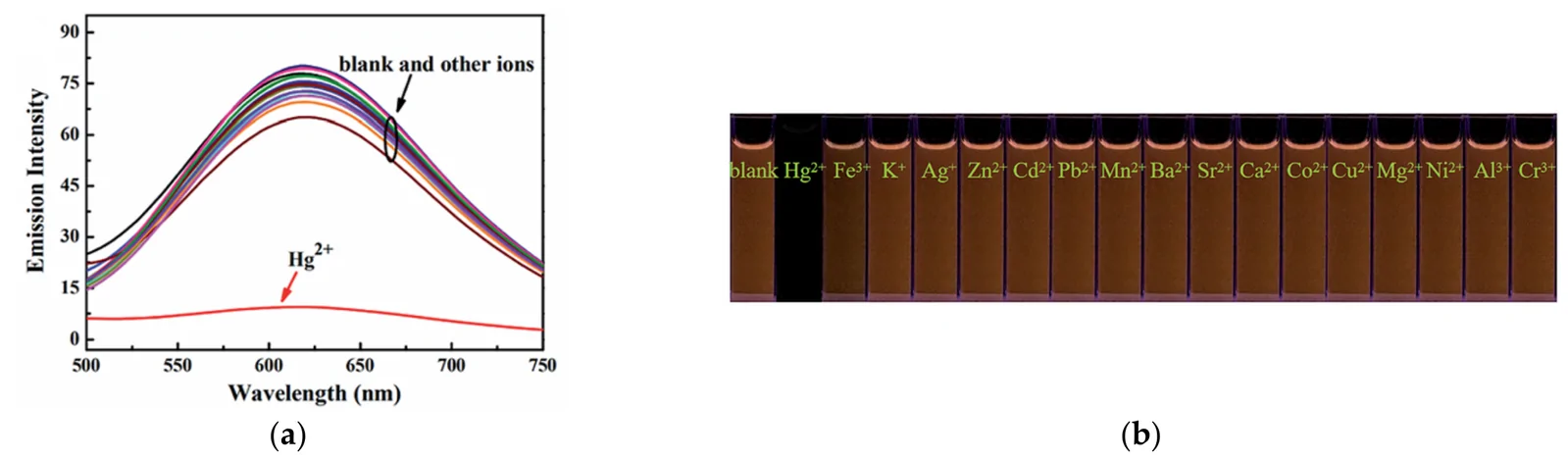
(**a**) Fluorescence spectra of sensor **43** (2.0 × 10^−5^ mol L^−1^) towards various cations, excitation wavelength 380 nm (red line is spectrum of **43** upon addition of Hg^2+^, the other lines correspond to the free sensor and to its spectra upon addition of Fe^3+^, K^+^, Ag^+^, Zn^2+^, Cd^2+^, Pb^2+^, Mn^2+^, Ba^2+^, Sr^2+^, Ca^2+^, Co^2+^, Cu^2+^, Mg^2+^, Ni^2+^, Fe^3+^, Cr^2+^); (**b**) Fluorescence photographs of sensor **43** after addition of various metal ions (7.0 equiv.) under 365 nm light. Reproduced with permission of Royal Society of Chemistry [103].

Tang et al. reported synthesis of 7-(4-(1,2,2-triphenylvinyl)phenyl) pyrido[2,3-*b*]pyrazine **43** (Figure 38) as multifunctional organic materials [103]. Fluorophore **43** combining a donor tetraphenylethene (TPE) fragment and an acceptor pyrido[2,3-*b*]pyrazine core, exhibits aggregation-induced emission enhancement (AIEE) in MeCN/H_2_O mixture at a water fraction of 90% due to restriction of intramolecular rotation (RIR), primarily of the TPE aryl groups. Moreover, compound **43** demonstrates solvatochromic behavior arising from an ICT effect and superior cell-imaging performance owing to its AIEE activity and low cytotoxicity. In addition, this compound can detect Hg^2+^ ions in acetonitrile, operating as a “turn-off” chemosensor (Figure 39), with a visual color change from orange-red to dark under UV irradiation, while other tested metals do not influence its fluorescence.

Upon coordination with Hg^2+^, the nitrogen atoms of the pyridine and pyrazine moieties participate in cooperative binding with the metal ion in a 1:1 stoichiometry, as confirmed by Job’s plot, ^1^H NMR, and mass spectrometry (the proposed binding mode of **43** with Hg^2+^ is illustrated in Figure 38). This coordination is proposed to facilitate photoinduced electron or energy transfer processes, which is consistent with to a dramatic fluorescence quenching at 625 nm (λ_ex_ = 380 nm) with a quenching constant of 1.9 × 10^4^ M^−1^. Hg^2+^ quenches the AIEE emission of **43** through PET/energy-transfer processes, with the pyridine and pyrazine nitrogen atoms acting as cooperative binding sites in a 1:1 stoichiometry.

Sensor **43** shows high selectivity for mercury with minimal interference from other environmentally relevant metal ions (Zn^2+^, Cd^2+^, Pb^2+^, Cu^2+^, Fe^3+^, Ag^+^, etc.), a detection limit of 7.46 µM and represents unique chemosensor combining AIEE behavior, solvatochromic properties, and favorable cell imaging characteristics [103].

**Scheme 7:**
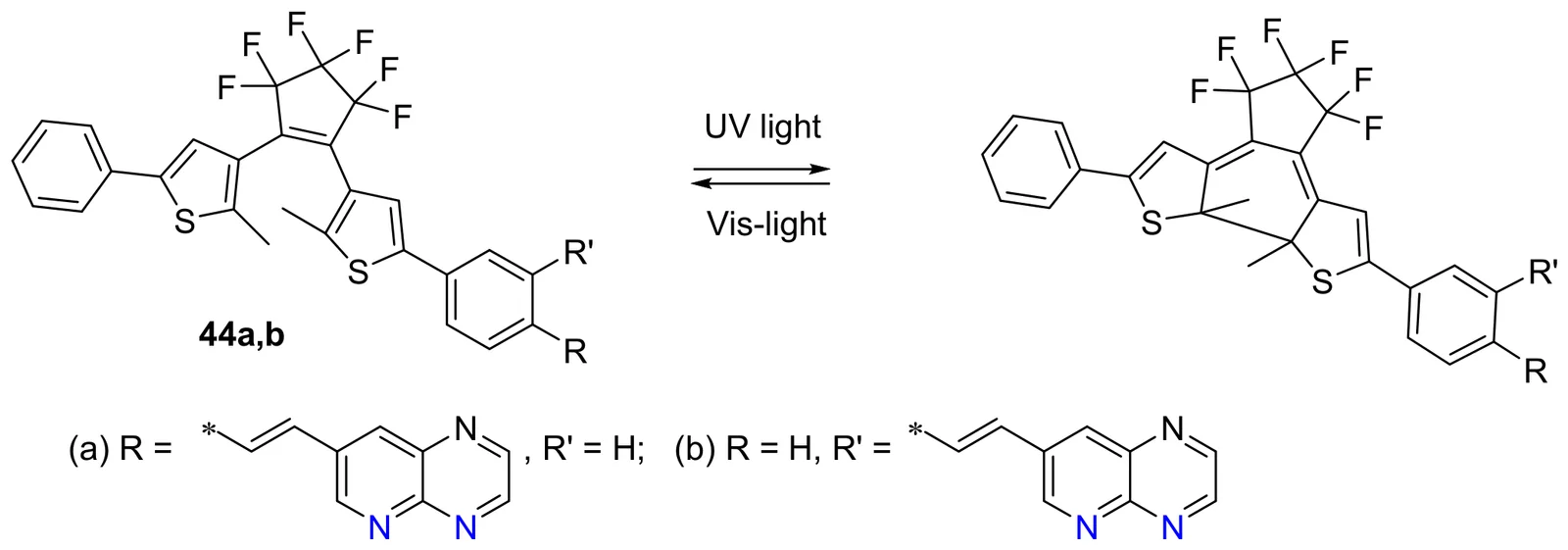
Photochromism of diarylethenes **44a**,**b** with a styrene-linked pyrido[2,3-*b*]pyrazine unit. Color coding: nitrogen atoms involved in metal coordination are shown in blue.

Later, the same research group employed the pyrido[2,3-*b*]pyrazine unit for functionalization of a diarylethene fragment, obtaining two isomeric photochromic chemosensors **44a,b** (Scheme 7), which function as colorimetric and fluorescent sensors for the detection of Hg^2+^ and Sn^2+^ ions [104].

Upon addition of Hg^2+^, the solutions of **44a,b** turn yellow, and a new red-shifted absorption band appears at 403 nm (for **44a**) and at 390 (for **44b**) along with a clear isobestic point, indicating a two-species (**44** and **38**-Hg^2+^) conversion process. UV–Vis absorption spectra, absorbance ratios, and competitive tests with 16 metal cations revealed high selectivity of both **44a** and **44b** toward Hg^2+^, with more pronounced changed and a lower LOD obtained for the *para*-substituted diarylethene **44a** (LOD = 0.498 μM) compare to the *meta*-substituted counterpart **44b** (LOD = 1.56 μM). Fluorescence titration of **44a** with Hg^2+^ revealed quenching of its emission at 560 nm (λ_ex_ = 415 nm) and visible darkening of the solution. The association constant and LOD of Hg^2+^ by **44a** were calculated to be 4.15 × 10^3^ M^−1^ and 4.53 µM, respectively.

**Scheme 8:**
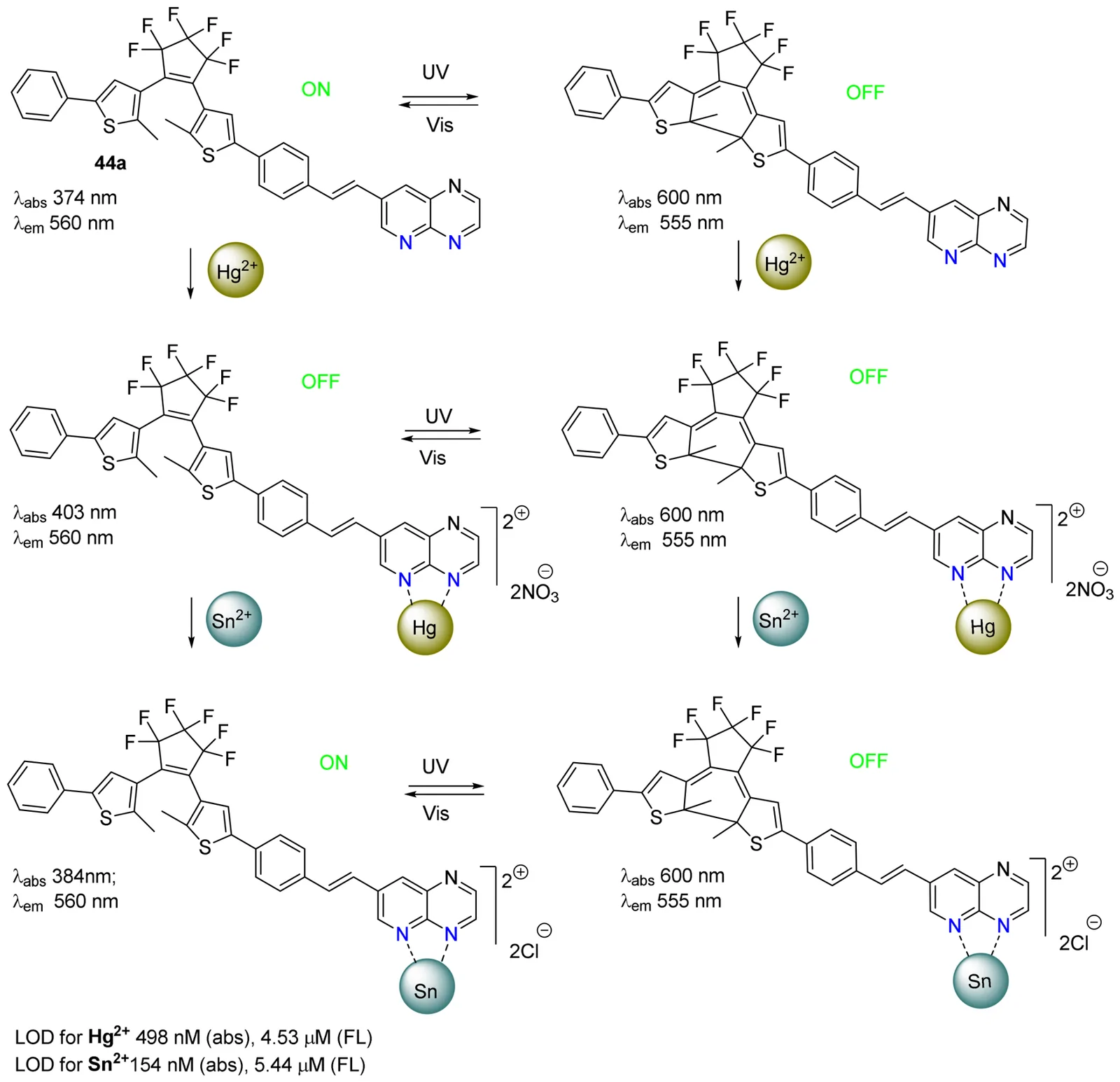
Dual-controlled fluorescent switching of photochromic diarylethene-pyrido[2,3-*b*]pyrazine derivative **44a** in response to Hg^2+^/Sn^2+^ and UV-vis stimuli upon addition of Hg(NO_3_)_2_ or SnCl_2_ in MeCN. Color coding: nitrogen atoms involved in metal coordination are shown in blue.

Interestingly, both **44a**-Hg^2+^ and **44b**-Hg^2+^ complexes can serve as a secondary platform for Sn^2+^ detection (Scheme 8). For example, upon addition of tin ions to **44a**-Hg^2+^, a hypsochromic shift in the absorption band from 403 to ~350 nm is observed, along with solution decolorization (from yellow to colorless) and partial fluorescence recovery (yellow emission, quantum yield 0.186). This behavior is attributed to displacement of Hg^2+^ from the coordination sphere and the formation of the **44a**-Sn^2+^ complex, in which diamagnetism property of Sn^2+^ is proposed to play a role in the “on” emission. The **44a,b**–Hg^2+^ complexes therefore operate as secondary platforms for Sn^2+^ via a displacement mechanism: Sn^2+^ displaces Hg^2+^ from the coordination sphere, restoring the fluorescence of the free sensor. The LODs are 154 nM and 36.4 nM for **44a**-Hg^2+^ and **44b**-Hg^2^, respectively (from absorption titration), and 5.44 µM in case of fluorescent titration of **44a**-Hg^2+^, demonstrating high sensitivity.

Results of UV/fluorescent and ^1^H NMR titration experiments revealed a 1:1 stoichiometry of **44** with Sn^2+^/Hg^2+^ with the most probable binding sites being the nitrogen atoms on the pyridine and the pyrazine rings (Scheme 8).

Notable reversible photoswitching was demonstrated for **44a**,**b** and their Me^2+^ complexes (Scheme 7 and Scheme 8): the compounds undergo photoinduced cyclization upon UV irradiation to form the closed-ring isomer and revert under visible light, allowing the optical properties to be modulated by external stimuli. Based on these properties, a combinational logic gate based on more sophisticated example **44a** was constructed with inputs (UV light, Hg^2+^ and Sn^2+^) and output (fluorescence intensity at 560 nm), demonstrating the potential for developing intelligent molecular devices.

**Figure 40:**
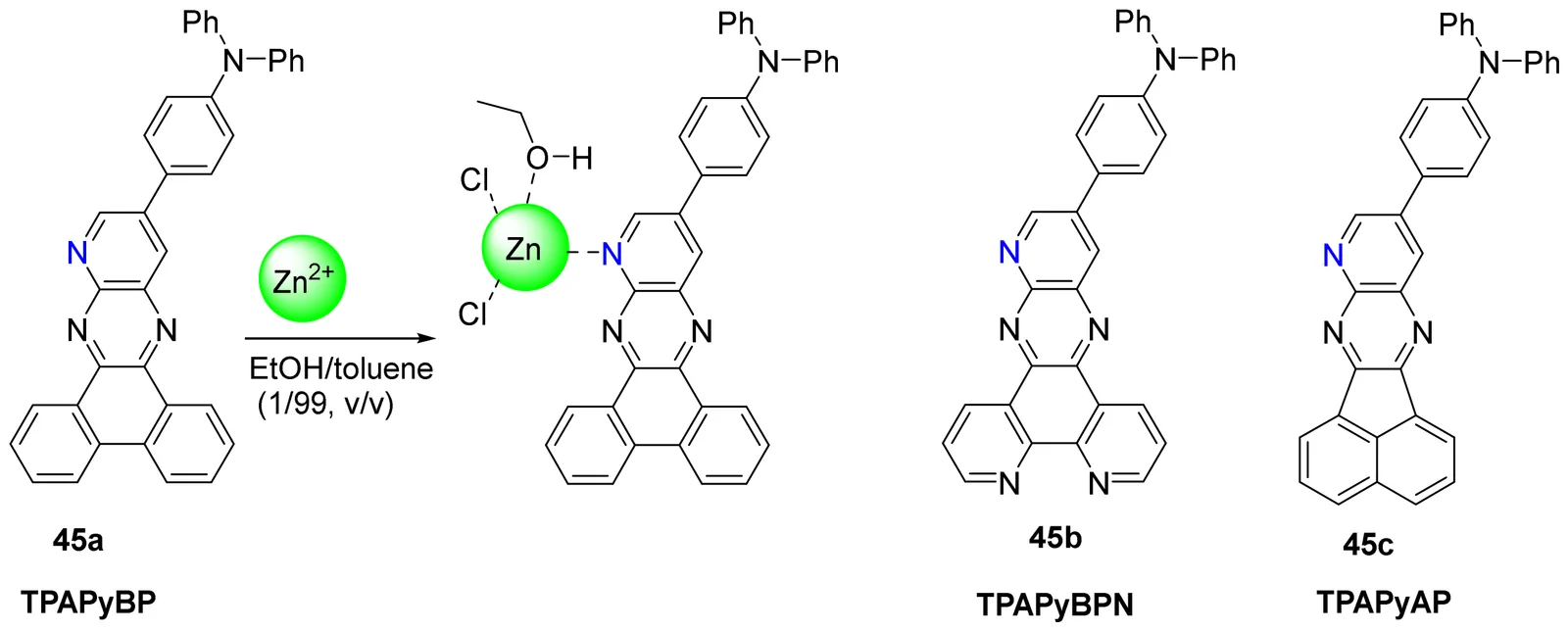
Molecular structure of pyridopyrazine derivatives **45a**–**c** and interaction pattern of **45a** with ZnCl_2_. Color coding: nitrogen atom involved in metal coordination are shown in blue.

Si and co-workers [105] introduced a family of three donor–acceptor emitters **45a**–**c** (TPAPyAP, TPAPyBP, and TPAPyBPN, Figure 40) with central pyrido[2,3-*b*]pyrazine core, exhibiting a distinctive “two-color” response to ZnCl_2_, accompanied by an emission shift from green to deep red.

**Figure 41:**
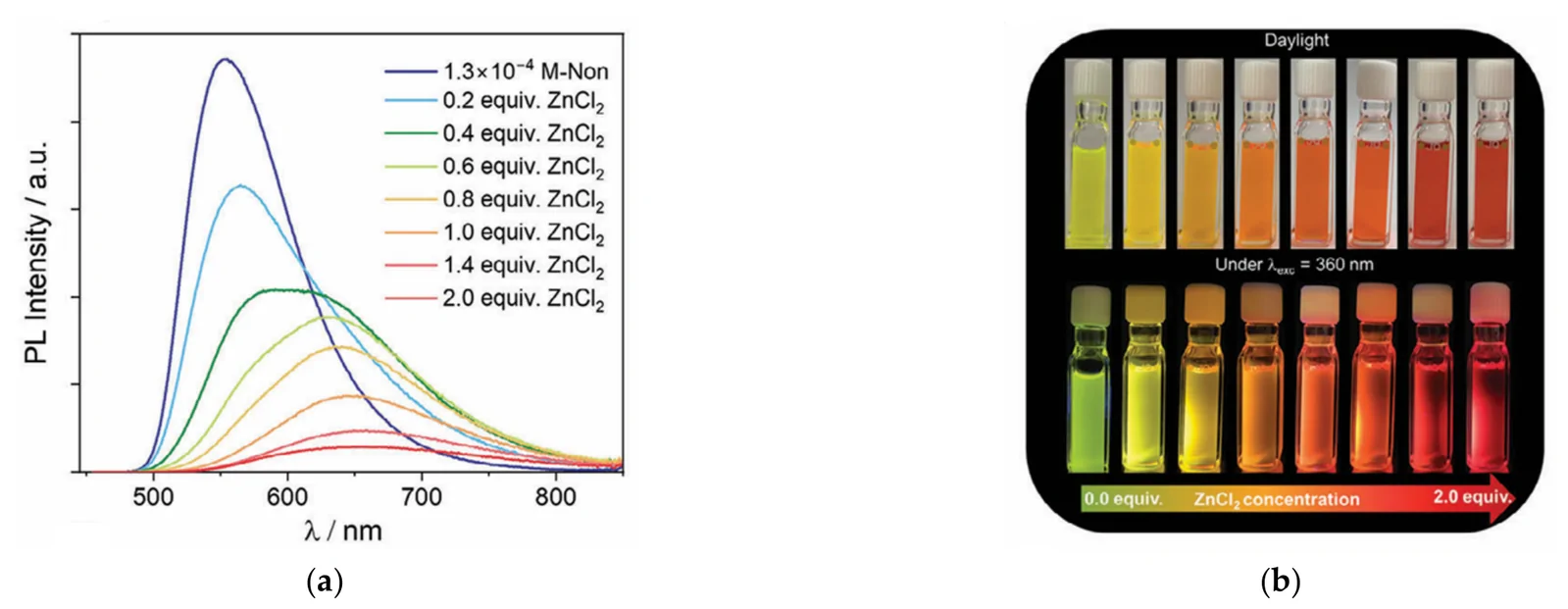

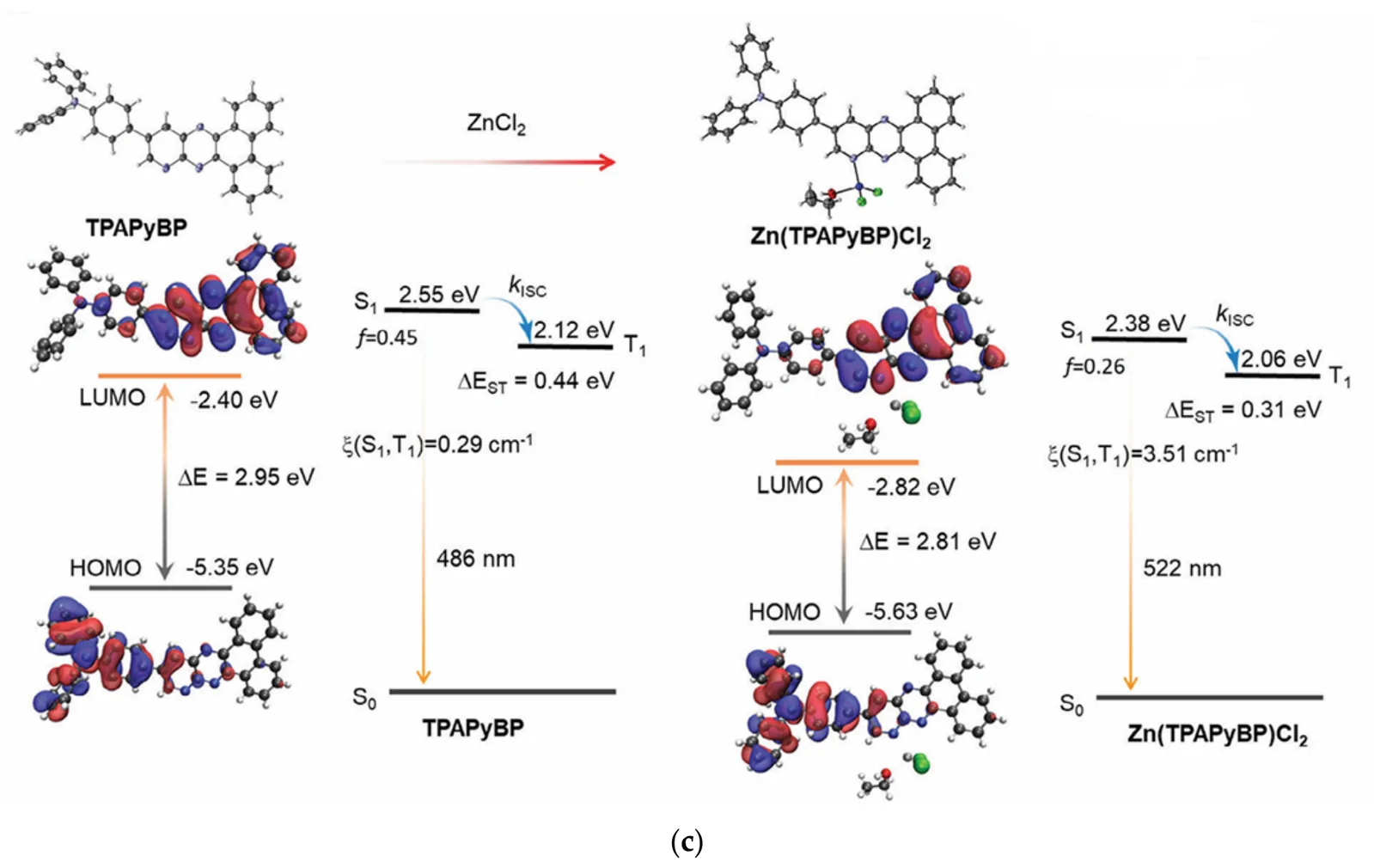
(**a**) PL measurements of **45a**, TPAPyBP (1.3 × 10^−4^ M) with the addition of ZnCl_2_ from 0 to 2.0 equiv. (*λ*_ex_ = 487 nm); (**b**) Samples in daylight and excited by UV torch (*λ*_ex_ = 360 nm) of **45a**, TPAPyBP ZnCl_2_ from 0 to 2.0 equiv.; (**c**) Combined view of the single crystal structures of **45a** and **its Zn^2+^-complex** (TPAPyBP and Zn(TPAPyBP)Cl_2_), and the corresponding frontier molecular orbitals (isovalue: 0.02) calculated using single crystal geometry in the gas phase at the PBE0/6-31G(d,p) level. Reproduced with permission of Wiley-VCH GmbH under the terms of the Creative Commons CC BY license [105].

This behavior distinguishes them from conventional sensors that rely solely on intensity changes. Among the series, the phenanthroline derivative **45b** shows the strongest electron-accepting capability (LUMO = −3.25 eV vs. −3.13 eV for **45a**) and the most red-shifted emission in toluene (575 nm). Nevertheless, **45a** demonstrated superior analytical performance then **45b** and **45c**, including the most pronounced bathochromic shift (from 550 to 680 nm (Figure 41a,b)) and a rapid response time on the order of seconds [104]. Mechanistically, ZnCl_2_ binding stabilizes the LUMO of the acceptor and modulates the TADF/ICT emissive state, producing a green-to-deep-red two-color response that is distinct from conventional intensity-based sensing.

The authors attribute these results to the optimal balance of properties offered by the dibenzo[*f*,*h*]-pyridopyrazine acceptor. Efficient sensor operation requires sufficiently strong binding for complex formation, yet coordination must proceed rapidly without irreversible degradation of the emitter. In **45b**, the excessively high coordination ability arising from additional chelating nitrogen atoms in the rigid phenanthroline framework apparently leads to over-stabilization of the complex. Consequently, while a spectral response is observed in **45b**, it is less contrast-rich compared to **45a**. For **45b**, a new band appears at ~655 nm, but the spectral changes are less dramatic, likely because the exceptionally strong dibenzo[*f*,*h*]-pyridopyrazine acceptor already substantially stabilizes the charge-transfer state, so additional zinc coordination introduces smaller relative perturbations in electron density distribution.

Structural factors also play a key role: the dibenzophenazine fragment in **45a** provides an optimal torsional angle between donor and acceptor moieties (approximately 31° for one independent molecule in the crystal and nearly coplanar for the other), and upon ZnCl_2_ binding, the geometry rearranges to effectively stabilize the LUMO (by 0.42 eV according to calculations) (Figure 41c). In contrast, the more rigid pyridino-phenanthroline core of **45b** restricts conformational flexibility during complexation; although the LUMO is also stabilized, the change in the optical gap (ΔE) is insufficient to produce a comparable bathochromic shift. Furthermore, the abundance of coordination-active nitrogen atoms in dibenzo[*f*,*h*]-pyridopyrazine may compromise selectivity, rendering the sensor more responsive to other Lewis’s acids such as SnCl_2_. In **45a**, however, the pyridinic nitrogen of the pyrazine ring together with the N-atoms of the phenazine moiety strike an optimal balance, enabling selective ZnCl_2_ binding with 1:1 stoichiometry and yielding maximum spectral contrast [105].

The coordination mode of the ligand **45a** with ZnCl_2_ was confirmed by single-crystal X-ray diffraction analysis of **45a**-Zn^2+^ complex. Theoretical calculations (TD-DFT at the PBE0/6-31G(*d*,*p*) level) not only predicted the electron density distribution in the excited states but also revealed that complexation lowers the LUMO energy by 0.42 eV, which is consistent with the emergence of new long-wavelength absorption and emission bands; however, orbital energy differences should not be equated directly with experimental transition energies. The combination of experimental techniques (NMR titration, mass spectrometry) with quantum-chemical modeling provided a consistent picture of the sensing mechanism as chemisorptive binding of the analyte with formation of a 1:1 stoichiometric adduct. The detection limit for ZnCl_2_ was estimated by the authors [105] to be approximately 5.0 × 10^−5^ M (50 µM).

Pyrido[2,3-*b*]pyrazines, bearing an additional pyridine nitrogen atom, represent the most electron-deficient annelated pyrazine system discussed in this review. This enhanced electron-accepting character, combined with the presence of multiple coordination-active nitrogen atoms, makes them particularly attractive for the development of chromogenic and fluorogenic sensors for soft and borderline metal ions such as Hg^2+^, Sn^2+^, and Zn^2+^. The reported systems display a notable diversity of functions: from simple colorimetric sensors to AIEE-active probes with solvatochromic properties and cell-imaging capabilities, and even photochromic systems capable of dual-controlled fluorescence switching. The work by Si et al. on donor–acceptor emitters **45a**–**c** is particularly noteworthy, as it demonstrates how structural tuning can achieve a distinctive ‘two-color’ response to ZnCl_2_, with a bathochromic shift from green to deep red. Despite these advances, the number of pyridopyrazine-based sensors is still modest, and further research is warranted to fully exploit the potential of this scaffold for selective metal-ion detection, particularly in biological and environmental applications.

## 4. Conclusions and Outlook

This review has surveyed the structural diversity, recognition strategies, photophysical response mechanisms, and practical applications of pyrazine-based small-molecule chemosensors for metal cations, with an emphasis on studies published between 2015 and 2026. The collected data demonstrate that pyrazine derivatives have evolved from simple short-wavelength emitters to sophisticated multifunctional conjugates capable of orange-to-red or even far-red emission, solvatochromism, aggregation-induced emission (AIE), and bioimaging applications. This evolution reflects the broad synthetic versatility of the pyrazine scaffold, which can be readily functionalized with diverse donor groups, hydrazide/hydrazone units, annulated rings, or π-extended aromatic systems to achieve tailored photophysical properties and metal-ion selectivity. Several key trends emerge from the literature. First, the majority of reported sensors operate through well-established mechanisms, including CHEF, PET, ICT, ESIPT, and, to a lesser extent, CHEQ, FRET, and AIE. The choice of mechanism is often dictated by the structural motif: hydrazone- and carboxamide-based sensors predominantly exploit CHEF and PET upon metal coordination, while annulated systems frequently engage ESIPT or AIE pathways. For clarity, the sensing performance and the exact mechanism of each sensor discussed in this review are summarized in Table 1. A more comprehensive comparative dataset, including solvent composition, pH and buffer, probe concentration, working range, response time, interferents, binding stoichiometry with supporting evidence, and applications in relevant matrices, is provided in Table S1 (Supplementary Materials). Where a given parameter was not reported in the original study, the entry is marked “n.r.” (not reported).

**Table 1:** Sensing mechanisms of pyrazine-based chemosensors for metal cations.

Probe	Analyte	Proposed Mechanism	Method (Response)	Medium, LOD	Application	Ref.
***Pyrazinamides***						
**1**	Zn^2+^	CHEF	Fluorometric (turn-on)	CH_3_CN: 1.11 µM	INHIBIT logic gate	[55]
**1**	Cu^2+^	ICT/LMCT	Colorimetric	CH_3_CN: 14.8 µM	-	[55]
**2**	Cu^2+^	CHEQ (paramagnetic quenching)	Fluorometric (turn-off)	DMSO: 5.73 µM	Human plasma, drinking water, test paper strips, molecular logic function	[56]
**2**	Pb^2+^	CHEQ (heavy-atom effect)		DMSO: 17.4 µM		[56]
**3a,b**	Fe^3+^	CHEQ (paramagnetic quenching)	Colorimetric and Fluorometric (turn-off)	DMSO: **3a**: 11.7 µM (FL) **3b**: 11.6 µM (FL)	Real water samples, test paper strips	[57]
**4a**	Cu^2+^	CHEQ	Colorimetric and Fluorometric (turn-off)	MeCN: 174 µM (abs) 188 µM (FL)	Test paper strips	[58]
**4b**	Cu^2+^	LMCT; CHEQ	Colorimetric	MeCN: 11 µM (abs)	Test paper strips, biomedical and biosensing field	[59]
**4c**	Cu^2+^	CHEQ	Colorimetric and Fluorometric (turn-off)	MeCN: 0.134 µM (abs) DMSO: 0.502 µM (FL)	Smartphone, test paper strips, biomedical and biosensing field	[60]
***Pyrazinehydrazides***						
**5**	Cu^2+^	PET off; CHEF	Colorimetric and Fluorometric (turn-on)	DMSO/H_2_O (1:1): 12.1 nM (FL)	Real water samples	[61]
**6**	Cu^2+^	ICT	Colorimetric	MeOH: 390 nM	Real water samples	[62]
**7**	Cu^2+^	CHEQ	Colorimetric and Fluorometric (turn-off)	MeOH: 3.4 µM (abs) 42 µM (FL)	-	[63]
**8**	Zn^2+^	AIRE	Fluorometric (ratiometric)	H_2_O/DMSO (99.5:0.5): 3.52 µM	Recognition Zn^2+^ and PPi in living cells	[64]
**9**	Zn^2+^	ICT	Colorimetric	CH_3_CN/H_2_O (7:3): 136 nM	Pharmaceutical and real water samples, test paper strips, smartphone	[65]
**10**	Al^3+^	ESIPT off; PET off; CHEF	Fluorometric (turn-on)	DMSO: 15.6 nM DMSO/HEPES buffer (8:2): 33.8 nM	Determination of Al^3+^ and F^−^ in living cells	[66]
**10**	Mg^2+^			DMSO: 80.4 nM	-	[66]
**10**	Zn^2+^			DMSO: 162.3 nM	-	[66]
**11**	Al^3+^	PET off; ESIPT off; rigidification	Fluorometric (turn-on)	MeCN: 16.6 nM (FL)	Real water samples	[67]
**12**	Al^3+^	CHEF; AIE	Fluorometric (turn-on)	H_2_O/DMSO (99:1): 8 nM (FL)	Real water samples	[68]
**13**	Ag^+^	CHEF; restriction of C=N isomerization	Colorimetric and Fluorometric (turn-on)	DMSO/H_2_O (7:3): 10.6 nM (abs) 6.74 nM (FL)	Real water sample, paper test strips, bioimage	[69]
**14**	Al^3+^	ICT off; imine bond hydrolysis	Chromogenic and Fluorometric (turn-on)	CH_3_CN/H_2_O (9:1): 137 nM	Bioimaging	[70]
***2-Acetylpyrazines***						
**17a**	Al^3+^	CHEF; PET off; rigidification	Fluorometric (turn-on)	EtOH: 100 nM	-	[71]
**17b**	Al^3+^	CHEF; PET off; rigidification	Fluorometric (turn-on)	EtOH: 100 nM	-	[72]
**18**	Co^2+^	CHEF; ICT; restriction of C=N isomerization	Colorimetric and Fluorometric (turn-on)	DMSO: 0.104 nM (FL)	Test paper strips, real water samples, imaging of exogenous Co^2+^ in HeLa cells	[73]
**19**	Co^2+^	CHEF; spirolactam ring opening	Colorimetric and Fluorometric (turn-on)	THF/Tris-HCl (4:6, pH 7.5): 0.31 µM (FL)	Imaging Co^2+^ in living cells	[74]
***Aminopyrazines***						
**20**	Cr^3+^	Coordination-induced hyperchromic effect	Colorimetric	MeOH: 0.77 µM	AND molecular logic gate	[44]
**21**	Hg^2+^	LMCT; CHEF; restriction of C=N isomerization	Colorimetric and Fluorometric (turn-on)	MeOH/H_2_O (3:7): 26.4 nM	Real water samples, molecular logic gate	[77,78]
**21**	Ni^2+^		Colorimetric and Fluorometric (ratiometric)	MeOH/H_2_O (7:3): 0.862 µM	Real water samples, test strips, fluorescence imaging of Hg^2+^ in HeLa cells, molecular logic gate	[77,78]
**22**	Al^3+^	ESIPT; ICT	Colorimetric	CH_3_CN/H_2_O (1:1): 1.7 µM	Test paper strips, molecular logic gate	[79]
**23**	Cu^2+^	ESIPT off; CHEQ (paramagnetic quenching)	Colorimetric and Fluorometric (turn-off)	THF: 24.7 nM (FL)	Real water samples, test paper strips	[80]
***Hydrazinopyrazines***						
**24**	Al^3+^	MLCT; CHEF; PET off	Fluorometric (turn-on)	EtOH/H_2_O (95:5): 233 nM	Water and drug samples, sensor-coated swabs, molecular logic gates	[81]
**24**	Zn^2+^			EtOH/H_2_O (95:5): 168 nM		[81]
***Arylpyrazines***						
**25**	Fe^3+^	CHEQ (paramagnetic quenching)	Fluorometric (turn-off)	MeCN: 0.63 µM	Real water samples	[82]
**26b**	Fe^3+^	CHEQ (paramagnetic quenching)	Fluorometric (turn-off)	CH_3_CN: No data	-	[83]
**27**	Pb^2+^; K^+^	AIE disruption	Fluorometric (turn-off)	H_2_O/THF (9:1): No data	-	[84]
**28**	Fe^3+^	ICT; FRET	Colorimetric and Fluorometric (turn-off)	DMF/HEPES buffer (3:7): 3.57 µM (abs) 84.6 nM (FL)	-	[85]
***Other substituted pyrazines***						
**29a–c**	Zn^2+^	PET off; CHEF	Fluorometric (turn-on)	Aq. buffer pH 7: No data	Live Cell Imaging	[86,87]
**30a–c**	Cd^2+^	PET off	Fluorometric (turn-off)	THF/H_2_O (1:1): 8–13 µM	-	[88]
	***Pyrazines annelated with five-membered carbo(hetero)cycle***					
**31**	Hg^2+^ (with CH_3_COO^−^/F^−^)	ICT	Colorimetric	EtOH/H_2_O (1:1): No data	AND logic gate	[89]
**32a–c**	Pd^2+^/Pd^0^	ICT	Fluorometric (turn-off)	MeCN: No data	-	[90]
**33a–e, 34a,b**	Lewis-acidic metal ions: Li^+^, Mg^2+^, Ca^2+^, Ba^2+^, Sc^3+^, La^3+^	Imidazopyrazinium resonance form stabilization	Colorimetric and Fluorometric	MeCN: No data	-	[91,92]
**35a,b**	Hg^2+^	Electron/energy-transfer	Fluorometric (turn-off)	THF: No data	-	[93]
***Quinoxalines***						
**36**	Fe^3+^	-	Colorimetric	MeOH/H_2_O (1/1): 0.378 µM	Tap water	[95]
**37**	Sb^3+^	-	Colorimetric	TAC-TEHP membrane: 20.5 nM	Real water samples, food, biological effluents	[96]
**38**	Zn^2+^	ESIPT off; PET off; restriction of C=N isomerization; CHEF	Fluorometric (turn-on)	EtOH/HEPES buffer (8:2, pH 7.4): 95 nM	-	[97]
**39**	Fe^3+^	static quenching due to ground-state complexation	Colorimetric and Fluorometric (turn-off)	MeOH: 3.58 µM (FL)	Real water samples	[98]
**40**	Cu^2+^	ICT	Colorimetric	MeOH/HEPES buffer (1:1, pH 7.4): 2.3 µM	Real water sample, paper test strips, molecular logic gate	[99]
**41**	Fe^3+^, Fe^2+^, Co^2+^, Ni^2+^, Cu^2+^, Zn^2+^, Pb^2+^, Ag^+^, Cd^2+^, Hg^2+^	Multiple coordination sites; ICT	Colorimetric	DMSO/H_2_O (8:2): 3.94–0.08 µM (lowest for Cd^2+^)	Paper test strips,	[100]
***Pyrido[2,3-b]pyrazines***						
**42**	Co^2+^, Cu^2+^, Zn^2+^, Ni^2+^, Cd^2+^	CHEF	Colorimetric	MeCN: No data	-	[102]
**43**	Hg^2+^	AIEE; PET/energy-transfer on	Colorimetric and Fluorometric (turn-off)	MeCN: 7.46 µM (FL)	Bioimaging	[103]
**44a,b**	Hg^2+^	ESIPT off	Colorimetric and Fluorometric (turn-off)	MeCN: **44a**: 498 nM (abs), 4.53 µM (FL) **44b**: 1.56 µM (abs)	Logic circuit	[104]
**44a,b-** **Hg^2+^**	Sn^2+^	ESIPT off; diamagnetism property of Sn^2+^	Colorimetric and Fluorometric (turn-on)	**44a**: 154 nM (abs), 5.44 µM (FL) **44b**: 36.4 nM (abs)		
**45a–c**	ZnCl_2_	ICT	Colorimetric and Fluorometric (ratiometric)	EtOH/toluene (1:99): 50 µM (FL)	-	[105]

### 4.1. Advantages of the Pyrazine Scaffold

Analysis of the data summarized in Table 1 allows the advantages of pyrazine-based chemosensors to be formulated more precisely than simply as “electron deficiency and synthetic versatility”. First, the two adjacent nitrogen atoms in the 1,4-diazine ring create a compact, polar, and strongly electron-accepting chelation site that is geometrically distinct from the nitrogen donors of bipyridines, terpyridines, or phenanthrolines: the N–N distance in pyrazine is too short for direct bidentate chelation of a single metal ion but is ideally suited for bridging coordination, hydrogen bonding, and participation in extended chelate pockets when combined with amide, hydrazide, or Schiff-base substituents. Second, the low-lying π* orbitals (pKa ≈ 0.65 for the parent ring) make the pyrazine core an intrinsic electron acceptor, which stabilizes ICT states, narrows the HOMO–LUMO gap upon metal coordination, and facilitates the red shift in absorption and emission that is essential for biological imaging. Third, the pyrazine ring is synthetically more accessible than its annulated analogs: it can be functionalized at the 2-, 3-, 5-, and 6-positions by nucleophilic substitution, cross-coupling, or condensation, whereas quinoxalines and pyrido[2,3-*b*]pyrazines require more elaborate building blocks. Fourth, the pyrazine moiety plays a dual role—it acts simultaneously as a coordination site, an electron acceptor, and a modulator of photophysical pathways (CHEF, CHEQ, PET, ICT, ESIPT, AIE)—which is not typical of conventional fluorophores such as rhodamine, BODIPY, or pyrene, where the signaling unit is electronically decoupled from the receptor. This dual role explains why a single pyrazine scaffold can be tuned to respond to chemically diverse cations (Zn^2+^, Cu^2+^, Fe^3+^, Al^3+^, Hg^2+^, Ag^+^, Co^2+^, Ni^2+^, Cr^3+^, Cd^2+^, Pd^2+^, Sb^3+^, La^3+^) simply by varying the peripheral substituent.

The analysis also reveals a clear dichotomy between sensors designed for short-wavelength detection (typically absorbing below 400 nm and emitting in the blue–green region) and those engineered for long-wavelength or far-red emission. The latter, often achieved through annulation (quinoxalines, pyridopyrazines) or conjugation with strong donor groups, are particularly promising for biological applications owing to reduced autofluorescence and enhanced tissue penetration. Several such systems have been successfully validated in live-cell imaging, demonstrating low cytotoxicity and effective intracellular metal-ion detection.

The coordination chemistry of pyrazine-derived ligands reveals considerable diversity. While many sensors incorporate N,N,O-tridentate chelating pockets (particularly in hydrazone and Schiff-base derivatives), the pyrazine ring nitrogen atom does not invariably participate in metal binding. In certain systems, particularly those with strongly chelating substituents (such as salicylidene hydrazones), the pyrazine moiety may remain uninvolved, with coordination occurring exclusively through the peripheral donor groups. Conversely, in aryl- and hetaryl-substituted pyrazines, the ring nitrogen atoms often serve as the primary coordination sites, highlighting the importance of the substitution pattern in determining the binding mode.

Beyond the small-molecule sensors discussed above, recent work has extended the application of pyrazine-containing architectures to functional coordination complexes and portable sensing platforms. For example, a binuclear Cd(II) complex bridged by tetra-2-pyridinylpyrazine was shown to act as a dual-mode colorimetric and fluorometric sensor for Fe^3+^, with detection limits of 3.57 µM and 84.6 nM, respectively, and was further integrated into a Schottky diode device [85]. Similarly, quinoxaline-based Schiff base receptors have been applied to the detection of Ag^+^ in environmental samples, BSA proteins, and living cells over a wide pH range [106]. More recently, a nitrogen-rich imidazolylpyrazine derivative has been reported as a “turn-on” fluorescent probe for La^3+^ via ligand-to-metal energy transfer, with a detection limit of 2.32 µM [107], highlighting the potential of pyrazine scaffolds for rare-earth ion sensing and separation. These examples underscore the growing trend towards multifunctional pyrazine-based sensing platforms that combine molecular recognition with device integration, biological imaging, and environmental monitoring.

### 4.2. Critical Limitations and Loopholes

Despite these advantages, the present survey reveals several systemic rather than incidental limitations.

(1) The Cu^2+^/Fe^3+^ problem is structural, not accidental. Because both ions are paramagnetic (d^9^ and d^5^, respectively) and possess accessible redox couples, they quench fluorescence through multiple parallel pathways (ISC, heavy-atom effect, electron transfer, inner-filter effects). Any pyrazine-based sensor that relies on a nitrogen-rich chelating pocket will inevitably bind Cu^2+^ and Fe^3+^ with high affinity; selectivity can therefore be achieved only by steric or electronic discrimination (e.g., size-selective cavities, rigidified chelates, or redox-inactive analogs), not by simply increasing binding strength.
(2) “Turn-on” sensing of paramagnetic ions remains the exception. Only a handful of sensors (**18** for Co^2+^, **12** for Al^3+^) achieve genuine turn-on responses towards paramagnetic analytes, and the mechanistic origin of this behavior—efficient radiative channeling by rigid fluorophores—is not yet generalizable.
(3) Binding stoichiometries are often inferred from indirect methods. Job’s plots, Benesi–Hildebrand analyses, and ESI-MS provide evidence consistent with a proposed binding model, but they do not establish stoichiometry unequivocally: their interpretation depends on assumptions about species distribution, concentration regimes, and the possible coexistence of multiple equilibria. Only a minority of the sensors discussed (such as **4a–c, 38, 45a**) have been characterized by single-crystal XRD of the metal complex, which provides direct structural evidence. This limitation should be kept in mind when comparing reported stoichiometries across different studies.
(4) Crystallographic characterization is the exception rather than the rule. Of the sensors discussed, only a minority (e.g., **4a**–**c, 38, 45a**) have been characterized by single-crystal XRD of the metal complex; and even fewer have been studied by equilibrium methods capable of yielding binding constants or free energies of association. In most cases the binding mode is inferred from Job’s plots, ESI-MS, and DFT. Furthermore, the thermodynamic stability of the complex is inferred from the HOMO–LUMO gap, an inference that is not, by itself, justified, since the orbital energy gap is not a measure of binding affinity. This gap hampers the rational design of truly orthogonal sensors and should be addressed in future work by combining structural, thermodynamic, and photophysical characterization.
(5) The reported analytical performance is not directly comparable. Detection limits, association constants, and selectivity coefficients are measured under widely different conditions (solvent, pH, ionic strength, competing ions, excitation wavelength), and no standard protocol exists for pyrazine-based sensors; this hampers the identification of genuinely superior designs.
(6) Biological validation is still superficial. Most “bioimaging” claims are based on HeLa or MCF-7 cells incubated with the sensor for a few hours; long-term cytotoxicity, cellular localization, and in vivo behavior remain largely unexplored.
(7) The gap between laboratory demonstration and practical deployment is wide. Test-strip and smartphone-based readouts are promising, but issues of reproducibility, storage stability, matrix effects, and regulatory validation have not been systematically addressed. Taken together, these limitations explain why, despite the impressive number of published pyrazine-based sensors, very few have reached the stage of validated analytical protocols or commercial devices.

### 4.3. Future Development Directions

The mechanistic map presented in Table 1 suggests several concrete directions rather than generic calls for further research.

(1) Rational design of Cu^2+^/Fe^3+^-discriminating cavities. This requires moving beyond the currently dominant N,N,O-tridentate motif towards size-selective or redox-tuned chelates, for example, by introducing bulky substituents that penalize the smaller Cu^2+^ ion, or by incorporating redox-active groups that shift the Fe^3+^/Fe^2+^ potential out of the sensing window.
(2) Exploitation of TADF-active pyrido[2,3-*b*]pyrazines. The two-color ZnCl_2_ response of **45a–c** suggests that thermally activated delayed fluorescence can be modulated by coordination; extending this principle to ratiometric sensing of other cations, and to time-resolved detection (which would eliminate background fluorescence), is a promising but almost unexplored direction.
(3) Deliberate combination of two or more mechanisms. Sensors that integrate ESIPT + AIE, FRET + ICT, or PET + ESIPT within a single pyrazine scaffold could provide orthogonal readouts (e.g., ratiometric intensity plus lifetime), improving reliability in complex matrices.
(4) Integration into portable platforms. Smartphone-assisted RGB analysis, paper-based test strips, microfluidic devices, and wearable sensors are natural next steps; however, they require close collaboration between synthetic chemists, analytical chemists, and engineers, and must address reproducibility and shelf-life.
(5) Pyrazine-based MOFs and COFs. Although MOF-based sensors were deliberately excluded from this review, the pyrazine linker is one of the most versatile building blocks for porous sensing materials; a systematic comparison of discrete pyrazine sensors with their framework-incorporated counterparts would clarify whether porosity offers genuine advantages in selectivity and sensitivity.
(6) Computational pre-screening. DFT and TD-DFT calculations have already proven indispensable for rationalizing experimental observations; their systematic use in the pre-screening of candidate structures—particularly for predicting HOMO–LUMO gaps, ICT character, and binding affinities—could substantially accelerate the discovery of next-generation sensors.
(7) Biocompatibility and in vivo validation. Moving from cell imaging to in vivo applications will require systematic toxicity studies, pharmacokinetic profiling, and the development of sensors that operate in the near-infrared window where tissue penetration is maximal and autofluorescence is minimal.

Systematic structure–mechanism–performance correlations, supported by crystallography and TD-DFT, remain the key prerequisite for the next generation of pyrazine-based chemosensors. Equally important is the adoption of standardized reporting protocols—specifying solvent, pH, ionic strength, competing ions, excitation wavelength, and the method used to determine detection limits—so that sensors from different laboratories can be compared meaningfully.

### 4.4. Concluding Remark

In summary, pyrazine derivatives have firmly established themselves as versatile and tunable platforms for metal-cation sensing, with notable achievements in environmental monitoring, biological imaging, and molecular logic operations. Their advantages—a compact electron-deficient chelation site, synthetic accessibility, a dual receptor/modulator role, and mechanistic versatility—are now well documented. However, the translation of these laboratory successes into commercial devices and clinical tools requires continued efforts in selectivity enhancement (particularly towards Cu^2+^ and Fe^3+^), crystallographic characterization of binding modes, standardization of analytical protocols, biocompatibility optimization, and device integration. We hope that this comprehensive overview, together with the mechanistic map in Table 1, will stimulate further research and facilitate the rational design of more effective, selective, and practical pyrazine-based chemosensors.

## Data Availability

No new data were created or analyzed in this study. Data sharing is not applicable to this article.

## Supplementary Materials

The following supporting information can be downloaded at: https://www.mdpi.com/article/10.3390/molecules31193573/s1, Table S1: Comparative analytical performance of pyrazine-based chemosensors for metal cations.

## Abbreviations

The following abbreviations are used in this manuscript:

OLED	Organic light-emitting diode
MOF	Metal–organic framework
CHEF	Chelation-enhanced fluorescence
PET	Photoinduced electron transfer
ICT	Intramolecular charge transfer
ESIPT	Excited-state intramolecular proton transfer
WHO	World health organization
DNA	Deoxyribonucleic acid
AIE	Aggregation induced emission
NAC	Nitroaromatic compounds
TNT	Trinitrotoluene
NMR	Nuclear magnetic resonance
ESI-MS	Electrospray ionization mass spectrometry
FT-IR	Fourier-transform infrared spectroscopy
DFT	Density functional theory
TD-DFT	Time dependent density functional theory
HOMO	Highest occupied molecular orbital
LUMO	Lowest occupied molecular orbital
HRMS	High-resolution mass spectrometry
CHEQ	Chelation-enhanced quenching
LOD	Limit of detection
EDTA	Ethylenediaminetetraacetic acid
ISC	Intersystem crossing
UV-vis	Ultraviolet–visible
LMCT	Ligand-to-metal charge transfer
RSD	Relative standard deviation
AIRE	aggregation-induced ratiometric emission
ACQ	Aggregation-caused quenching
LOQ	Limit of quantification
BODIPY	Boron dipyrromethene
US EPA	U.S. environmental protection agency
TPE	Tetraphenylethilene
AIEE	Aggregation-induced emission enhancement
RIR	Restriction of intramolecular rotation
HEPES	N-2-hydroxyethylpiperazine-N′-2-ethanesulfonic acid
FRET	Förster resonance energy transfer
