# Supplementary material for: Pyrazine-Based Chemosensors for Cation Detection
Source: Molecules. 2026 Oct 8;31(19):3573. doi: 10.3390/molecules31193573 (PMC13667247; doi:10.3390/molecules31193573)
Supplement: Supplementary file 1 [file molecules-31-03573-s001.zip › molecules-4591943-supplementary.pdf]

Table S1. Comparative analytical performance of pyrazine-based chemosensors for metal cations

Sensor	Ref. (year)	Structural class	Analyte	Mechanism	Optical response	LOD	Medium (solvent / pH)	Binding stoichiometry and evidence method	Applications
**1**	[55] (2017)	Pyrazinamide (quinoline-linked)	Zn^{2+}; Cu^{2+}	CHEF + ICT (Zn^{2+}); ICT/LMCT (Cu^{2+})	Turn-on (Zn^{2+}, 476 nm); colorimetric, bathochromic shift 328→378 nm (Cu^{2+})	1.11 µM (Zn^{2+}); 14.8 µM (Cu^{2+})	MeCN; pH n.r.	1:1; Job's plot, ESI-MS (m/z 396.74), DFT/TD-DFT	n.r. (inhibit logic gate with SCN^{-})
**2**	[56] (2025)	Pyrazinamide (N-(pyridin-2-ylmethyl))	Cu^{2+}; Pb^{2+}	CHEQ; paramagnetic quenching/ISC (Cu^{2+}); heavy-atom effect (Pb^{2+})	Turn-off (340 nm, λ_{ex} 281 nm)	5.73 µM (Cu^{2+}); 17.4 µM (Pb^{2+})	Aqueous (DMSO); pH n.r.	1:1; Job's plot, ESI-MS, FT-IR	Drinking water, human blood plasma, paper test strips; memory device
**3a**	[57] (2025)	Pyrazinamide (N-(2-iodophenyl))	Fe^{3+}	CHEQ; paramagnetic quenching (d^5)	Turn-off (349 nm, λ_{ex} 268 nm); colorimetric colorless → yellow	11.7 µM	DMSO; pH n.r.	1:1; Job's plot, ESI-MS (m/z 503.19), Benesi–Hildebrand, Stern–Volmer	Drinking, tap, river water; test strips
**3b**	[57] (2025)	Pyrazinamide (N-(2-bromophenyl))	Fe^{3+}	CHEQ; paramagnetic quenching (d^5)	Turn-off (346 nm, λ_{ex} 284 nm)	11.6 µM	DMSO; pH n.r.	1:1; Job's plot, ESI-MS, Benesi–Hildebrand	n.r.
**4a**	[58] (2025)	Pyrazinamide (N-(2-methoxyphenyl))	Cu^{2+}	CHEQ; d–d/LMCT; paramagnetic quenching	Turn-off (514 nm, λ_{ex} 283 nm); colorimetric pale yellow	174 µM (UV-vis); 188 µM (FL)	MeOH / aqueous; pH n.r.	Depends on Cu salt (1:1, 2:1); XRD, Job's plot	Test strips; reversible with EDTA (3–5 cycles)
**4b**	[59] (2025)	Pyrazinamide (N-(2-ethoxyphenyl))	Cu^{2+}	CHEQ; d–d/LMCT; paramagnetic quenching	Turn-off; colorimetric pale yellow; new band ~458 nm	11 µM (UV-vis)	MeCN; pH n.r.	2:1 (L:Cu^{2+}); XRD ([Cu(**4b**)$_2$(η^2-NO_3)$_2$(H_2O)]), Job's plot	n.r.
**4c**	[60] (2026)	Pyrazinamide (N-(2-phenoxyphenyl))	Cu^{2+}	CHEQ; d–d/LMCT; paramagnetic quenching	Turn-off (357 nm, λ_{ex} 280 nm); colorimetric pale yellow	0.134 µM (UV-vis); 0.502 µM (FL)	MeCN; pH ≈ 6 (broad range)	4:1 (L:Cu^{2+}); XRD ([Cu(**4c**)$_4$$Cl_2$]), Job's plot	Smartphone RGB/test kit; biological studies
**5**	[61] (2026)	Pyrazinehydrazide (N-ethylcarbazole)	Cu^{2+}	PET → CHEF switching; ICT	Turn-on (500 nm); colorimetric colorless → bright yellow	12.1 nM	DMSO/H_2O (1:1, v/v); pH 4–10	1:1; Job's plot, ^1H NMR, DFT (B3LYP/6-31G(d))	Real water samples
**6**	[62] (2021)	Pyrazinehydrazide (chromone)	Cu^{2+}	ICT (enhanced); O,N,O-tridentate	Colorimetric; new band 311→428 nm	390 nM	MeOH; pH 6–11 (optimum 7.9)	1:1; Job's plot, HRMS (m/z 355.9), DFT (B3LYP)	Tap, groundwater, canal water (98–108% recovery, RSD < 2.8%)
**7**	[63] (2022)	Pyrazinehydrazide (1,2,3-triazole-linked organosilane)	Cu^{2+}	CHEQ; energy transfer to Cu^{2+} d-orbitals; heavy-atom effect	Turn-off (420–450 nm); hyperchromic UV-vis shift	3.4 µM (abs); 42 µM (FL); 2.7 µM / 0.86 µM after immobilization	MeOH; pH n.r.	1:1; Job's plot, Benesi–Hildebrand, ^1H NMR	Magnetic nanoparticles (Fe_3O_4@SiO_2); reversible with EDTA
**8**	[64] (2021)	Pyrazinehydrazide (coumarin, AIRE-active)	Zn^{2+}	AIRE; ratiometric; CHEF; O,N,O-coordination	Ratiometric (F_{628}/F_{555} ~11-fold); far-red 628 nm	3.52 µM	H_2O/DMSO (99.5:0.5, v/v); pH n.r.	1:1; Job's plot, ^1H NMR, ESI-MS, DFT	HeLa cells; secondary detection of pyrophosphate (LOD 2.45 µM)
**9**	[65] (2024)	Pyrazinehydrazide (2-hydroxy-1-naphthaldehyde)	Zn^{2+}	ICT; CHEF; ratiometric; deprotonation-	Ratiometric; colorimetric colorless → yellow; new band 450 nm	136 nM	MeCN/H_2O (7:3, v/v); pH n.r.	1:1; ^1H NMR, IR, HRMS, Job's plot, DFT	Zincovit tablets and syrup; real water; test strips

Table S1. Comparative analytical performance of pyrazine-based chemosensors for metal cations

Sensor	Ref. (year)	Structural class	Analyte	Mechanism	Optical response	LOD	Medium (solvent / pH)	Binding stoichiometry and evidence method	Applications
				assisted					
**10**	[66] (2019)	Pyrazinehydrazide (salicylidene)	Al^{3+}; Mg^{2+}; Zn^{2+}	ESIPT inhibition + CHEF	Turn-on; multi-color (green 525 nm Al^{3+}; red 600 nm Mg^{2+}; yellow 545 nm Zn^{2+})	15.6 nM (Al^{3+}); 80.4 nM (Mg^{2+}); 162.3 nM (Zn^{2+})	DMSO; DMSO/H_2O (8:2, v/v); pH n.r.	n.r.; ESI-MS, FT-IR, ^1H NMR, DFT	HeLa cells; sequential detection of F^-
**11**	[67] (2021)	Pyrazinehydrazide (diarylethene, photochromic)	Al^{3+}	PET + ESIPT suppression; AIE; rigidification	Turn-on (541 nm, blue shift 30 nm); colorimetric	16.6 nM	MeCN/H_2O (7:3, v/v); H_2O-induced; pH n.r.	1:1; ESI-MS (m/z 710.40), DFT	Real water samples; reversible with Na_2EDTA
**12**	[68] (2024)	Pyrazinehydrazide (vitamin B_6, pyridoxal-5'-phosphate)	Al^{3+}	CHEF + AIE	Turn-on (456 nm, 20-fold)	8 nM	H_2O/DMSO (99:1, v/v); pH n.r.	1:2 (12:Al^{3+}); ^1H NMR, IR, ^{31}P NMR, DFT, MS (m/z 760.99)	Distilled, river, mineral water (86–99% recovery)
**13**	[69] (2025)	Pyrazinehydrazide (thiazole)	Ag^+	CHEF; restriction of C=N isomerization	Turn-on (523 nm); colorimetric pale yellow → blue	10.6 nM (abs); 6.74 nM (FL)	DMSO/H_2O (7:3, v/v); pH n.r.	1:1; ^1H NMR, HRMS, DFT	Tap and lake water (95–102%); paper strips; MCF-7 cells (viability 75–91%)
**14**	[70] (2019)	Pyrazinehydrazide (BODIPY Schiff base)	Al^{3+}	Chemodosimetric hydrolysis of imine; ICT off	Turn-on (green 552 nm); colorimetric pink → yellow	1.37 nM	MeCN/H_2O (9:1, v/v); pH n.r.	n.r. (hydrolysis); fluorescence titration	Live-cell imaging
**17a**	[71] (2015)	2-Acetylpyrazine (quinolone hydrazone)	Al^{3+}	CHEF + inhibition of C=N isomerization	Turn-on (488 nm, ~230-fold)	100 nM	EtOH; pH n.r.	1:1; Job's plot, ^1H NMR, MS	n.r.
**17b**	[72] (2015)	2-Acetylpyrazine (furan hydrazone)	Al^{3+}	CHEF + inhibition of C=N isomerization	Turn-on (517 nm, 164-fold)	100 nM	EtOH; pH n.r.; probe 50 μM	1:1; Job's plot, ^1H NMR, MS	Reversible with Na_2EDTA
**18**	[73] (2020)	2-Acetylpyrazine (pyrene hydrazone)	Co^{2+}	CHEF; inhibition of C=N isomerization; ICT	Turn-on (390 nm); colorimetric colorless → pale yellow	0.104 nM (6.15 ppb)	DMSO; pH n.r.	1:1; FT-IR, HRMS, DFT	Natural water (86–104%); test strips; HeLa cells (viability > 85%)
**19**	[74] (2017)	2-Acetylpyrazine (rhodamine 6G)	Co^{2+}	Chemodosimetric ring opening + CHEF	Turn-on (550 nm, 56-fold); colorimetric pink-orange	0.31 μM	THF/Tris-HCl (4:6, v/v); pH 7.5; probe 10 μM	2:1 (19:Co^{2+}); Job's plot, ESI-MS, spectrofluorimetric titration	HeLa cells
**20**	[44] (2022)	Aminopyrazine (3-amino-2-pyrazinecarboxylic acid)	Cr^{3+}	Coordination-induced hyperchromic effect (not CHEF/CHEQ)	Hyperchromic (250 nm)	0.77 μM	MeOH; pH 6.0–8.0	3:1 (proposed); N,O-bidentate in crystal; XRD, UV-vis	n.r.
**21**	[77,78] (2018, 2020)	Aminopyrazine (phthalaldehyde Schiff base)	Hg^{2+}; Ni^{2+}	CHEF; LMCT; suppression of C=N isomerization	Hg^{2+}: turn-on, 393→437 nm; Ni^{2+}: ratiometric (393/634 nm); colorimetric	26.4 nM (Hg^{2+}); 0.862 μM (Ni^{2+})	MeOH/H_2O (3:7 and 7:3, v/v); pH n.r.	1:1; Job's plot, IR, DFT	Tap, river, bottled water (72.5–108.3%); test strips; silica gel kits; HeLa cells; logic gates
**22**	[79] (2023)	Aminopyrazine (2-hydroxynaphthalen-1-yl)	Al^{3+}	ESIPT + ICT (monodentate coordination)	Colorimetric colorless; absorption shift 435/457→318 nm	1.7 μM	MeCN/H_2O (1:1, v/v); physiological	1:1; Job's plot, MS, ^1H NMR, DFT	Test strips; logic gate

Table S1. Comparative analytical performance of pyrazine-based chemosensors for metal cations

Sen-sor	Ref. (year)	Structural class	Analyte	Mechanism	Optical response	LOD	Medium (solvent / pH)	Binding stoichiometry and evidence method	Applications
							pH		
**23**	[80] (2020)	Aminopyrazine (D-π-A, hexafluorocyclopentene)	Cu^{2+}	ESIPT restriction + CHEQ + PET; paramagnetic quenching	Turn-off (629 nm); colorimetric orange → black	24.7 nM	THF; pH n.r.	1:1; Job's plot, ^{1}H NMR, ESI-MS	Test strips; real water (97.2–103.4%)
**24**	[81] (2022)	Hydrazinopyrazine (4-(diethylamino)salicylaldehyde)	Al^{3+}; Zn^{2+}	CHEF; PET suppression; N,N,O-coordination	Turn-on (Al^{3+} 582 nm; Zn^{2+} 542 nm)	0.233 μM (Al^{3+}); 0.168 μM (Zn^{2+})	EtOH/H_2O (95:5, v/v); pH n.r.; probe 50 μM	2:1 (Al^{3+}); 1:1 (Zn^{2+}); Job's plot, MS, UV-vis, NMR, FT-IR	Real water and drug samples; cotton swab naked-eye detection
**25**	[82] (2024)	Arylpyrazine (pyrazine-4-yl)pyrazine	Fe^{3+}	CHEQ/paramagnetic quenching (presumed)	Turn-off (440 nm)	0.63 μM	n.r.; pH n.r.	n.r.; n.r.	Tap and drinking water (92.69–105.49%)
**26b**	[83] (2011)	Arylpyrazine (triazinyl fragment)	Fe^{3+}	CHEQ/paramagnetic quenching	Turn-off (green → reddish-brown)	n.r.	MeCN; pH n.r.	n.r.; n.r.	n.r.
**27**	[84] (2026)	Arylpyrazine (2,6-diphenyl-3,5-di(thiophen-2-yl))	Pb^{2+}; K^{+}	AIE disruption; non-radiative decay	Turn-off (390 nm)	n.r.	H_2O/THF (9:1, v/v); pH n.r.	n.r.; n.r.	n.r.
**28**	[85] (2025)	Arylpyrazine (binuclear Cd^{2+} complex, tetrapyridinylpyrazine)	Fe^{3+}	ICT + FRET; static → dynamic quenching	Dual-mode: colorimetric colorless → pink (520 nm); fluorimetric turn-off	3.57 μM (colorimetric); 84.6 nM (fluorimetric)	DMF/H_2O (3:7, v/v); HEPES buffer	n.r.; Stern–Volmer (K_SV = 1.57 × 10^{5} M^{-1}), XPS, lifetime	Six cycles with EDTA
**29a–c**	[86,87] (2008, 2011)	Other (xanthenone/fluorescein, di(2-picolyl)amine)	Zn^{2+}	Two-step PET suppression + CHEF	Turn-on; two-step fluorescence response	n.r.	Aqueous buffer; pH 7	n.r.; fluorescence titration, competitive studies	Biological systems (mobile Zn^{2+})
**30a–c**	[88] (2018)	Other (carbazole–pyrazine conjugated polymers)	Cd^{2+}	PET-based static quenching	Turn-off	9, 13, 8 μM	THF/H_2O (1:1, v/v); pH n.r.	n.r.; Stern–Volmer (K_SV = 1.8–2.3 × 10^{4} M^{-1})	n.r.
**31**	[89] (2011)	Annulated (ninhydrin-derived)	Hg^{2+} (with CH_3COO^{-}/F^{-})	Coordination-induced ICT; AND logic	Colorimetric purple → blue (535→590 nm)	n.r.	EtOH/H_2O (1:1, v/v); pH n.r.	1:1; ^{1}H NMR, IR, UV-vis	Reversible with tetrabutylammonium iodide
**32a–c**	[90] (2015)	Annulated (1H-imidazo[4,5-b]pyrazine)	Pd^{2+}/Pd^{0}	Charge-transfer quenching	Turn-off	n.r.	MeCN; pH 3–7	1:1; n.r.	n.r.
**33a–e, 34a,b**	[91,92] (2005, 2010)	Annulated (imidazo[1,2-a]pyrazin-3(7H)-ones)	Lewis-acidic metal ions (Li^{+}, Mg^{2+}, Ca^{2+}, Ba^{2+}, Sc^{3+}, La^{3+})	Lewis-acid coordination to carbonyl O; resonance stabilization	Colorimetric/fluorometric; blue shift; QY increase	n.r.	MeCN; pH n.r.	n.r.; n.r.	Quantitative determination based on Lewis acidity
**35a,b**	[93] (2010)	Annulated (thieno[3,4-b]pyrazine D-π-A copolymers)	Hg^{2+}	Electron/energy-transfer quenching	Turn-off (**35a**: 644 nm; **35b**: 546 nm)	n.r.	THF; pH n.r.	n.r.; Stern–Volmer (**35a**: 1.1 × 10^{4}; 35b: 2.1 × 10^{4} M^{-1})	n.r.
**36**	[95] (2025)	Quinoxaline (2,3-bis(6-chloropyridin-2-yl)-6-fluoro)	Fe^{3+}	Coordination-induced hyperchromic effect (purely colorimetric)	Hyperchromic UV-vis (280 nm)	0.378 μM	MeOH; pH 7.4 (stable)	1:1; Job's plot, molar ratio, FT-IR	Tap water (errors < 3.93%)
**37**	[96]	Quinoxaline (azo-derivative,	Sb^{3+}	Coordination-induced	Colorimetric (665 nm)	20.5 nM (2.5	TAC-TEHP	3:1; complex formation	Water, biological

Table S1. Comparative analytical performance of pyrazine-based chemosensors for metal cations									
Sen-sor	Ref. (year)	Structural class	Analyte	Mechanism	Optical response	LOD	Medium (solvent / pH)	Binding stoichiometry and evidence method	Applications
	(2025)	optode)		colorimetric complexation		ng/mL)	membrane; pH n.r.		(blood, urine, kidney, femur), food samples; regeneration with HCl
**38**	[97] (2024)	Quinoxaline (hydrazone, 3-hydrazineylquinoxalin-2(1H)-one)	Zn^{2+}	Multi-mechanistic CHEF (ESIPT block + PET suppression + C=N rigidification)	Turn-on (537 nm); naked-eye yellowish-green	95 nM	EtOH/HEPES (8:2, v/v); pH 7.4; pH 6–12	1:2 (38:Zn^{2+}); XRD ($[Zn_2(38)Cl_3]\cdot 2H_2O\cdot 2.5DMF$), DFT, TD-DFT	Real water; reversible with EDTA
**39**	[98] (2025)	Quinoxaline (1,2,3-triazole conjugate)	Fe^{3+}	Static quenching (ground-state complexation)	Turn-off (472 nm); colorimetric hypsochromic shift	3.58 μM	MeOH; pH 2–12	1:1; Job's plot, temperature-dependent Stern–Volmer, lifetime (1.499 vs 1.503 ns)	River, tap, groundwater, distilled water (71–91%); reversible with EDTA
**40**	[99] (2024)	Quinoxaline (indeno[1,2-b]quinoxalinone hydrazide)	Cu^{2+}	Coordination-induced ICT (carbonyl-O, phenolate-O)	Colorimetric; new band 351→460 nm (yellow)	2.3 μM	MeOH/HEPES (1:1, v/v); pH 7.4; pH 5–10	n.r.; IR, NMR, Job's plot	Real water; paper strips (LOD 5.01–5.5 μM); cysteine and ATP detection
**41**	[100] (2026)	Quinoxaline (thiosemicarbazide conjugate)	Fe^{3+}, Fe^{2+}, Co^{2+}, Ni^{2+}, Cu^{2+}, Zn^{2+}, Pb^{2+}, Ag^{+}, Cd^{2+}, Hg^{2+}	Multi-site coordination + ICT	Colorimetric multi-color (yellow → black/orange/brown); bathochromic shifts up to 470 nm	3.94–0.08 μM (lowest for Cd^{2+})	DMSO/H_2O (8:2, v/v); pH n.r.	1:1 (Fe^{3+}/Fe^{2+}); 2:1 (other metals); Job's plot	Paper-strip visual detection; in MeCN inert toward cations, selective for F^-
**42**	[102] (2006)	Pyrido[2,3-b]pyrazine (2,3-di(1H-2-pyrrolyl))	Co^{2+}, Cu^{2+}, Zn^{2+}, Ni^{2+}, Cd^{2+}	Coordination-induced emission (CHEF-like)	Emission at 496 nm; colorimetric	n.r.	MeCN; pH n.r.	n.r.; XRD (42–Ni^{2+} complex, distorted tetrahedral)	n.r.
**43**	[103] (2019)	Pyrido[2,3-b]pyrazine (TPE-substituted, AIEE)	Hg^{2+}	AIEE + PET/energy-transfer quenching	Turn-off (625 nm, λ_{ex} 380 nm); colorimetric orange-red → dark under UV	7.46 μM	MeCN; pH n.r.; probe 2.0 × 10^{-5} mol/L	1:1; Job's plot, ^1H NMR, MS	Cell imaging (AIEE, low cytotoxicity, solvatochromism)
**44a**	[104] (2021)	Pyrido[2,3-b]pyrazine (diarylethene, photochromic)	Hg^{2+}; Sn^{2+}	Coordination + photochromic switching + displacement	Colorimetric (403 nm yellow); turn-off (560 nm); photochromic switching	0.498 μM (Hg^{2+}, UV-vis); 4.53 μM (FL); 154 nM (Sn^{2+} via displacement)	n.r.; pH n.r.	1:1; UV-vis, fluorescence, ^1H NMR	Combinational logic gate (UV, Hg^{2+}, Sn^{2+})
**44b**	[104] (2021)	Pyrido[2,3-b]pyrazine (diarylethene, photochromic)	Hg^{2+}; Sn^{2+}	Coordination + photochromic switching + displacement	Colorimetric (390 nm); turn-off (555 nm)	1.56 μM (Hg^{2+}); 36.4 nM (Sn^{2+})	n.r.; pH n.r.	1:1; UV-vis, fluorescence, ^1H NMR	Photochromic switching
**45a–**	[105]	Pyrido[2,3-b]pyrazine (D-A	$ZnCl_2$	Coordination-induced	Ratiometric/colorimetric;	~50 μM	EtOH/toluene	1:1; XRD (45a–Zn^{2+}), NMR, MS,	OLED applications

Table S1. Comparative analytical performance of pyrazine-based chemosensors for metal cations									
Sen-sor	Ref. (year)	Structural class	Analyte	Mechanism	Optical response	LOD	Medium (solvent / pH)	Binding stoichiometry and evidence method	Applications
**c**	(2024)	TADF emitters)		ICT + TADF modulation	green → deep red (550→680 nm)		(1:99, v/v); pH n.r.; probe 1.3 × 10^{-4} M	TD-DFT (PBE0/6-31G(d,p))	

n.r. — not reported in the original study
n.d. — not determined
